# Genomic Identification of Founding Haplotypes Reveals the History of the Selfing Species *Capsella rubella*

**DOI:** 10.1371/journal.pgen.1003754

**Published:** 2013-09-12

**Authors:** Yaniv Brandvain, Tanja Slotte, Khaled M. Hazzouri, Stephen I. Wright, Graham Coop

**Affiliations:** 1 Department of Evolution and Ecology & Center for Population Biology, University of California - Davis, Davis, California, United States of America; 2 Department of Evolutionary Biology, Evolutionary Biology Centre, Uppsala University, Uppsala, Sweden; 3 Science for Life Laboratory, Uppsala University, Uppsala, Sweden; 4 Department of Biology, New York University – Abu Dhabi, Abu Dhabi, United Arab Emirates; 5 Department of Ecology and Evolutionary Biology, University of Toronto, Toronto, Ontario, Canada; Centre National de la Recherche Scientifique, France

## Abstract

The shift from outcrossing to self-fertilization is among the most common evolutionary transitions in flowering plants. Until recently, however, a genome-wide view of this transition has been obscured by both a dearth of appropriate data and the lack of appropriate population genomic methods to interpret such data. Here, we present a novel population genomic analysis detailing the origin of the selfing species, *Capsella rubella*, which recently split from its outcrossing sister, *Capsella grandiflora*. Due to the recency of the split, much of the variation within *C. rubella* is also found within *C. grandiflora*. We can therefore identify genomic regions where two *C. rubella* individuals have inherited the same or different segments of ancestral diversity (i.e. founding haplotypes) present in *C. rubella*'s founder(s). Based on this analysis, we show that *C. rubella* was founded by multiple individuals drawn from a diverse ancestral population closely related to extant *C. grandiflora*, that drift and selection have rapidly homogenized most of this ancestral variation since *C. rubella*'s founding, and that little novel variation has accumulated within this time. Despite the extensive loss of ancestral variation, the approximately 25% of the genome for which two *C. rubella* individuals have inherited different founding haplotypes makes up roughly 90% of the genetic variation between them. To extend these findings, we develop a coalescent model that utilizes the inferred frequency of founding haplotypes and variation within founding haplotypes to estimate that *C. rubella* was founded by a potentially large number of individuals between 50 and 100 kya, and has subsequently experienced a twenty-fold reduction in its effective population size. As population genomic data from an increasing number of outcrossing/selfing pairs are generated, analyses like the one developed here will facilitate a fine-scaled view of the evolutionary and demographic impact of the transition to self-fertilization.

## Author Summary

While many plants require pollen from another individual to set seed, in some species self-pollination is the norm. This evolutionary shift from outcrossing to self-fertilization is among the most common transitions in flowering plants. Here, we use dense genome sequence data to identify where in the genome two individuals have inherited the same or different segments of ancestral diversity present in the founders of the selfing species, *Capsella rubella* to obtain a genome-wide view of this transition. This identification of founding haplotypes allows us to partition mutations into those that occurred before and after *C. rubella* separated from its outcrossing progenitor, *C. grandiflora*. With this partitioning, we estimate that *C. rubella* split from *C. grandiflora* between 50 and 100 kya. In this relatively short time frame, an extreme reduction in *C. rubella*'s population size is associated with a massive loss of genetic variation and an increase in the relative proportion of putatively deleterious polymorphisms.

## Introduction

Most flowering plants are hermaphroditic, but many have evolved elaborate mechanisms to avoid self-fertilization and the associated costs of inbreeding [1], [2]. However, an estimated 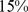 of flowering plant species are predominantly self-fertilizing [3], [4] and many of these species have evolved floral morphologies that promote this means of reproduction. This shift from outcrossing to inbreeding by self-fertilization is among the most common transitions in flowering plants [5], [6], and can occur when the short-term benefits of selfing (e.g. assured fertilization [7], the ‘automatic' transmission advantage [8], and the maintenance of locally adapted genotypes [9]) overwhelm the immediate costs of inbreeding depression [10], [11]. However, in the longer term, limited genetic diversity and difficulty in shedding deleterious mutations are thought to doom selfing lineages to extinction [12]–[14].

While the causes and consequences of plant mating system evolution have long fascinated evolutionary biologists, the paucity of population genomic data for species with a recent shift in mating system and an absence of a framework in which to interpret such data have prevented the development of a genome-wide understanding of this transition. Here, we introduce a novel approach that utilizes patterns of variation in a recently derived selfing population to partition diversity within and among founding haplotypes. By partitioning two sources of sequence diversity – incompletely sorted ancestral polymorphisms and *de novo* mutations which occurred since the population origin – we generate a novel view of the selective and demographic history of a recently derived selfing population. In particular, we can distinguish two factors that can lead to low diversity in selfers: the loss of ancestral polymorphism that occurred at the transition to selfing and a long term small effective population size since the transition.

We apply this framework to the selfing species, *Capsella rubella*, for which we make use of a recently available population genomic dataset [15] consisting of eleven resequenced transcriptomes – six of *C. rubella* and five of a closely related, obligately outcrossing species, *C. grandiflora*, to generate a well-resolved, genome-wide view of the transition from outcrossing to selfing and its immediate consequences. While the origin of *C. rubella* has received significant attention [15]–[18], our understanding of *C. rubella*'s history has been hampered by the small number of independent loci examined in previous studies and by the lack of methods tailored to understand the somewhat unusual haplotype structure of genetic variation within recently derived selfing species. Similarly, while *C. rubella* contains relatively elevated levels of putatively deleterious variation [15]–[17]; previous analyses could not partition the extent to which this was due to a long-term relaxation of the efficacy of purifying selection, or extreme sampling variance at the founding of the species. Perhaps the most intriguing ‘origin story' for *C. rubella* argues that at the last glacial maxima, a single individual capable of selfing may have became isolated and gave rise to the entire species [17]. Evidence for this hypothesis comes from the observation of only one or two distinct haplotypes per a locus in a sample of 17 loci examined in 25 *C. rubella* individuals [17].

Here, we use our novel framework and coalescent modeling to investigate the origin of *C. rubella* focusing on: testing the hypothesis that it was founded by a single individual, estimating the timing of its founding, comparing patterns of variation across its distribution, estimating its long-term effective population size, and documenting the weakening of purifying selection associated with the shift to selfing. A major result of our analyses is that we need not invoke an extreme bottleneck of a single founder, rather the data are consistent with high levels of drift in a population with a small effective size potentially founded by a large number of individuals.

The novel haplotype-based method developed herein allows us to partition polymorphism patterns between variation inherited from the ancestral outcrossing population and new diversity introduced after the bottleneck. By partitioning these sources of variation, our approach allows us to more clearly detail the relaxation in purifying selection associated with the transition to selfing. This partitioning also facilitates coalescent-based approaches to the demographic history of selfing populations and can therefore help infer the extent of a founding bottleneck, identify population subdivision, and document recent population growth and geographic spread. Therefore, beyond the application to *Capsella*, the framework developed here can be used in other pairs of outcrossing/selfing species in order to build a broad comparative view of the shift from outcrossing to self-fertilization. More generally, the ideas developed herein could be applied to many recently diverged species pairs in which one has gone through an extreme demographic bottleneck, leaving only a few recognizable founding haplotypes, regardless of mating system.

## Results

### Samples/Sequencing

#### Sequence data

We analyze SNP data generated from the transcriptomes of 11 *Capsella* samples (six *C. rubella*, five *C. grandiflora*) aligned to the *C. rubella* reference genome [15]. SNPs were called using the GATK pipeline and subjected to an additional series of quality controls (described in the *METHODS*). These calls were validated by comparison to 53 Kb of Sanger sequencing that overlapped a subset of these data, revealing highly replicable genotype calls across technologies and nearly identical values of 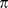 (see *METHODS*, and Table S1). Throughout the paper we focus on detailing variation at four- and zero- fold degenerate sites (i.e. synonymous, and nonsynonymous sites), which we signify with the subscripts, 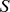 and 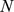 respectively.

Together, our data span 124.6 Mb of the *C. rubella* genome, covering 25,000 unigenes. Of this 124.6 Mb approximately 96% could be assigned a recombination rate from a genetic map (map length = 339 cM) that was constructed from a QTL cross between *C. rubella* and *C. grandiflora* [19]. While this genetic map may not be representative of that in *C. rubella*, it is more appropriate to measure haplotype lengths on a genetic rather than physical map, because the former provides information about the number of outcrossing events since coalescence, and so we quote both measures.

#### Samples

Our six *C. rubella* samples consist of three plants from Greece, the native range of *C. grandiflora*, and the putative location of the origin of *C. rubella* [16], [17], and three from outside of Greece (Italy, Algeria, and Argentina), outside of *C. grandiflora*'s range. We often partition our analysis into these two *C. rubella* groups because Greek samples are likely closer to demographic equilibrium and have the opportunity to introgress with *C. grandiflora*, while Out-of-Greece samples provide us with an opportunity to explore the influence of *C. rubella*'s geographic expansion on patterns of sequence diversity.

### Whole genome summaries

Before presenting our haplotype-based analyses, we briefly summarize patterns of sequence variation within and among species. These results, which are consistent with previous analyses and are strongly concordant with Slotte *et al.*'s [15] analysis of the same data, are summarized here for completeness. To generate empirical confidence intervals, we calculate the upper and lower 2.5% of tails of focal summary statistics by resampling 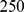 kb blocks with replacement.

#### Patterns of diversity and divergence

In Table 1 and Figure S1A we show variation within and between populations and species. Interspecific divergence at synonymous sites (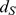) slightly exceeds synonymous diversity (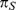) in *C. grandiflora*. In turn, both of these estimates dwarf diversity in *C. rubella*. Sequence diversity in *C. rubella* is geographically structured, with pairs of Out-of-Greece samples being much more similar to one another than are Greek sample pairs (estimated as 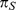), while pairs consisting of one Greek and Out-of-Greece sequence differ slightly more. The spatial structure of genetic variation in *C. rubella* argues against recent introgression between*C. grandiflora* and sympatric Greek *C. rubella*, since divergence (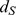) between them is not significantly different from that between allopatric Out-of-Greece *C. rubella* and *C. grandiflora*. To further test this, we calculated a formal test of introgression, the 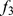 statistic [20], [21], which provided no evidence for introgression (see Text S1).

**Table 1:** Percent sequence variation within and between *Capsella spp*.

		*C. rubella*		
	*C. grandiflora*	Greek	Out-of-Greece	All
*C. grandiflora*	1.86 [1.83,1.93]			
Greek *C. rubella*	2.03 [2.01,2.13]	0.40 [0.39,0.45]		
Out-of-Greece *C. rubella*	2.02 [2.00,2.09]	0.46 [0.45,0.52]	0.27 [0.26,0.32]	
All *C. rubella*	2.03 [2.00,2.11]	NA	NA	0.41 [0.40,0.46]

Neutral variation within and between *Capsella* populations. Percent sequence differences at synonymous sites averaged across pairs of individuals within and between *C. rubella* and *C. grandiflora*. This matrix is symmetric and comparisons between partially overlapping sets (e.g.*C. rubella* x Greek *C. rubella*) are noted as ‘NA’. Redundant cells above the main diagonal are intentionally left blank.

Additional characteristics of these data, specifically an excess of intermediate frequency variants and a relative excess of nonsynonymous variation, likely reflect genomic consequences of the transition to selfing. For example, we observe an excess of intermediate frequency variation in both Greek and Out-of-Greece *C. rubella* samples as compared to constant neutral population expectations, consistent with a historical population contraction (Figure S1C–D). A relaxed efficacy of purifying selection in *C. rubella* is suggested by the level of nonsynonymous relative to synonymous variation within and between species (Figure S1B) – 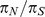 within *C. rubella* is large (0.173) compared to both 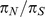 within *C. grandiflora* (0.144), and to 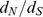 or between species (0.146).

#### The genomes of *C. rubella* individuals are largely autozygous

Since *C. rubella* is predominantly self-fertilizing, we expect most of an individual's genome to be autozygous – that is, an individuals two chromosomes are predominantly identical by descent due to a very recent common ancestor. As expected, most *C. rubella* individuals are homozygous at the majority of sites (*C. rubella* individuals are homozygous at 89% to 95% of non-singleton synonymous polymorphisms in *C. rubella*, as compared to *C. grandiflora* individuals who are homozygous at 55% to 64% of non-singleton synonymous polymorphisms), likely due to numerous consecutive generations of self-fertilization in *C. rubella*. However, some individuals contain a few genomic regions that are putatively allozygous, as manifested by high local levels of heterozygosity. Such regions have yet to be homogenized by selfing since the most recent ancestral outcrossing event, and are clearly demarcated and easily identified by higher levels of individual heterozygosity than in the rest of the genome (see Text S1 and Figures S9A–F). In total, we infer that on average 7% of a *C. rubella* individual's genome is allozygous. To simplify our haplotype-based analyses, we ignore these allozygous regions, which allows us to directly observe the phase of nucleotide variants. In the *METHODS* and Figure S8 we show that these excluded allozygous regions do not contain unusual patterns of sequence diversity, and so their exclusion is unlikely to affect our inference (see *METHODS*).

### Comparisons within and among founding haplotypes

We now describe our novel haplotype-based analysis, which focuses on identifying haplotypes that founded *C. rubella*. By identifying these distinct founding haplotypes, we can divide variants in the extant *C. rubella* population into those present in its founding lineages and new mutations. This information will allow us to infer a coalescent based model of the recent demography of *C. rubella*.

#### Identifying *C. rubella*'s founding haplotypes

**Figure 1:**
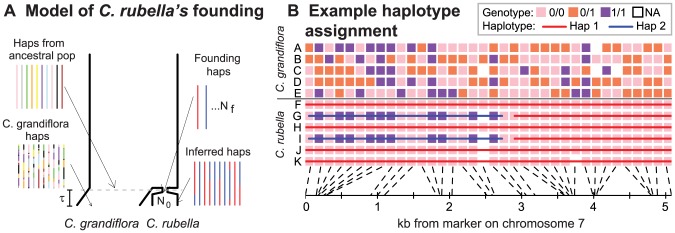
The founding of *C. rubella* and the identification of its founding haplotypes. *A)* A cartoon coalescent model of *C. rubella*'s origin. At time, 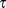, a population ancestral to *C. rubella* is formed by sampling 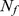 chromosomes (i.e. haplotypes, haps) from a large outcrossing population ancestral to both species, and this selfing population quickly recovers to size, 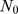. Because some of the 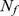
lineages are lost to drift, we can identify the founding haplotypes surviving to the present, which we color in red and blue. While recombination scrambles ancestral chromosomes in *C. grandiflora*, the low effective recombination rate in *C. rubella* ensures that large chunks of founding haplotypes remain intact. *B)* We aim to identify these founding haplotypes by using patterns of sequence variation (see text and *METHODS* for details of our algorithm). Here, we present an example of founding haplotype identification in a typical genomic region. To aid visualization, we label the major allele in *C. rubella* as ‘0’, and the allele that is rare or absent *C. rubella* as ‘1’, and only display genotypes at sites with common variants in *C. grandiflora*. In the left hand side of Figure 1B, there are clearly two distinct founding haplotypes on the basis of patterns of variation at sites polymorphic in both species. On the right hand side, all *C. rubella* individuals are identical at sites polymorphic in *C. grandiflora*, so we infer a single founding haplotype.

Figure 1 illustrates our approach to identifying *C. rubella*'s distinct founding haplotypes, a framework which will likely apply to many recently evolved selfing species. At a given locus, all extant individuals trace their ancestry to one of a small number of founding lineages (which, for brevity, we call ‘founding haplotypes’) that survive to the present (Figure 1A). These founding haplotypes should persist for long genetic map distances, given the recent origin of *C. rubella* and low effective recombination rate under selfing [22].

We define founding haplotypes as distinct *C. rubella* lineages that do not share a common ancestor until they are present in the population ancestral to *C. rubella* and *C. grandiflora*. A common way that this could occur is from the incomplete sorting of ancestral variation (Figure 1A). While a founding haplotype could, in principle, be introduced via introgression from *C. grandiflora*, the lack of evidence for introgression (above) suggests that this is rare. While we observe no evidence for recent introgression, we note that our inferences, with the exception of the coalescent modeling later, do not rely on assuming that introgression is rare.

Using the model in Figure 1A, we develop a non-parametric framework to robustly identify the genomic regions where two *C. rubella* individuals both have the same founding haplotype, versus two different ones (see Figure 1B for an example, and *METHODS* for more details). A pair of individuals must have different founding haplotypes in genomic regions where they differ at multiple sites that are polymorphic in both species (assuming no recurrent mutation). We therefore assign pairs of individuals to distinct founding haplotypes in genomic regions where they consistently differ at sites segregating in both species, and to the same founding haplotype where they are identical at such sites (see for example the left portion of Figure 1B). Also, in stretches of the genome where a number of sites are polymorphic in *C. grandiflora* but fixed in *C. rubella*, we assign all *C. rubella* individuals to the same founding haplotype (see for example the right portion of Figure 1B, see *METHODS*).

To ensure robust founding haplotype calls we identified ‘ambiguous’ genomic regions, where the assignments for different pairs of *C. rubella* individuals reveal conflicts (e.g. for three individuals, A, B, and C, A = B, B = C, A≠C, due to missing data, where = and≠refer to the same or different preliminary haplotype assignment, respectively). Because haplotype calling in such ‘ambiguous’ regions is problematic, we exclude them from our analysis (and return to discuss these genomic regions later). In the *METHODS* we describe these algorithms fully, with details of the number of SNPs and physical distances that we require to assign samples to the same or different founding haplotypes. In Text S1 we show that our results are robust to these cutoffs.

#### Patterns of pairwise founding haplotype sharing

**Figure 2:**
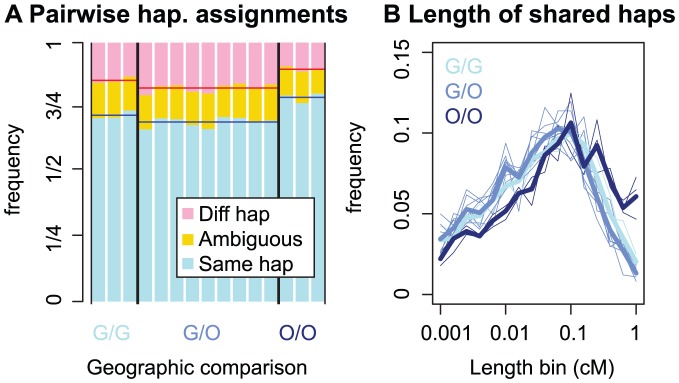
Patterns of founding haplotype sharing in *C. rubella*. *A)* The proportion of the genome for which two individuals have inherited the same or different founding haplotypes, or for which haplotype calls are ambiguous (see text for explanation). The geographic origin of the pair is denoted by G (Greek), or O (Out-of-Greece), e.g. a comparison between a Greek and Out-of-Greece pair is denoted by ‘G/O’. *B)* The length distribution of regions assigned to the same founding haplotype. Thin lines represent pairwise comparisons and thick lines represent mean values for this pairwise measure within a geographic class. In *Text S1*, we recreate this figure utilizing physical, rather than genetic distances, and find qualitatively similar patterns (Figure S2).

Figure 2A shows the proportion of the genetic map for which two *C. rubella* individuals are assigned to the same founding haplotype (on average 72%), distinct founding haplotypes (15%), or for which haplotype assignment is ambiguous (13%). Figure S2 shows similar results measured by proportion of the physical map, and Figure S3 shows the robustness of these results to haplotype assignment cutoffs. In total, pairs of individuals transition between the same to different founding haplotypes between 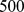 and 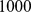 times, depending on the comparison. Therefore, the haplotype-based analyses, below reflect at least 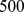, and likely many more, different coalescent events per pair of individuals.

As expected, assignment of pairs of samples to the same or different founding haplotype is consistent with patterns of pairwise sequence diversity reported above. Out-of-Greece pairs are assigned to the same founding haplotype more often than pairs from Greece, and comparisons between a Greek and Out-of-Greece plant have the lowest proportion of founding haplotype sharing. The same pattern is reflected in the length distribution of founding haplotype blocks (Figure 2B). This high level of founding haplotype sharing suggests that there has been extreme drift during or subsequent to the founding of *C. rubella*, particularly outside Greece.

#### Patterns of polymorphism within and between founding haplotypes

We next used these founding haplotype designations to partition patterns of polymorphism. We denote comparisons between individuals assigned to the same founding haplotype in a genomic region, averaged across all such regions genome-wide, by the phrase, ‘within founding haplotypes’. In turn, we denote comparisons between individuals assigned to different founding haplotypes, averaged across all such regions genome-wide, by the phrase, ‘among founding haplotypes’. As above, the subscripts N and S refer to synonymous and on synonymous sites, respectively. To provide empirical 95% confidence intervals for reported statistics, we resample regions of haplotype assignment with replacement.

#### Diversity within founding haplotypes is low, diversity among founding haplotypes is high

**Figure 3:**
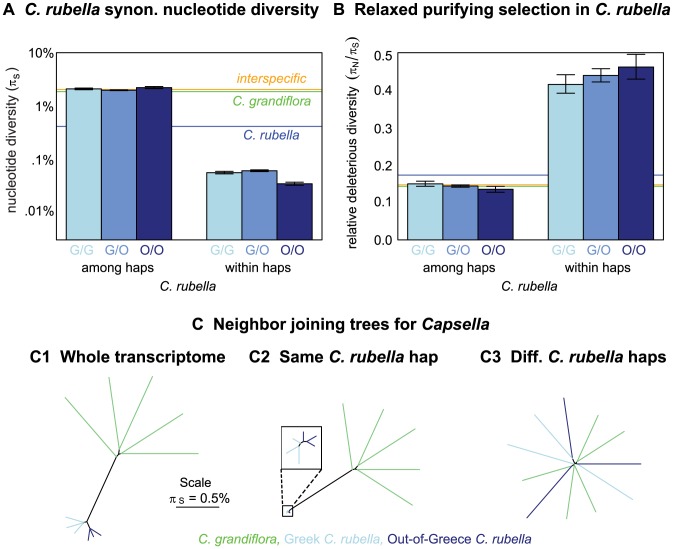
Variation within and among *C. rubella*'s founding haplotypes. *A)* Pairwise nucleotide diversity (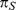) within and among *C. rubella*'s founding haplotypes at synonymous sites (see Table S3 for values). *B)* Ratio of nucleotide diversity at non-synonymous relative to synonymous sites (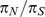) within and among *C. rubella*'s founding haplotypes. Error bars mark the upper and lower 2.5% tails and are generated by resampling blocks assigned to different (left hand side) or same (right hand side) founding haplotypes. In the top panel (A and B), orange, green, and blue horizontal lines are drawn for reference to interspecific comparisons, comparisons within *C. grandiflora*, and genome-wide *C. rubella* comparisons, respectively (taken from Table 1). *C)* Neighbor joining trees in *Capsella*, using all comparisons (*C.1*), comparisons within (*C.2*), or among (*C.3*) founding haplotypes to generate entries in the pairwise distance matrix for comparisons within *C. rubella*. All distances are generated from nucleotide diversity at synonymous sites.

For pairs of *C. rubella* samples, we estimated 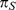 in genomic regions assigned to the same or different founding haplotypes. Regardless of the geographic origin of the *C. rubella* plants analyzed, 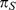 among haplotypes is similar to estimates of interspecific diversity (Figure 3A). This suggests that our inferred founding haplotypes correspond well to *C. rubella*'s founding lineages. By contrast, diversity within founding haplotypes is very low – approximately an order of magnitude lower than baseline diversity in this inbred species (Figure 3A). Additionally, the amount of variation within founding haplotypes depends on the geographic location of samples. As in genome-wide summaries, diversity within founding haplotypes is highest across geographic comparisons, lowest in Out-of-Greece pairs, and intermediate within Greece pairs (Figure 3A). All of these results are robust to cutoffs for founding haplotype assignment (Figure S5A). Since variation within founding haplotypes must have arisen since *C. rubella*'s founding, this paucity of variation could reflect either little time to accrue novel mutations, or a small effective population size limiting the extent of variation. Below, we show that the small effective population size explanation is a strong explanation of these data.

**Figure 4:**
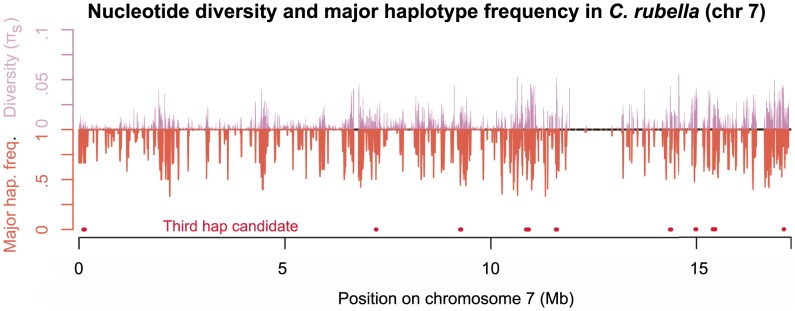
Diversity across chromosome seven in *C. rubella*. Mean pairwise synonymous diversity (purple, upward pointing lines) and major founding haplotype frequency (orange, downward pointing lines) across chromosome seven. Red points mark regions putatively containing more than two extant founding haplotypes. Values of 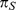 and major founding haplotype frequency are averaged across overlapping sliding windows (ten kb windows with a two kb slide), here only windows with data for 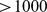 sites of pairwise comparisons are evaluated. See Figure S7, for plots of all chromosomes.

These results offer a straightforward interpretation of *C. rubella* diversity across the genome as a mosaic of relatively few founding haplotypes that have survived to the present day. Thus, we expect sequence diversity to vary as we transition between genomic regions with different numbers and frequencies of surviving founding haplotypes. Patterns of polymorphism are consistent with this view – there is a strongly negative relationship between the frequency of the most common founding haplotype and sequence diversity (Pearson correlation, 
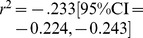
, see Figure 4 and Figure S7).

To further aid visualization of the structure of variation within and among founding haplotypes we present a set of neighbor joining trees constructed from pairwise distance matrices (Figure 3C). The tree constructed from the entire transcriptome (Figure 3C.1) shows little genetic diversity within *C. rubella*, the distinctness of *C. rubella* from *C. grandiflora*, and the clustering of Out-of-Greece *C. rubella* samples. In contrast, Figure 3C.2 reveals diversity within founding haplotypes is completely dwarfed by diversity within *C. grandiflora* and interspecific divergence; however, by zooming in on the *C. rubella* branch of this tree we recover the clustering of Out-of-Greece samples (top left of Figure 3C.2). Comparisons among founding haplotypes reveal a starlike structure for all sequences (Figure 3C.3). Because *C. rubella* samples that have different founding haplotypes do not cluster with one another, this suggests that *C. rubella*'s founders were close to a random selection of ancestral variation, rather than a distinct *C. rubella* sub-population, and that there has been little allele frequency divergence genome-wide in *C. grandiflora* since the founding of *C. rubella*.

#### Putatively deleterious variation is overrepresented within founding haplotypes

The ratio of non-synonymous to synonymous variation among *C. rubella*'s founding haplotypes is low, resembling that found in *C. grandiflora*. By contrast, much of the diversity within founding haplotypes is nonsynonymous (nearly one-third) (Figure 3B), a result that is robust to founding haplotype calling cutoffs (Figure S5B). Since the excess nonsynonymous variation in *C. rubella* is segregating within haplotypes, and therefore novel, elevated nonsynonymous diversity in this species suggests a relaxation in the efficacy of purifying selection following the transition to selfing [23]. This elevated 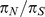 within compared to among founding haplotypes, is also reflected in patterns of variation at polymorphic sites private to a species sample. That is, the ratio of nonsynonymous to synonymous polymorphisms unique to our *C. rubella* sample is 3.5 fold higher than this ratio in polymorphisms unique to our *C. grandiflora* sample. Overall this shows that polymorphisms that have arisen since the founding event within *C. rubella* are strongly enriched for non-synonymous, likely deleterious, variants.

#### The frequency of different founding haplotypes in *C. rubella*

Building on pairwise founding haplotype assignments, we identified distinct founding haplotypes across the *C. rubella* genome. This higher-order haplotype assignment provides information about both the frequency spectrum of founding haplotypes, and the allele frequency spectrum within founding haplotypes.

To construct the set of founding haplotypes in a genomic region, we simultaneously evaluate all patterns of pairwise founding haplotype assignment in this region (see *METHODS* for a complete description of the algorithm). For example, in the left hand side of Figure 1B, all pairwise comparisons between individuals F, H, J, and K show them to be identical at sites polymorphic in both species, and so they are assigned to haplotype 1 (indicated by red lines). Similarly, individuals G and I are assigned to the same founding haplotype (haplotype 2, blue lines), which is distinct in pairwise comparisons from founding haplotype 1. On the right hand side of this figure all *C. rubella* samples are identical for a stretch of sites polymorphic in *C. grandiflora*, and so are assigned to the same founding haplotype.

#### A summary of founding haplotype assignment

Using these assignments, we find that for 57% of the genome, *all C. rubella* individuals in our sample have inherited the same founding haplotype, for 19% of the genome, all individuals can be unambiguously assigned to one of two haplotypes, and for 25% of the genome at least one individual could not be unambiguously assigned to a founding haplotype. The fact that so much of the *C. rubella* genome contains so little diversity in founding haplotypes suggests that either very few individuals founded *C. rubella*, or that nearly all of the diversity present in a large founding population has been lost by subsequent drift and selection. Below we use the frequency spectrum within founding haplotypes and coalescent modeling to distinguish between these possibilities.

#### Regions with more than two founding haplotypes

Overall 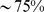 the genomes of our *C. rubella* samples can be unambiguously assigned to 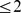 founding haplotypes. The remaining quarter of the genome is split between genomic regions with more than two founding haplotypes, ambiguous haplotype assignment and/or transitions between haplotypes for at least one sample (see Table S2 for the sensitivity of these results to haplotype calling cutoffs). Convincing evidence for even a single genomic region containing more than two founding haplotypes would rule out the hypothesis that the ancestry of *C. rubella* can be traced to a single founder with no subsequent introgression [17]. However, there are numerous alternative reasons why a small portion of the *C. rubella* genome may appear to contain more than two founding haplotypes. These explanations include the misalignment of paralogous regions as well as incorrect founding haplotype assignments caused by multiple historical recombination events. We therefore carefully investigate the possibility that some genomic regions contain more than two founding haplotypes.

We identified genomic regions likely containing more than two founding haplotypes by a sliding window analysis moving across the genome of all trios of our six *C. rubella* samples. In windows of 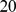 sites with more than one copy of the minor allele in *C. grandiflora*, moving one such SNP at a time, we noted candidate regions where each member of the trio differs from the others at one or more of these SNPs. We pruned this list of candidates in two ways. We included only windows where each member of the trio is differentiated by 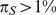 in the candidate region, a level much higher than that within founding hapotypes and within the range of diversity in *C. grandiflora*, to ensure that the windows likely include 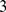 distinct founding haplotypes. To minimize the chance that such high diversity regions represent misassembly, we required that at least one member of the trio is similar to another sample (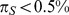) in that genomic region.

We identified 172 genomic regions likely to harbor more than two founding haplotypes, and we present nine exemplary regions in Figure S10. In total, such regions make up approximately 2% of the genome. These regions are generally quite short (53 are 10 kb or less, 132 are less than 20 kb, and all are shorter than 70 kb). The length distribution of genomic regions with 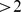 haplotypes likely reflects recombination since the origin of *C. rubella*, and suggests that these additional founding haplotypes have probably not been recently introduced by introgression. Given their small size and our stringent criteria, we likely have underestimated the fraction of the genome with 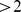 founding haplotypes.

#### No excess of high frequency derived alleles within *C. rubella* founding haplotypes

**Figure 5:**
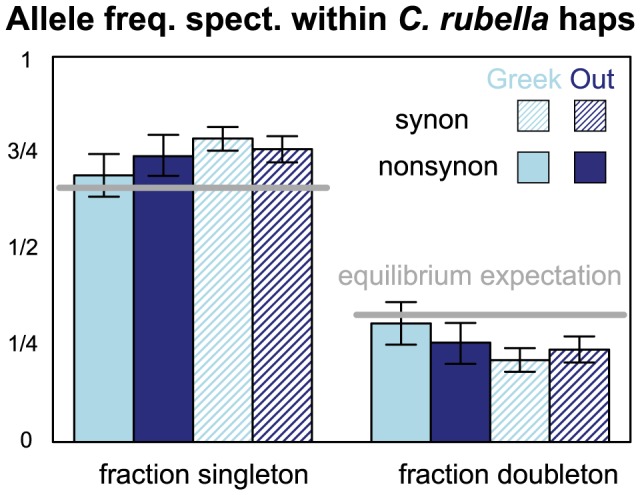
The allele frequency spectrum within *C. rubella*'s founding haplotypes. The proportion of polymorphic derived alleles within a founding haplotype observed as singletons or doubletons, split by geography and synonymy. Light and dark blue represent comparisons within Greek and Out-of-Greece samples, respectively. Filled and hatched bars represent synonymous and non-synonymous sites, respectively. Error bars represent the upper and lower 2.5% tails of the allele frequency spectrum when founding haplotypes are resampled with replacement. Grey lines represent expectations of a model for neutral mutations at mutation-drift equilibrium.

We make use of the allele frequency spectrum within founding haplotypes to distinguish between two alternative models of *C. rubella*'s origin – an extreme but short-lived bottleneck at its origin or a long-term reduction in population size. Within founding haplotypes, the frequency spectrum in Greece resembles the expectation under a constant population size model, and there is only a slight excess of rare derived alleles outside of Greece (Figure 5), a result robust to the choice of cutoffs for the labeling of founding haplotypes (Figure S4). Since diversity within founding haplotypes is close to its expectation under drift-mutation equilibrium, the low level of variation within founding haplotypes in Greece reflects a small long-term effective population size, rather than solely the effect of a dramatic bottleneck at the founding of C rubella (we quantify this statement shortly through coalescent modeling). The slight excess of singletons within haplotypes outside of Greece is consistent with an out-of-Greek expansion; however, given the broad geographic sampling we cannot exclude the confounding effect of population structure [24].

We also used the allele frequency spectrum to test alternative explanations of the excess of nonsynonymous variation within founding haplotypes. Specifically, this elevated 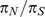 could represent a relaxed efficacy of purifying selection in *C. rubella*, or may reflect a departure from demographic equilibrium (whereby the excess of non-synonymous variants is due to the fact that many of the variants in *C. rubella* are young and hence at low frequency). However, the similarity of the allele frequency spectrum at synonymous and non-synonymous sites within founding haplotypes (Figure 5) argues against a demographic explanation for elevated 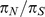 and suggests a weakening efficacy of purifying selection in *C. rubella*, presumably cause by its reduced effective population size.

#### Inferring the number of founders and the timing of speciation

So far, we have examined patterns of diversity in *C. rubella* with little reliance on specific models or assumptions. To complement these analyses, we build a coalescent-based framework to infer the parameters of a simple demographic model of *C. rubella*'s history from the results above. To facilitate this inference we introduce a few assumptions. The most restrictive of these is that introgression between *C. rubella* and *C. grandiflora* has been negligible. While we cannot rule out the possibility of infrequent and/or very old introgression events, the similarity in divergence between *C. grandiflora* and both Greek (sympatric) and Out-of-Greece (allopatric) *C. rubella* populations, and the positive 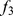 statistic (*Text S1*) argue against recent common introgression. Additionally, conversion of synonymous site diversity measures into a time-scale of years requires assumptions about the mutation rate, variation in this rate, and life-history. Following previous work on *Capsella* [16], [17], we assume an average neutral mutation rate (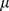) of 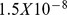 per base per generation [25] in both species and an annual life history, so that a neutral position in *C. rubella* experiences 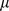 mutations per a year. To change these rate assumptions, divergence times can be linearly rescaled by alternative estimates of 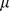 and/or life history descriptions. For example, to use a more recent estimate of 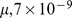 [26], we can simply multiply our estimates, below, by roughly a factor of two.

As a first estimate of the split date between *C. rubella* and *C. grandiflora*, we use levels of diversity within and between species to estimate a divergence time (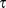, following [27]). In addition to assuming no introgression, this model also assumes that the expected pairwise coalescent time in *C. grandiflora* is the same today as it was in the population ancestral to *C. grandiflora* and *C. rubella*. Under these assumptions, divergence at synonymous sites should be given by 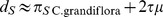. Solving for 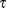 and substituting our estimates of 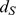 and 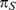 within *C. grandiflora*, we estimate a split time of 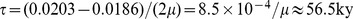.

#### Demographic model

The estimate above provides an approximate divergence date but no additional details about the founding of *C. rubella*. We aim to build a model that captures the major demographic events in *C. rubella*'s history and makes use of the founding haplotype approach introduced in this manuscript. Throughout, we limit this analysis to four exchangeable samples (three from Greece and one from Out-of-Greece), so that our inference is not misled by population structure [24]. Unlike the divergence estimate above, this model is robust to both introgression from *C. rubella* into *C. grandiflora*, and to changes in *C. grandiflora*'s effective population size, but assumes no introgression from *C. grandiflora* into *C. rubella* in the last 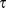 generations.

Inspired by previous methods that aim to infer the number of founding chromosomes from patterns of genetic variation [28], [29], we use coalescent modeling to jointly estimate the number of founding chromosomes and the time of *C. rubella*'s founding. We use the model (depicted in Figure 1A) where *C. rubella* was founded 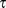 generations ago by a founding population of 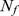 (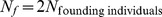) founding chromosomes, which instantly grew to its current effective population size of 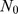 chromosomes. We infer the parameter, 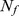, and the compound parameter of the population-scaled founding time 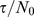 in a composite likelihood framework (see *METHODS* for full details). To do so, we generate expected values of the allele frequency spectrum within founding haplotypes and the fraction of genomic windows where all samples inherited the same founding haplotype by simulating a coalescent model across a grid of 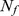 and 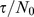. We then compute the composite likelihood of these aspects of our data across a grid of 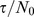 and 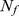, and resolve the compound parameter, 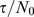, by including information contained in diversity within founding haplotypes. In *Text S1* we show that our inferences are robust to the choice of cutoffs for the labeling of founding haplotypes (Figure S6).

**Figure 6:**
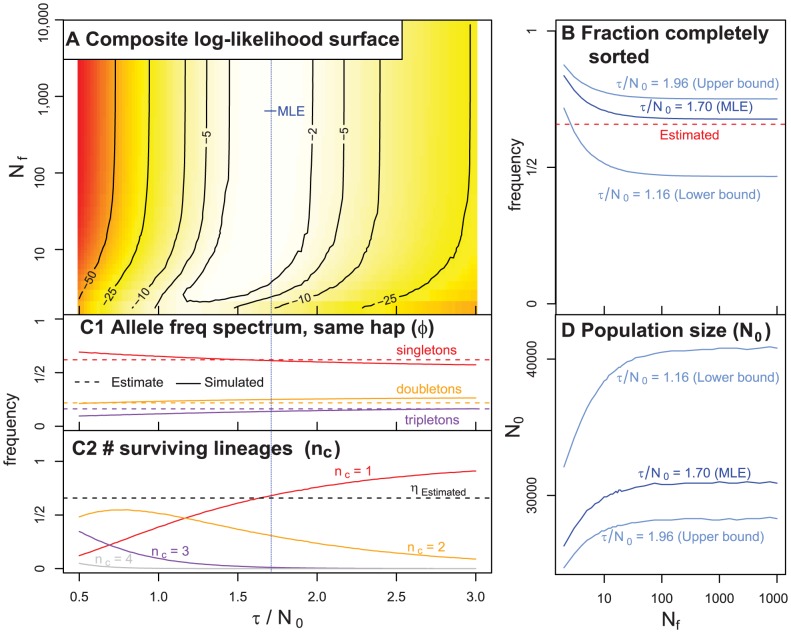
A summary of our coalescent model of the history of *C. rubella.* * A)* The relative composite log-likelihood surface as function of 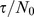 and 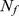. *B)* The probability that all individuals coalesce to the same founding haplotype (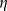) as a function of 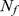 and three estimates of values 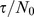 (the MLE, lower and upper confidence intervals). The dotted red line indicates the value of (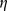) directly estimated from the data. *C)* A summary of simulation results (assuming 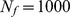). *C1)* The frequency of singletons, doubletons, and tripletons observed in simulation (full lines), and estimated from our data (dashed lines) conditional on all four samples deriving from the same founding haplotype. *C2)* The frequency of one, two, three or four lineages surviving to the founding event. When 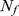 is large, 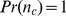 is the probability that all samples coalesce to the same founding haplotype. The dotted black line portrays the estimated frequency of all four samples residing on one founding haplotype 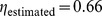. *D)* The estimated current effective number of chromosomes in *C. rubella* (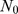) as a function of the number of founding chromosomes (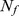). We plot this for three different values of 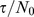 (the MLE, as well as the lower and upper confidence intervals). These results are robust to haplotype labeling criteria in Figure 6 (see *Text S1, Figure S6*).

Our likelihood surface with respect to *C. rubella*'s population-scaled founding time (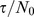) shows a strong peak at a relatively large value of 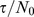 (MLE = 1.7, with two log likelihoods confidence interval of 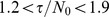, Figure 6A). This reflects the frequency with which all individuals inherit the same founding haplotype (Figure 6B), the slight excess of singletons within founding haplotypes, and the preservation of alternative founding haplotypes in *C. rubella* (Figure 6C). Given this estimated range of 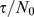, we resolve this compound parameter by using our estimate of diversity within founding haplotypes (see *METHODS*). Doing this, we infer the current effective number of chromosomes, 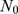 (Figure 6D), to lie between 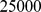 and 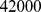, and a split time, 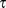, between 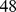 and 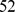 kya. This range is reasonably consistent with our estimated split time of 56 kya obtained using a relatively independent source of information (see above). Our likelihood surface shows a long ridge in parameter space with respect to the number of founding chromosomes (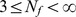 two log-likelihood confidence interval). Therefore, while our data are consistent with few to many founding individuals, a single founder is particularly unlikely.

## Discussion

We present a novel framework to interpret patterns of sequence diversity in recently founded populations by viewing the genome as stretches of ancestry inherited from distinct founding chromosomes. We exploit this view to provide a detailed characterization of the evolutionary transition from outcrossing to selfing in *C. rubella*. In principle, our conceptual approach is applicable to any founding event recent enough to preserve a reasonable portion of polymorphism present in the founders, regardless of mating system. The application to *Capsella* was aided by the fact that few founding lineages contribute ancestry to our *C. rubella* sample, and that levels of linkage disequilibrium differ so starkly between *C. rubella* and *C. grandiflora*, making identification of the founding haplotypes relatively easy. As these criteria are met by many recently founded selfing species and populations (e.g within *Leavenworthia*, *Mimulus spp.*, *Arabidopsis lyrata*, and *Clarkia xantiana* [30]–[34]), including a number of commercially important species (e.g. indica rice and soybean [35], [36]), our framework should be of broad use as population genomic resources continue to be developed in these systems [37]–[39].

Our approach provides a new way of thinking about patterns of nucleotide diversity across the genomes of recently derived selfers. Moving across two phased genomes, we transition between regions in which our samples coalesce at or since the origin of selfing, and regions in which samples do not coalesce until they join the ancestral outcrossing population. Critically, we can use polymorphism present in a proxy for the outcrossing progenitor population (*C. grandiflora*) to assess if two individuals have inherited the same or different founding haplotypes, since individuals that differ at ancestrally segregating sites almost certainly inherited different founding haplotypes.

### Concerns about samples sizes

While we have sequence data from only six *C. rubella* samples (and often make use of three to four genomes to control for population structure), these transcriptomic data provide information about hundreds to thousands of genealogical histories as we move along the genome. Therefore the small number of sequenced individuals provides plentiful information about population history. A recent demonstration of this principle is the development of coalescent methods to infer population history from a single individual's genome [40].

In particular, our findings about the small number of founding haplotypes are likely generalizable to the population, since much of the common diversity (i.e. that contained in the deep parts of the genealogy) in large samples is expected to be found in small samples [41]. This view is supported by the consistency of our findings and those of Guo et al. [17], who usually found one or two distinct haplotypes at each of 17 loci in a survey 25 *C. rubella* individuals.

While it is likely that our analyses, based on small sample sizes, have captured many aspects of the founding of *C. rubella*, larger samples will provide a fuller view of recent events. For example, additional genome-wide samples would provide access to lower frequency variants (i.e. more novel mutations), providing information about more recent population growth [42], [43], and finer resolution of population structure. Additionally, sequence data from more individuals would provide a finer resolution to the frequency spectrum of ancestral polymorphisms, and would help clearly identify genomic regions with more than two founding haplotypes. Therefore, additional samples could facilitate a more refined view of *C. rubella*'s initial founding, and could potentially narrow the confidence intervals on our estimates of founding time, population growth rates, and population size.

### A new view into the history of *Capsella rubella*

Our haplotype-based approach provides a rough characterization of the history of the selfing species, *C. rubella*. We note that since we have sequence data for only a handful of samples, we cannot provide fine resolution of recent demographic events in the history of the species. Assuming a mutation rate of 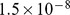 [25], we infer that approximately 50 kya, a *C. grandiflora*-like ancestral population of unknown size became largely selfing and gave rise to *C. rubella*. Much of the ancestral diversity present in the founding population has since been lost due to subsequent drift and selection. In fact, two *C. rubella* individuals inherit different founding haplotypes for on average only 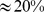 of their genome. Despite this, the diversity maintained from the founding population makes up roughly 90% of extant pairwise sequence diversity in *C. rubella*, since little diversity has arisen since its founding. We now turn to discuss some of the specifics of the founding and subsequent history of *C. rubella*.

### No obvious signal of an extreme bottleneck

High levels of autozygosity associated with selfing can reduce the effective population size of a selfing species to less than 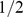 of the same outcrossing population [44], [45]. Therefore, all else being equal, neutral diversity in selfing taxa should be no less than half of that observed in their outcrossing relatives. As selfing species often exhibit a greater than two-fold reduction in diversity, severe founding bottlenecks are often presented to explain this discordance (e.g. in *C. rubella* [16], [17]); however, alternative explanations, including the greater reach of linked selection in selfing populations have also been proposed [46]–[50] (see below). Such founding bottlenecks are seen as evidence supporting the idea that selfing species are often founded by a small number of individuals, consistent with reproductive assurance favoring the evolution of selfing [8], [51].

The very low levels of diversity within *C. rubella* seemed initially to be consistent with this view [17]. Indeed, we find that for a given genomic region, few founding lineages drawn from a *C. grandiflora* - like population contributed ancestry to present day *C. rubella*. However, this reduction in *C. rubella*'s diversity relative to *C. grandiflora*, and the observation of only one or two extant founding haplotypes in most genomic regions (as previously observed [17]) is due to an extreme loss of variation subsequent to the founding of *C. rubella*, and does not necessarily imply an extreme founding bottleneck. This loss of variation is likely due to an extreme reduction in *C. rubella*'s effective population size, the potential causes of which we discuss shortly.

The high level of drift due to this small 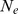 confounds our ability to estimate the actual number of founding chromosomes, because the genetic contribution of founders has been lost (see [28], [29] further discussion). We therefore caution that low long-term effective population sizes in selfing plants may erode historical signals of their founding. Our likelihood based inference as well as our evidence for more than two founding haplotypes in some genomic regions argues against the hypothesis that *C. rubella* was founded by a single plant with no subsequent secondary contact from *C. grandiflora* ; however, we lack sufficient information to pinpoint the founding population size.

The patterns of diversity that have arisen since *C. rubella*'s founding are consistent with a population at approximately mutation-drift equilibrium with a small long-term effective population size. In fact, we estimate a twenty-fold reduction in *C. rubella's* effective number of chromosomes from the ≈600,000 in *C. grandiflora*. Although the causes of this reduced effective population size are unclear, numerous forces, including frequent oscillations in population size, linked selection, etc. may be responsible [49], [52]–[54], and future work on the determinants of 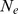 in selfing species will clarify this issue.

This small effective population size has led to a rapid loss of diversity since *C. rubella*'s founding. While some genomic regions maintain multiple extant founding lineages and high levels of pairwise sequence diversity, if this small size persists *C. rubella* will quickly lose much of its genetic variation. For example, currently two individuals inherit the same founding haplotype for approximately 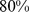 of the genome, resulting in a profound lack of diversity. At the current rate, it will take only another 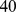
ky for 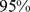 of the genome of two individuals to be homozygous for all ancestral variation. This would reduce genome-wide 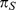 in *C. rubella* to 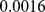, severely limiting the pool of standing variation available for a response to selection. Perhaps it is this low diversity that limits the adaptive evolution [55] of selfing species and contributes to their eventual demise [12]–[14].

### Relaxed efficiency of purifying selection in *C. rubella*

Viewing *C. rubella*'s founding haplotypes as a random draw from an ancestral *C. grandiflora* -like population, we expect (and indeed observe – Figure 3A) comparable 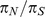 values among *C. rubella*'s founding haplotypes and within *C. grandiflora*. Therefore, the founding of *C. rubella* did not itself facilitate the accumulation of deleterious mutations, contrary to expectations from a model where an extreme reduction in 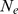 at the species founding allowed deleterious mutations to markedly and suddenly increase in frequency. Rather, the long-term reduction in *C. rubella*'s effective population size lessened the efficacy of purifying selection, as is reflected by the threefold increase in 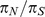 within founding haplotypes as compared to between species, founding haplotypes, or within *C. grandiflora*. Our view of the origin of deleterious mutations in *C. rubella* can reconcile two seemingly contradictory observations – that 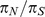 within selfing species is large but 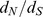 between selfers and close relatives is unremarkable (e.g. [56]). The unremarkable 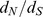 between selfers and their relatives reflects the fact that since selfing species are generally young, an overwhelming portion of their divergence from outcrossing relatives is simply the sorting of ancestral variation. By contrast, the high 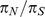 observed within selfing species reflects the rapid homogenization of most initial variation in selfing taxa, and the weakening of purifying selection against novel non-synonymous mutations, which can make up a substantial portion of intraspecific variation while hardly contributing to interspecific divergence.

### Future prospects

With our haplotype-based approach, we provide a reasonable sketch of *C. rubella*'s history. However, numerous questions remain. Future work on the population genomics of selfing will identify the cause(s) of the reduced effective population size often observed in selfing populations, highlight the role of rare introgression in the evolution of selfing, identify recent fluctuations in the size of selfing populations, and inform the geographic spread of selfing lineages. While full sequence data from more individuals will further illuminate these issues, our result highlight the vast information about species' origin present in population genomic data. Future analyses like the one presented here will help further refine our genomic understanding of the evolutionary transition to selfing.

## Materials and Methods

### Sequencing, alignment, and sequence quality

We utilized genotype data from 38 bp paired-end sequencing of RNA extracted from flower bud tissue of 11 samples (6 *C. rubella* and 5 *C. grandiflora*). These reads were then mapped to the *C. rubella* reference genome using Tophat [57] (v.1.3.0) as described previously [15] (using an inner distance between reads (-r) of 100, and minimum and maximum intron length of 40 and 1000 respectively). To call SNPs from the RNA data, we utilized the GATK pipeline on the BAM files [58], [59]. We instituted straightforward QC steps, and treated all genotypes with coverage less than 10×, quality scores (from the GATK pipeline) less than 30, and/or heterozygous sites in putatively autozygous regions as missing data.

To validate our calls we compared our genotype data to 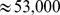 sites of Sanger sequencing and found very little discordance (see *Text S1*, Table S1), and nearly identical diversity measures (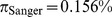, 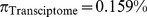, for 72,066 and 71,645 pairwise comparisons between base pairs, respectively). We analyzed all loci where individual genotypes passed quality control standards allowing us to utilize sites with partially missing data, a slight departure from the initial presentation of this data set [15], which only examined sites where all individuals passed QC. We focus on divergence and diversity at fourfold degenerate (i.e. synonymous) and zero fold degenerate (i.e. nonsynonymous) sites to view patterns of neutral and putatively deleterious variation within and among species.

### Identifying allozygous regions through patterns of heterozygosity

Given the high selfing rate in *C. rubella*, [18] the genome of a *C. rubella* individual is expected to be mostly autozygous. However, some allozygous regions are expected in field-collected samples of a species with a non-zero outcrossing rate. Indeed, we observe heterozygous sites in our *C. rubella* samples. Such sites could be caused by genotyping and/or alignment error, *de novo* mutations, or residual heterozygosity retained since a lineage's most recent outcrossing event (i.e. heterozygosity in allozygous regions). Since allozygous loci will be clustered in the genome due to the limited number of generations for recombination since the most recent outcrossed ancestor, while sequencing errors will be distributed relatively uniformly across the genome, we utilize the distribution of heterozygous sites across the genome to separate allozygous regions from sequencing error in *C. rubella*. More specifically, we identify allozygous regions by examining the local density of heterozygous sites. These regions are generally quite obvious (Figure S9A–F), so we visually identified the beginning and ends of these allozygous stretches of the genome within an individual.

We treat these allozygous regions of an individual's genome as missing data. Reassuringly, the average heterozygosity within an individual in these allozygous regions (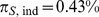) closely matches the pairwise diversity between individuals (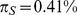 see Figure S7). This gives us confidence that by treating these allozygous regions as missing data for an individual we are not biasing ourselves away from interesting genomic regions of high diversity. By contrast, nearly all heterozygous sites in putatively autozygous regions should be artifacts (e.g. sequencing error, misalignment, etc.), and very few should represent *de novo* mutations that have arisen since the region was last made homozygous by descent due to inbreeding. In inferred autozygous regions on average 0.13% of synonymous sites are heterozygous. This error rate varies across individuals (see *Text S1*, Figure S8), corresponding to sequencing lane. We treat these heterozygous sites in allozygous regions as missing data in our population genomic analyses.

### Identifying founding haplotypes

Since *C. rubella* and *C. grandiflora* have recently split, much variation within each species is incompletely sorted variation inherited from a population ancestral to both. In *C. rubella*, this ancestry can persist for long physical distances, due to its recent founding and low effective recombination rate. We can therefore hope to infer the haplotypes that contributed to the founding of extant *C. rubella* diversity. In doing so, we do not attempt to assign founding haplotypes in regions between informative data, therefore minimizing our uncertainty in founding haplotype assignment.

One of the strengths of this approach is that even ancestrally polymorphic alleles that are missing from our small sample of extant *C. grandiflora* diversity, but by chance are found in our *C. rubella* sample, are likely to be correctly identified as differences among founding haplotypes, rather than contributing to difference within founding haplotypes. This follows from the fact that such sites will often be flanked by jointly polymorphic sites that were common in the ancestral population, allowing us to correctly assign the status of founding haplotype sharing.

*Preliminary haplotype assignment*: In some genomic regions, all of our samples will carry the same founding haplotype. Thus, we assign all *C. rubella* samples to the same founding haplotype in long regions (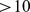 kb and 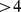 polymorphisms in *C. grandiflora*) where all *C. rubella* samples (with non-missing data) are identical at positions polymorphic in *C. grandiflora*.

We next focus on pairwise comparisons in regions where polymorphisms are jointly segregating, since such variation likely represents incompletely sorted ancestral variation. In regions of the genome where a pair of *C. rubella* individuals have inherited the same founding haplotype, they must have identical alleles at ancestrally polymorphic sites. We labeled all sites polymorphic in both species as a ‘same site’ if both individuals were homozygous for the same allele, and as a ‘different site’ if both individuals were homozygous for different alleles. We labeled sites as missing data if at least one of the pair did not pass QC at this site. We identified runs of haplotype sharing between two samples beginning with a ‘same site’ and ending at the last ‘same site’ before a ‘different site,’ ignoring sites with missing data. When these runs of ‘same’ sites extended more than 1.5 kb and consisted of at least 4 jointly polymorphic sites, we preliminarily assigned these individuals to the same founding haplotype.

In regions with ancestry from exactly two founding haplotypes (e.g. the left hand side of Figure1B), alternative founding haplotypes must differ at sites polymorphic in both species – that is, with two extant founding haplotypes, differences at jointly polymorphic sites are necessary and sufficient for assigning individuals to alternate founding haplotypes. In regions with more than two extant founding haplotypes, differences at jointly segregating sites are sufficient but not necessary for assigning individuals to alternate founding haplotypes, because two distinct founding haplotypes could be identical at the same jointly polymorphic allele. We explore alternative founding haplotype labeling rules in *Text S1*, and show that our results hold under most reasonable criteria.

*Higher order haplotype assignment*: Building on pairwise founding haplotype assignments, we aim to identify alternative founding haplotypes across the *C. rubella* genome. To do so, we broke the genome into windows of differing sizes corresponding to points in which runs of pairwise (same vs different) founding haplotype assignment begin and end across individuals. We then assigned individuals to founding haplotypes in each window as follows:

1. We did not attempt to infer the founding haplotype of an individual in a region where it was allozygous.
2. In invariant regions, we assigned all individuals to the same founding haplotype.
3. In all other regions, we assigned individuals with ‘same’ and ‘different’ founding haplotype assignments onto alternative founding haplotypes by constructing networks of haplotype sharing. To do this,
  - We began with the first individual (this choice does not affect the algorithm, see below) and found which (if any) others where on the same founding haplotype by the above criteria, and labeled all individuals as ‘founding haplotype one’.
  - We continued this process until no individuals are the same as founding haplotype one.
  - We then chose the first individual not assigned to founding haplotype one, and place it on founding haplotype two, finding the other individuals inferred to have inherited this founding haplotype as described above.
  - We continued this scheme, introducing additional founding haplotypes as necessary (i.e. repeating step 3), until all of these individuals where assigned to a founding haplotype.
4. Occasionally, we could not assign an individual to a founding haplotype in a region, and so we labeled this individual as ‘ambiguous’. This could occur for two reasons. The first is that due to missing data, there was discordance in our founding haplotype assignment, e.g. individual 1 was assigned to the same founding haplotype as individual 2 and 3, but individuals 2 and 3 were assigned to different founding haplotypes. To be conservative in such cases we labeled all three (or more) individuals as ‘ambiguous’ this both minimizes uncertainty and ensures that how we assign individuals in our algorithm does not influence our results. The second reason for an individual to be assigned an ‘ambiguous’ label is because pairwise assignments began and ended at the first and last different (or same) ancestrally polymorphic site, in some regions an individual was not assigned to the same or different founding haplotypes as any other samples. These regions could represent an individual switching rapidly between founding haplotypes due to historical recombination events, or a third founding haplotype present only once in our sample.

At the conclusion of this algorithm every individual was assigned to a founding haplotype (or labelled as ambiguous) for every genomic window where an individual was autozygous. We do not use these ambiguous regions when comparing within or among founding haplotypes, and we examine the possibility of regions with more than two founding haplotypes in the main text.

### Constructing neighbor joining trees

We used the *nj* function in the R [60] package ape [61] to construct neighbor-joining trees (presented in Figure 3C) from distance matrices containing subsets of our SNP data set at synonymous sites. For the entire transcriptome (Figure 3C1) we constructed the distance matrix where each off-diagonal element was the fraction of pairwise sequence differences between the pair of individuals (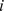 and 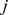) at synonymous sites, 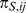 where 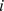 and 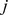 refer to rows and columns of the distance matrix. For the tree constructed within *C. rubella*'s founding haplotypes (Figure 3C2), we calculated the fraction of pairwise sequence differences between the pair of *C. rubella* individuals (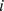 and 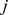) where we inferred 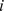 and 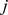 to have inherited the same founding haplotype. For the tree constructed among *C. rubella*'s founding haplotypes (Figure 3C3), we calculated the fraction of pairwise sequence differences between the pair of *C. rubella* individuals (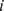 and 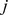) where we inferred 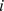 and 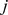 to have inherited different founding haplotypes. In both cases, entries in the distance matrix between pairs of *C. grandiflora* and *C. rubella*, and within *C. grandiflora* pairs where constructed by using all synonymous sites. We note that numerous recombination events clearly occurred during the history of these samples, and we therefore caution against interpreting this neighbor joining tree as a phylogenetic statement.

### Demographic inference

To infer the history of *C. rubella*, we simulated a coalescent model where at time 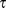, 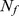 chromosomes founded a population that instantaneously grew to 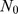 effective chromosomes (Figure 1A). To avoid potential confusion with the definition of the effective population size in selfers (see [62] for recent discussion), we directly used the effective number of chromosomes, 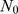, as our coalescent units, so that the rate of coalescence of a pair of lineages equaled 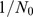. We note that our inference of the number of founding chromosomes was inspired by two recent papers [28], [29] that addressed this question using small numbers of micro-satellite and PCR amplified loci, respectively.

To infer the demographic parameters of interest (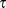, 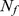, and 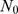), we made use of the frequency with which all samples are assigned to the same founding haplotype, 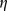, and the allele frequency spectrum in these regions, 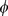. In our four exchangeable individuals (three Greek and one Out-of-Greece), 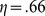, and 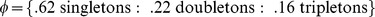. We aimed to estimate the composite likelihood of our data given our parameters, 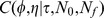, via coalescent simulation. As this likelihood depends on only 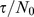 – the coalescent-scaled founding time, and not on 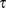 and 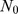 separately, we estimated the likelihood surface as a function of this compound parameter 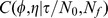. We then resolved these two parameters by considering nucleotide diversity within founding haplotypes (below).

For inference, we use a composite likelihood framework. Composite likelihoods approximate the full likelihood of the data as the product of the likelihoods of a set of correlated observations – ignoring their dependance. This facilitates inference in cases where obtaining the full likelihood is computationally prohibitive (see [42], [63], [64] for earlier population genetic applications). In making this approximation, composite likelihoods make the likelihood surface overly peaked, but do not produced a bias in the maximum likelihood estimate (MLE) [65], [66].

#### Coalescent simulations

We found 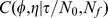 by generating expectations 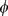 and 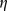 from 10,000 coalescent replicates across each cell in a fine-grained grid of 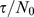 and 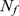. Specifically, we simulated the coalescent genealogy of four lineages in a population with 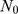 effective chromosomes, back to time 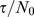. For a given simulation, our sample of four had coalesced to 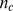 lineages (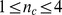) at time 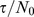. With probability, 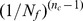, all 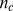 lineages coalesced to the same founding haplotype, at time 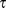, and with probability 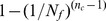 we expected more than one extant founding haplotype. For each simulation, we kept track of the proportion of simulations where all samples coalesced to the same founding haplotype (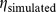), and a vector of the time with 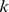 lineages, 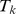 (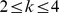).

*Likelihood of the allele frequency spectrum,* 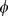: We used this distribution of coalescence times to calculate the expected allele frequency spectrum within a founding haplotype, 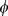, by computing the expected number of sites with 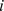 copies of a derived allele, 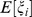, from [67]

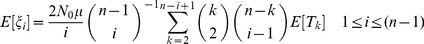
(1)Where 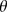 is the population mutation rate. We then converted 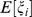 into the expected proportion of polymorphic sites observed 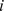 times in a sample of size 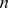, 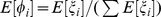, i.e. the expected frequency spectrum conditional on all four samples inheriting the same founding haplotype. Since 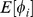 is independent of 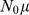 this value allowed us to disentangle 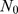 and 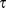, below. The probability of an allele frequency spectrum across many unlinked sites is multinomial with probabilities given by 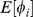 and the number of observations (i.e. the number of polymorphic sites within our four samples in regions where we inferred all to have inherited the same founding chromosome), which we used to estimate the composite likelihood of 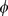 given the parameters, 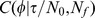.

*Likelihood of the proportion of the genome derived from a single founding haplotype,* 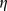: The probability that all samples coalesce to the same founding haplotype is binomial with probability 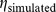, which we used to estimate the likelihood of 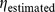 given the model. A difficulty with estimating the likelihood of 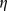 is that there is no natural observable unit for a founding haplotype to take a product of likelihoods over. We took a conservative solution to this challenge – since most regions where individuals share a founding haplotype are shorter than 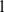
cM, and since our map covered 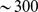 *cM*, we conservatively assumed that we observed 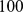 independent founding haplotype regions.

*Disentangling founding time (* 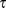 *) and current population size (* 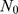 *)*: Using neutral diversity within the founding haplotypes used in this analysis, an estimate of 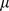 (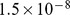 [25], as above), and estimates of 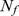 and 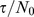, we could estimate 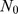 independently of 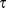. Via simulation, we found the expected number of generations since two lineages coalesce conditional on these lineages inheriting the same founding haplotype. We solved this to match the average 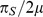 within haplotypes of our four exchangeable samples to obtain an estimate of 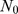.

## Supporting Information

Figure S1Patterns of variation in *Capsella*. 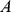 and 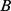) Mean number of pairwise differences at fourfold degenerate sites (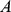), and ratio of diversity at synonymous to non-synonymous sites (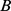) within *C. rubella*, *C. grandiflora*, and between species. The type of individuals compared is displayed on the x-axis, where G = Greek *C. rubell*a, O = Out-of- Greece *C. rubella*, and gr = *C. grandiflora*. Bars represent means and points represent single pairwise comparisons. 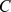 and 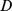) The frequency spectrum of synonymous sites segregating in *C. rubella* (polarized by the common *C. grandiflora* allele), within geographic comparisons (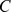) and across all samples (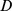).(EPS)

Figure S2Patterns of haplotype sharing in *C. rubella*. 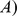 The proportion of the genome for which two individuals have inhertied the same or different founding haplotypes, or for which haplotype calls are ambiguous (e.g missing genotype makes haplotype calling problematic), by physical distance. 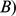 The proportion of regions of founding haplotype sharing of length X, by physical distance.(EPS)

Figure S3 Patterns of pairwise haplotype sharing across haplotype calling thresholds. As haplotype calling criteria become stricter, fewer pairwise comparisons can be assigned to the same (proportion below lines) or different (proportion above dashed lines) founding haplotypes, and more become ambiguous (compare to Figure 2A in the main text).
(EPS)

Figure S4 The allele frequency spectrum (proportion of singletons in three samples) across values for haplotype calling thresholds. Plotted separately for different types of trios – three Greek (red), three Out-of-Greece (green), two Greek and one out of Greece (blue), or two Out-of-Greece, and one Greek (purple) samples. Faint lines represent individual frequency spectra for comparisons within a trio. Thick lines represent mean values for a geographic comparison. Note that the observed fluctuations are much smaller than biologically informed differences (e.g. with and without accounting for haplotype labels, or all samples within vs. some samples outside of Greece, departures from expectations under the standard neutral model).
(EPS)

Figure S5

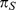
 (A) and 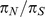 (B) within and among founding haplotypes across threshold values for haplotype assignment. Colored by geographic comparison. Lines and dashes represent comparisons within and among founding haplotypes, respectively.(EPS)

Figure S6Demographic inference across values for haplotype calling thresholds. The 95% confidence intervals and MLEs are displayed in red and blue, respectively. *A)* The inferred number of founding chromosomes, 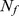. *B)* The inferred split time, 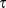. *C)* The inferred effective population size, 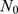.(EPS)

Figure S7 Haplotypic diversity and nucleotide diversity across the *C. rubella genome*. Nucleotide diversity at synonymous sites is in purple, and the inferred major haplotype frequency is in orange, while red points are below regions putatively containing more than two founding haplotypes. Each data point represents a 10 kb window with a 2 kb slide. Each of the eight panels represents a different chromosome.
(PDF)

Figure S8 Genome-wide individual heterozygosity in *C. rubella*. We separately display individual heterozygosity at synonymous sites in regions inferred to be allozygous (blue) or autozygous (red) for each *C. rubella* individual (noted on the x-axis). The dotted line represents pairwise sequence diversity between *C. rubella* samples at synonymous sites for reference.
(PDF)

Figure S9 Genome-wide individual heterozygosity in *C. rubella*. We label allozygous regions by eye (Letters A–F represent individuals, and numbers 1–8 represent chromosomes – see figure titles). Blue and red points display sites heterozygous in *C. rubella* or both species, respectively, while singleton sites are presented in grey. Dotted lines separate regions inferred to be autozygous and allozygous. The cumulative number of heterozygous genotypes is plotted on the y-axis, and the physical position is displayed on the x-axis. We infer a region to be allozygous when this slope is relatively large.
(PDF)

Figure S10Evidence for more than two founding haplotypes in *C. rubella*. Nucleotide diversity (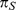) within *C. rubella* trios in genomic regions putatively containing more than two founding haplotypes. Each pairwise comparison between individuals inferred to have inherited alternative founding haplotypes is labelled in red, blue, or orange. Grey lines illustrate pairwise comparisons between individuals inferred to have inherited the same founding haplotype. Dashed lines mark 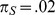 – the diversity expected between haplotypes on the basis of our estimates from regions with exactly two founding haplotypes. Dotted lines mark the borders of genomic regions in which we infer more than two founding haplotypes. Each of the nine panels in Figure S10 comprises a different region putatively containing three founding haplotypes (note that the chromosome, genomic position, and trio changes in each panel).(EPS)

Table S1Validation of *C. rubella* genotype calls by Sanger sequencing. 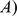 Genomic regions targeted for Sanger sequencing. The chromosome, genomic location, orientation of each ORF, and the individuals sequenced is noted in each column. 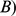 Concordance between Sanger and RNA-seq genotypes calls, split by individual. Numbers refer to the number of inferred non-reference alleles (e.g. 0 = homozygous for the reference, 1 = heterozygous, 2 = homozygous non-reference), and NAs mark missing data or data that did not pass QC. Sanger genotypes are presented before, and RNA-Seq genotypes are presented after the ‘/’. Note the minimal discordance between genotype calls by technology.(PDF)

Table S2 Influence of founding haplotype stringency on founding haplotype assignment. The proportion of the genome in which we infer less than two founding haplotypes and no ambiguity in founding haplotype assignment as we change the X consecutive SNPS spanning Y base pairs required for founding haplotype assignment.
(PDF)

Table S3 Summary of synonymous and non-synonymous variation within and among *C. rubella's* founding haplotypes. All = comparison between all *C. rubella* samples, G/G = comparison between two *C. rubella* samples from Greece, G/O = comparison betweenGreek and Out-of-Greek *C. rubella* samples, O/O = comparison between two *C. rubella* samples from Out-of-Greece.
(PDF)

Text S1Supplementary text: Additional details about the data and analyses presented above. 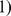 Data summary without reference to haplotype. 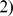 The 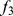 test shows no signal of recent introgression. 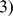 Founding haplotype sharing by physical distance. 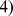 Validation of RNA-Seq genotypes by Sanger sequencing. 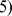 Robustness of results to thresholds for calling haplotype. 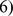 A summary of diversity within and among founding haplotypes. 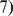 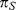 and major haplotype frequency of the across all chromosomes. 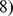 Putatively allozygous regions. 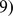 Third haplotype candidate regions.(PDF)
