# Supplementary figures and images for: Genomic Identification of Founding Haplotypes Reveals the History of the Selfing Species Capsella rubella
Source: PLoS Genet. 2013 Sep 12;9(9):e1003754. doi: 10.1371/journal.pgen.1003754 (PMC3772084; doi:10.1371/journal.pgen.1003754)

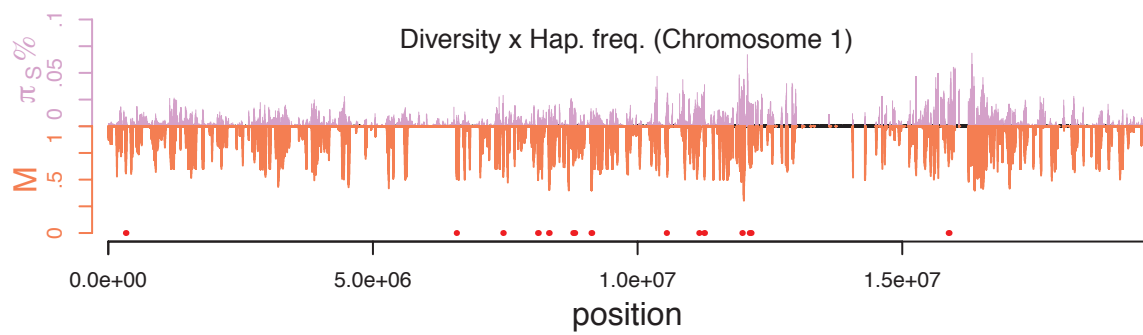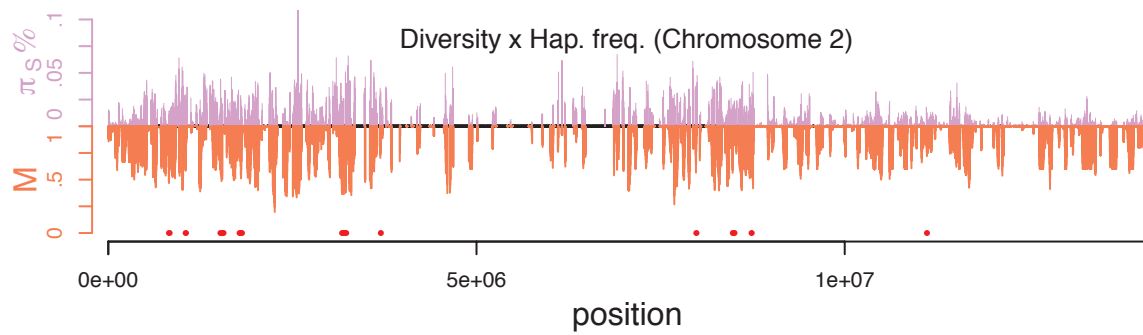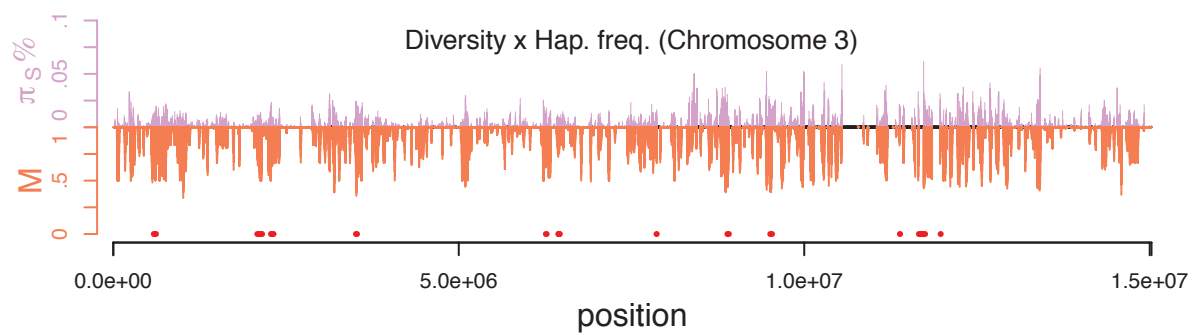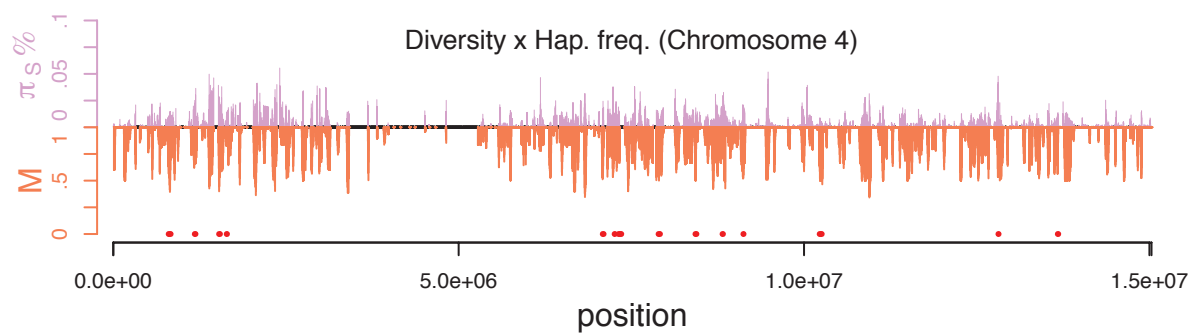

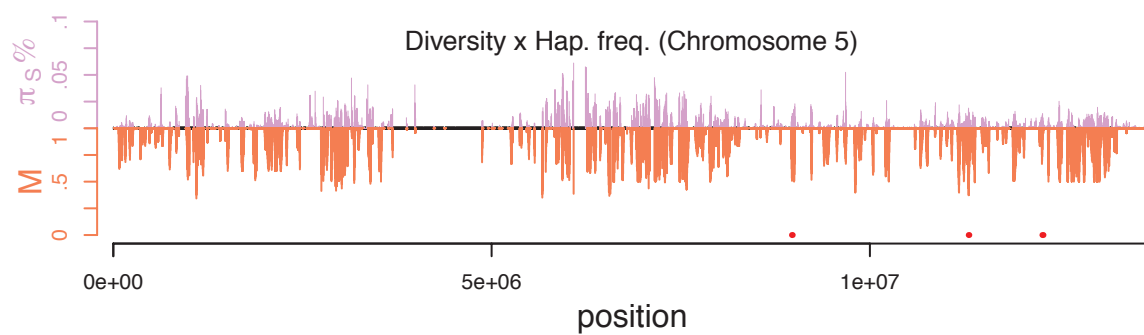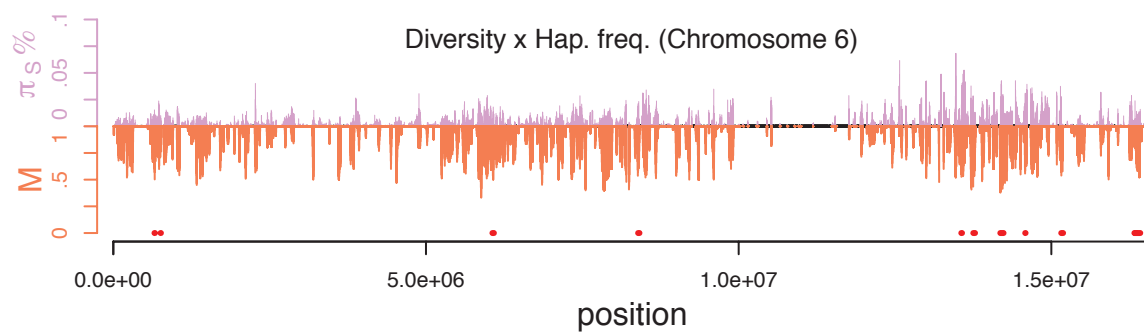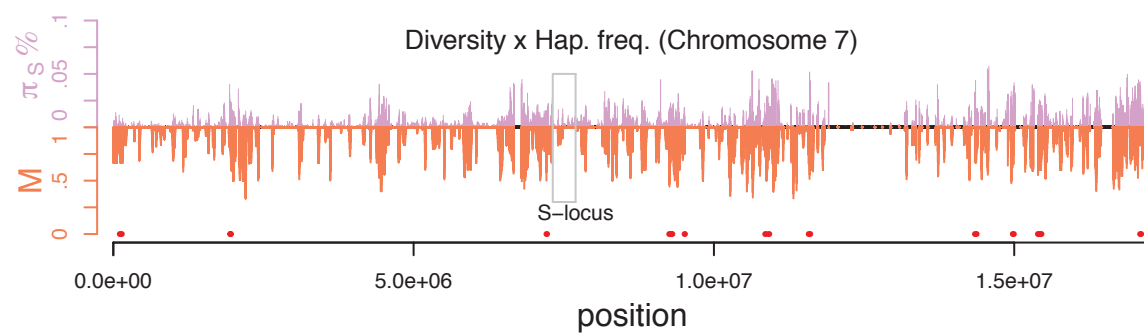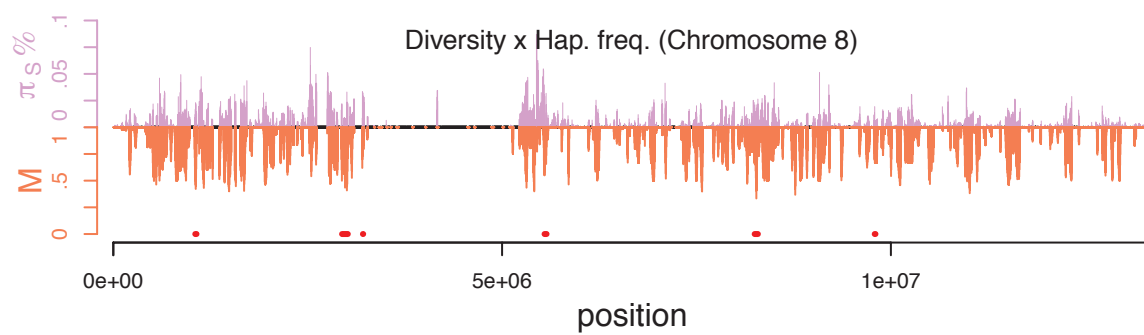

Supplement: Figure S7 — Haplotypic diversity and nucleotide diversity across the C. rubella genome. Nucleotide diversity at synonymous sites is in purple, and the inferred major haplotype frequency is in orange, while red points are below regions putatively containing more than two founding haplotypes. Each data point represents a 10 kb window with a 2 kb slide. Each of the eight panels represents a different chromosome. (PDF) [file pgen.1003754.s007.pdf]

# Individual heterozygosity in autozygous and allozygous genomic regions

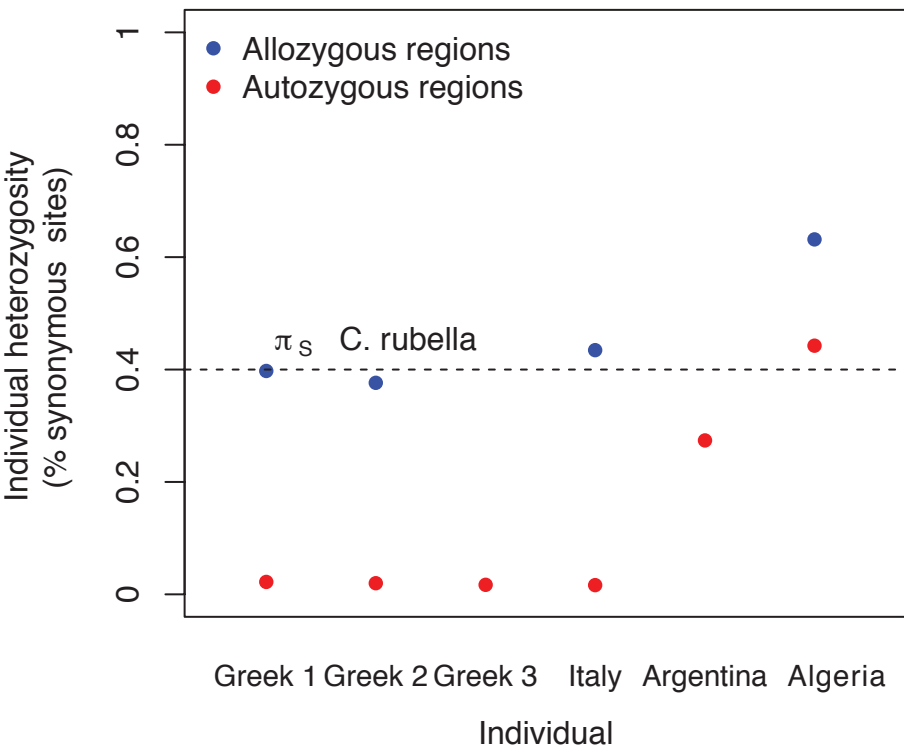

Supplement: Figure S8 — Genome-wide individual heterozygosity in C. rubella. We separately display individual heterozygosity at synonymous sites in regions inferred to be allozygous (blue) or autozygous (red) for each C. rubella individual (noted on the x-axis). The dotted line represents pairwise sequence diversity between C. rubella samples at synonymous sites for reference. (PDF) [file pgen.1003754.s008.pdf]
