# Supplementary material for: Genomic Identification of Founding Haplotypes Reveals the History of the Selfing Species Capsella rubella
Source: PLoS Genet. 2013 Sep 12;9(9):e1003754. doi: 10.1371/journal.pgen.1003754 (PMC3772084; doi:10.1371/journal.pgen.1003754)

S9_A.1)　　　Cr81 (Greek), Chromosome 1

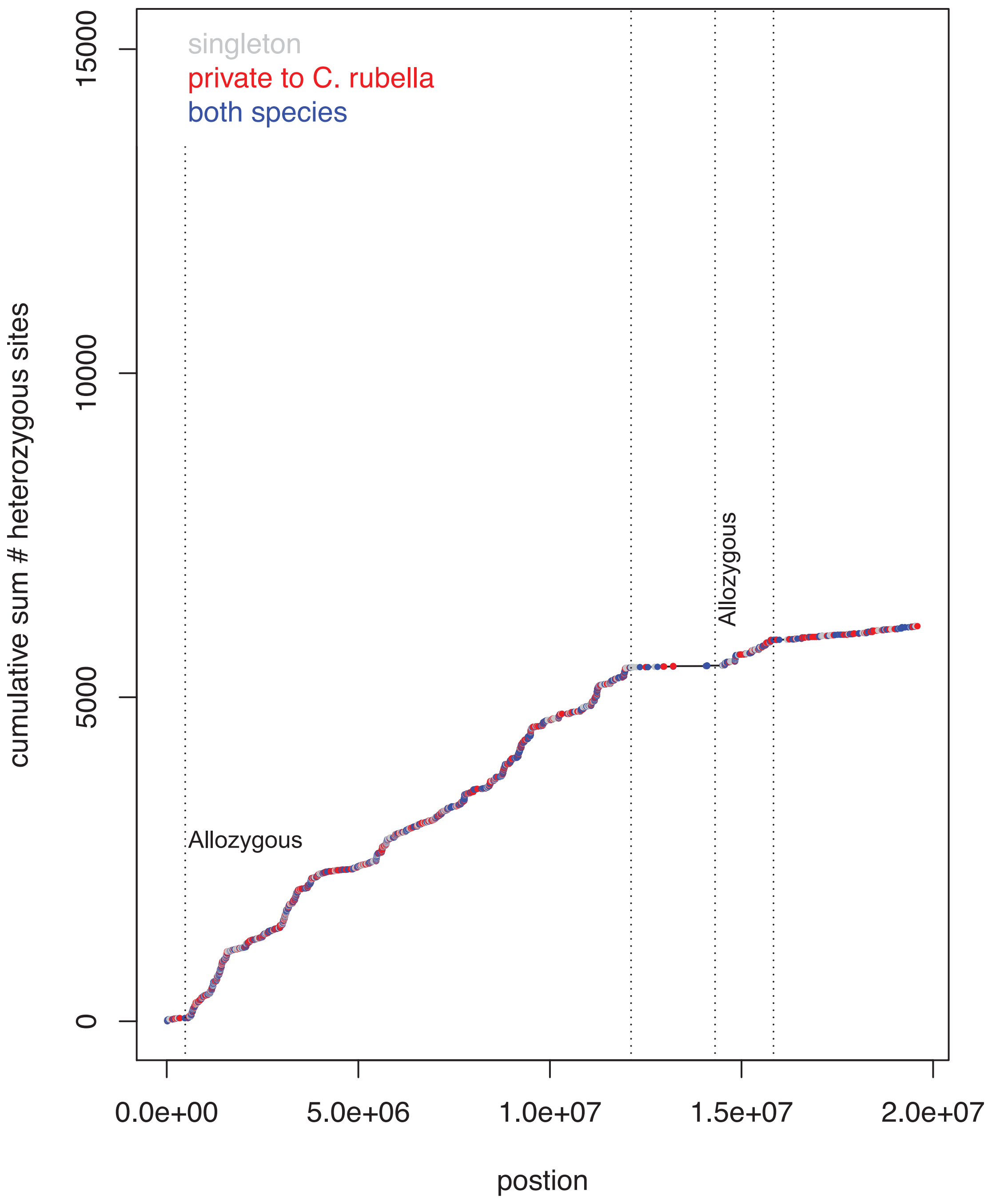

S9_A.2) **Cr81 (Greek), Chromosome 2**

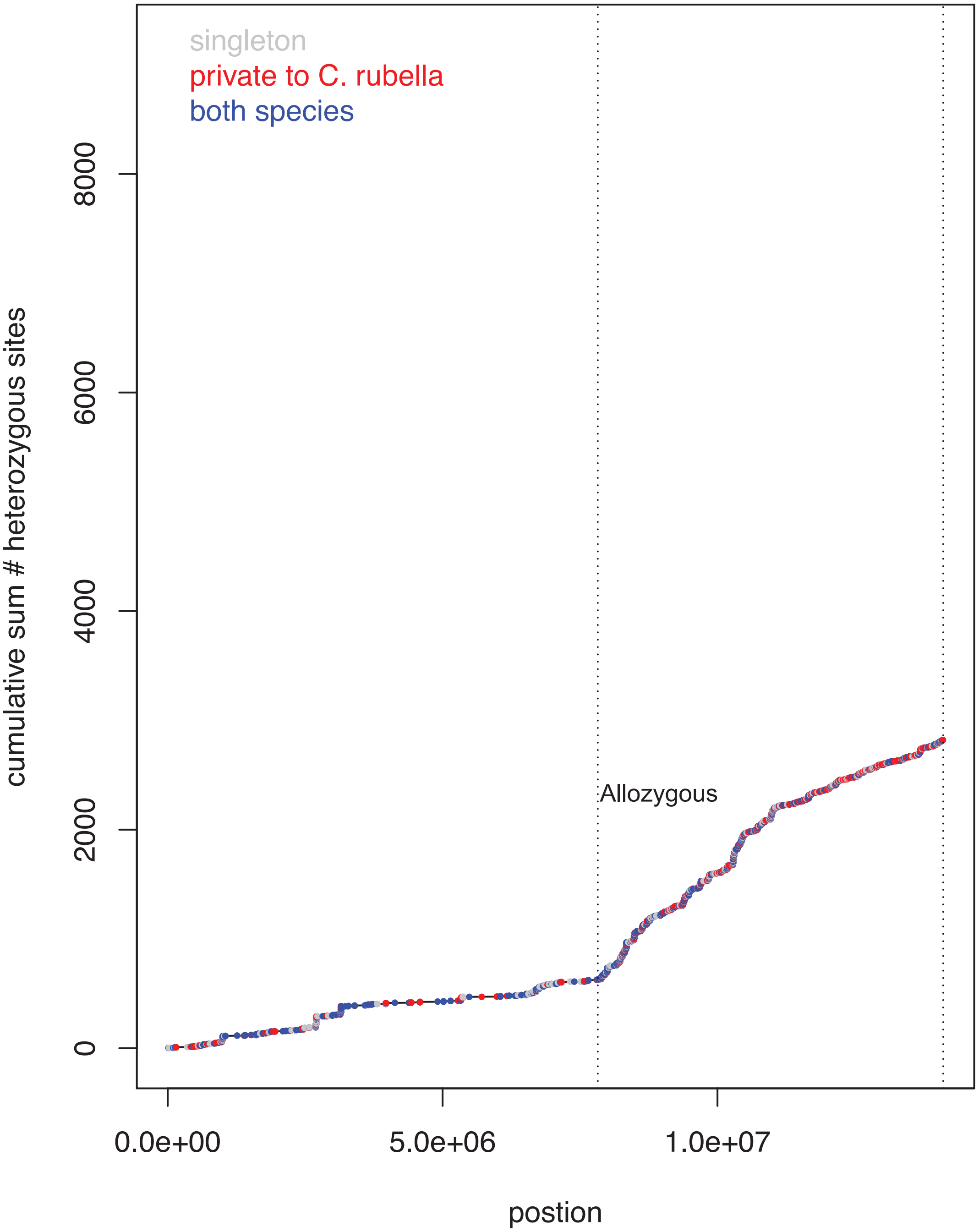

S9_A.3) Cr81 (Greek), Chromosome 3

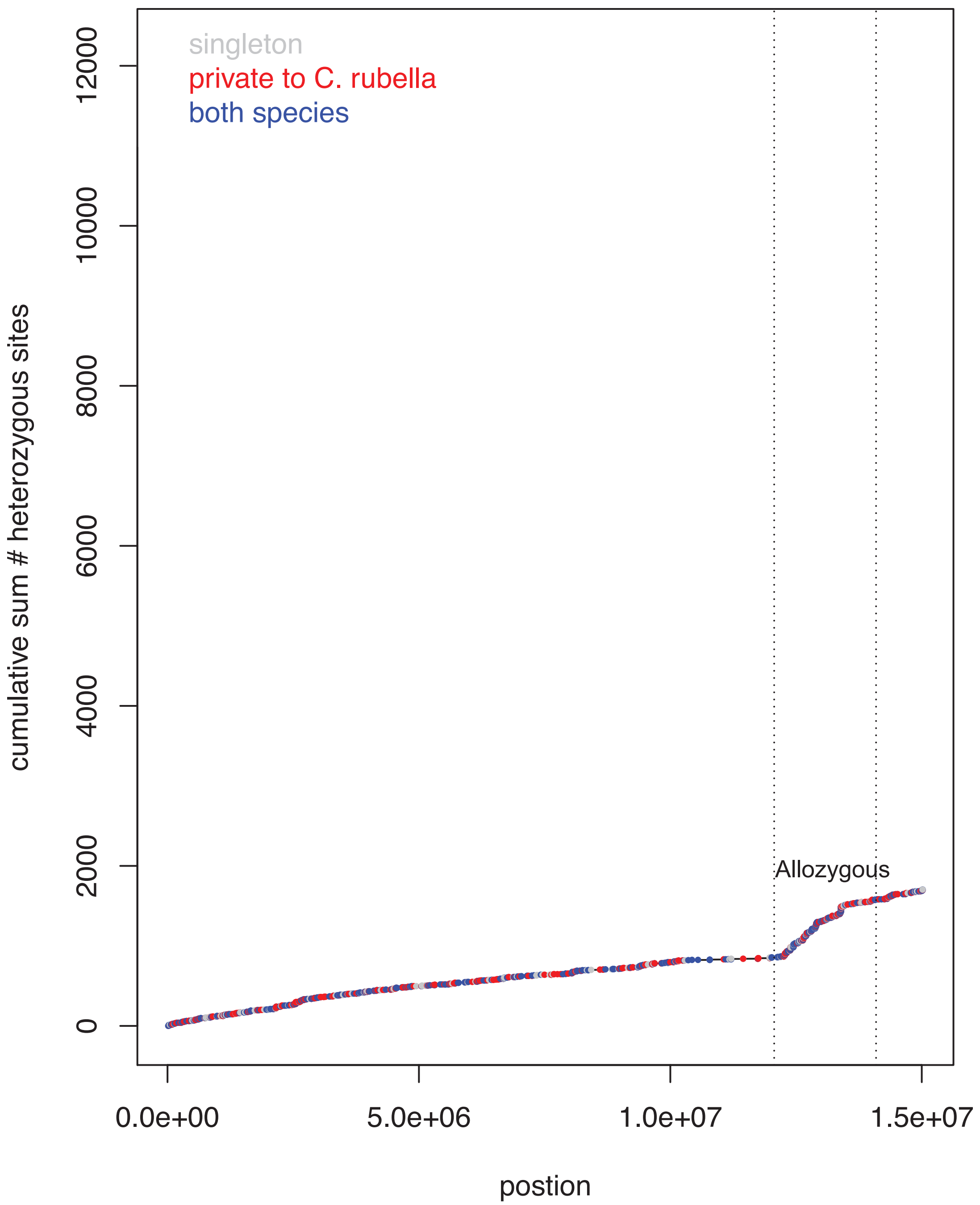

S9_A.4) Cr81 (Greek), Chromosome 4

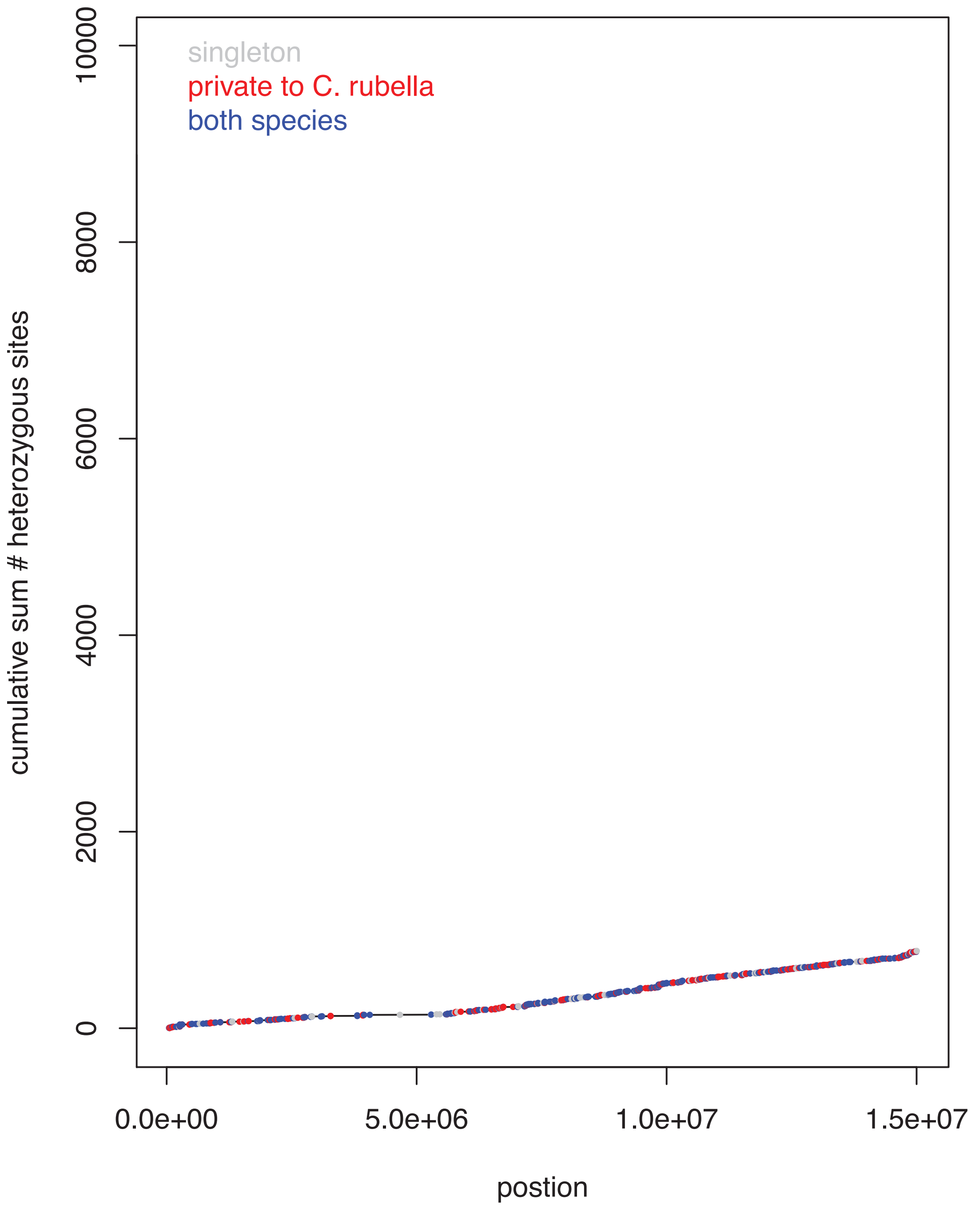

S9_A.5) Cr81 (Greek), Chromosome 5

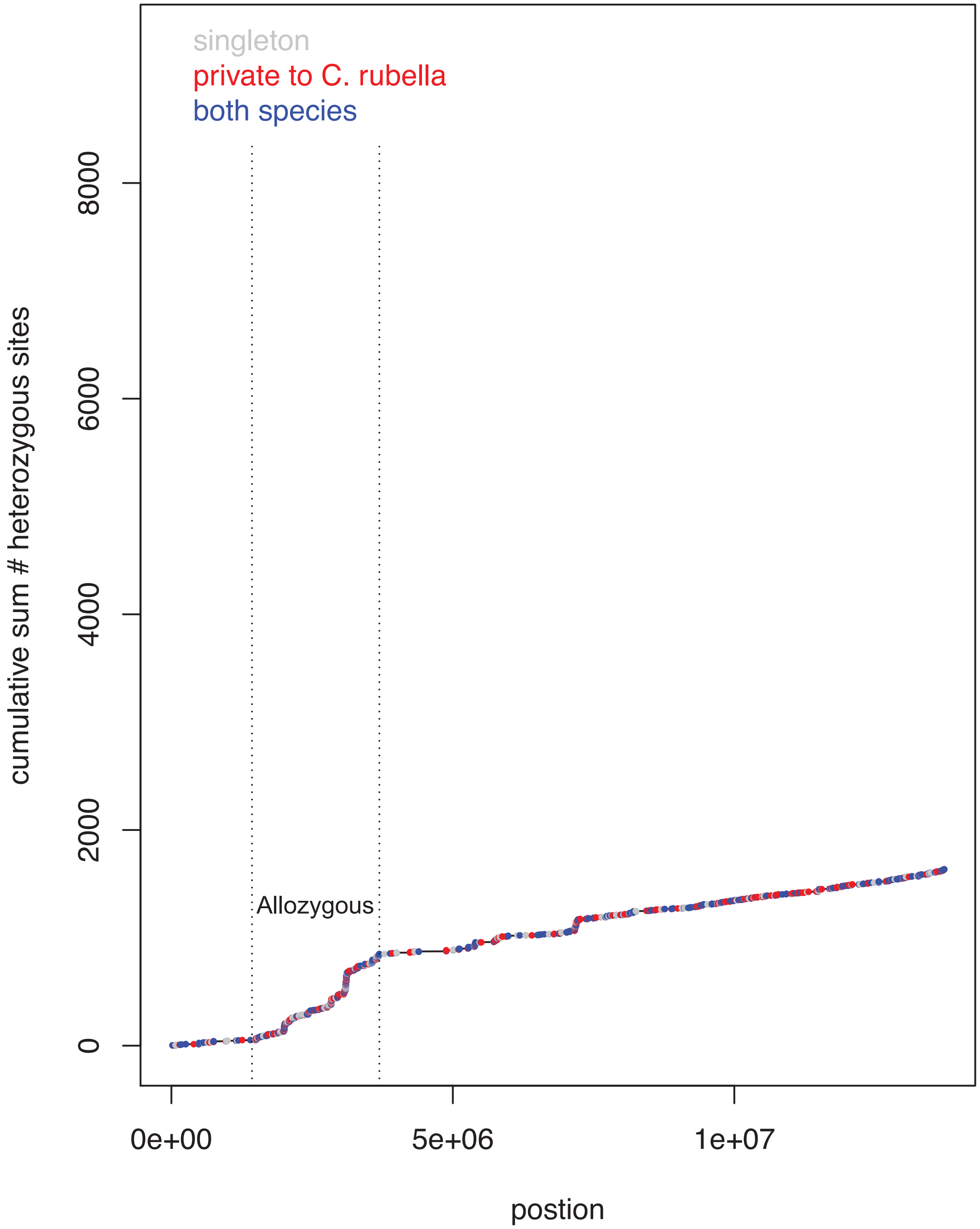

S9_A.6) **Cr81 (Greek), Chromosome 6**

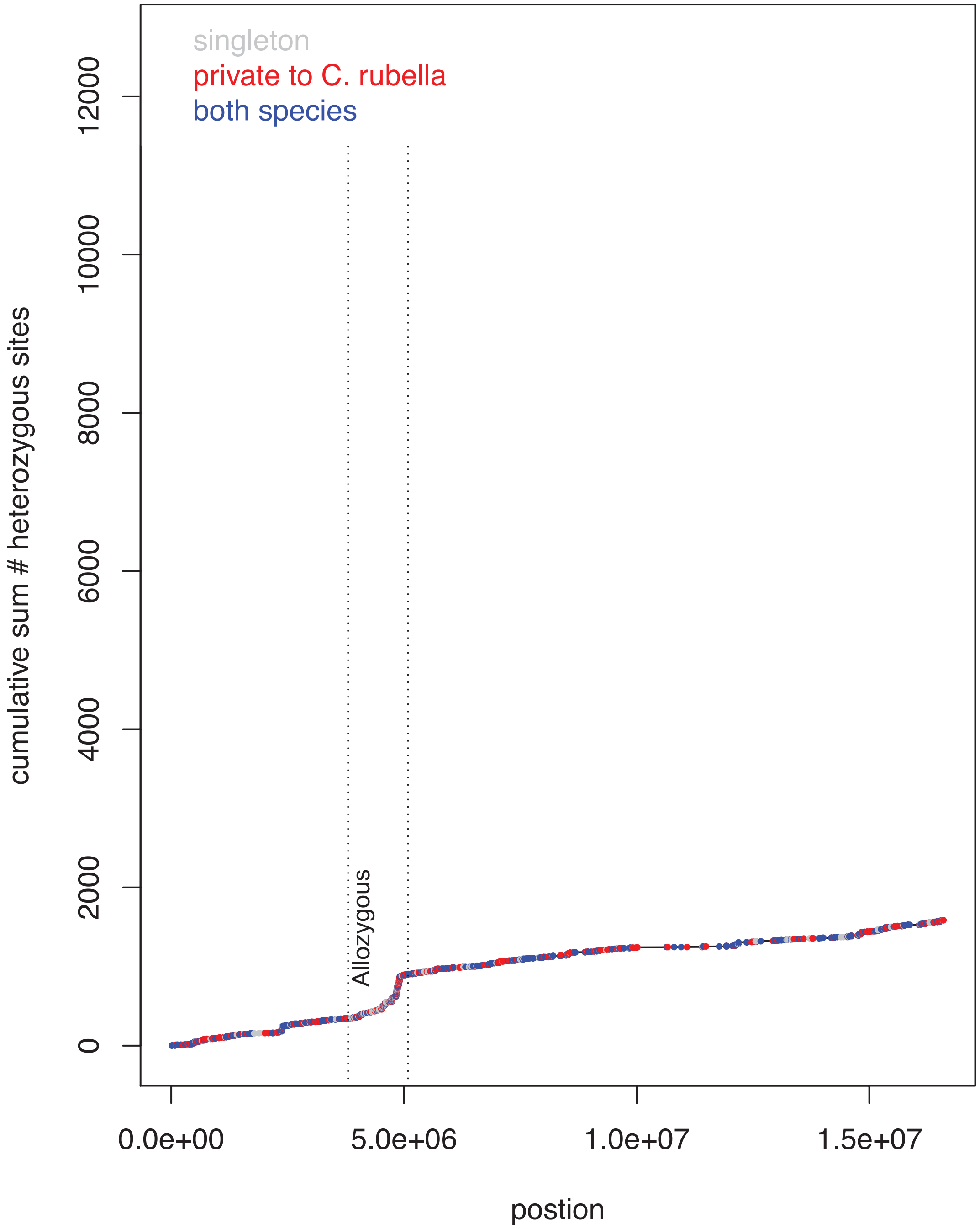

S9_A.7) **Cr81 (Greek), Chromosome 7**

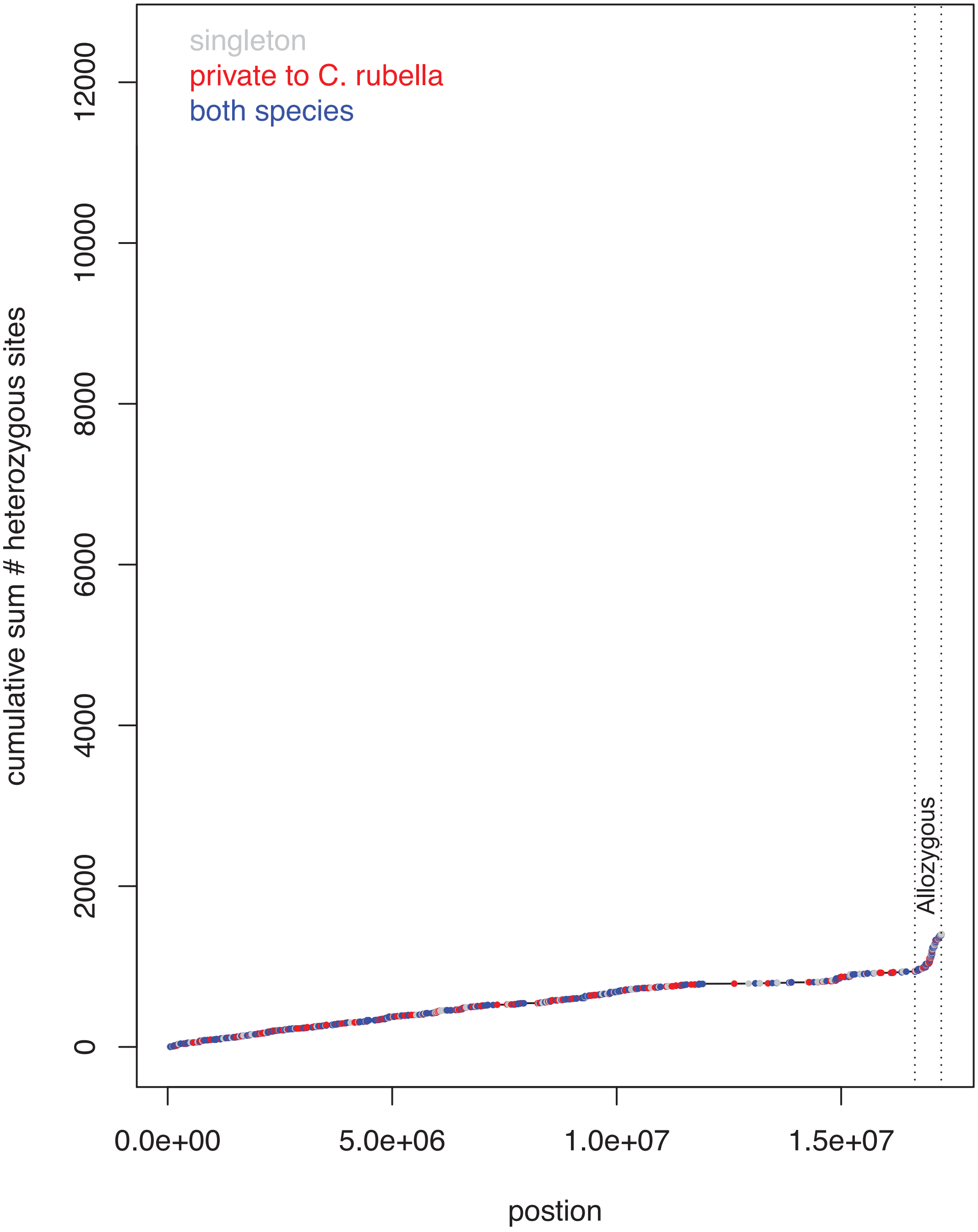

S9_A.8) **Cr81 (Greek), Chromosome 8**

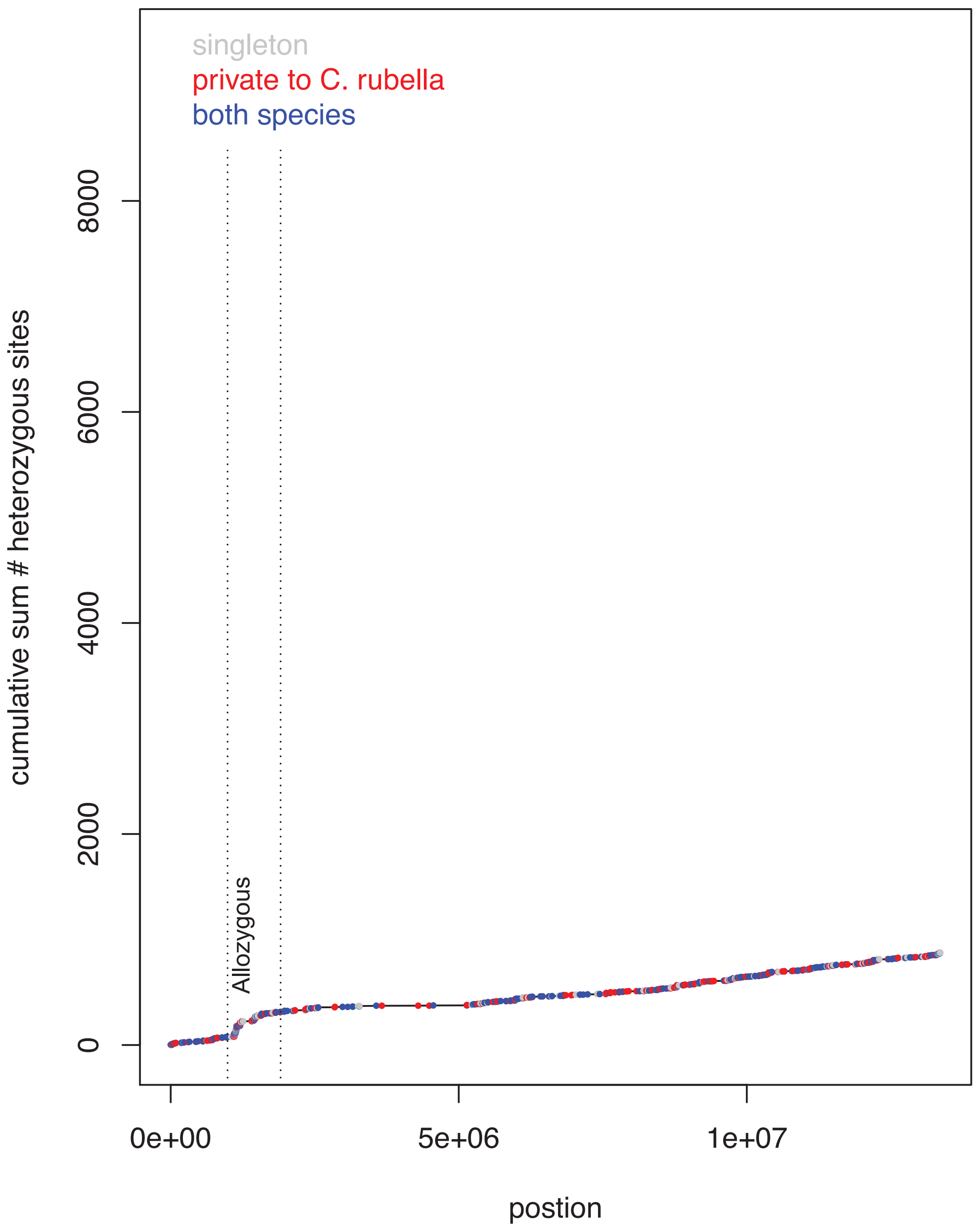

S9_B.1) Cr1Gr1 (Greek, lab inbred), Chromosome 1

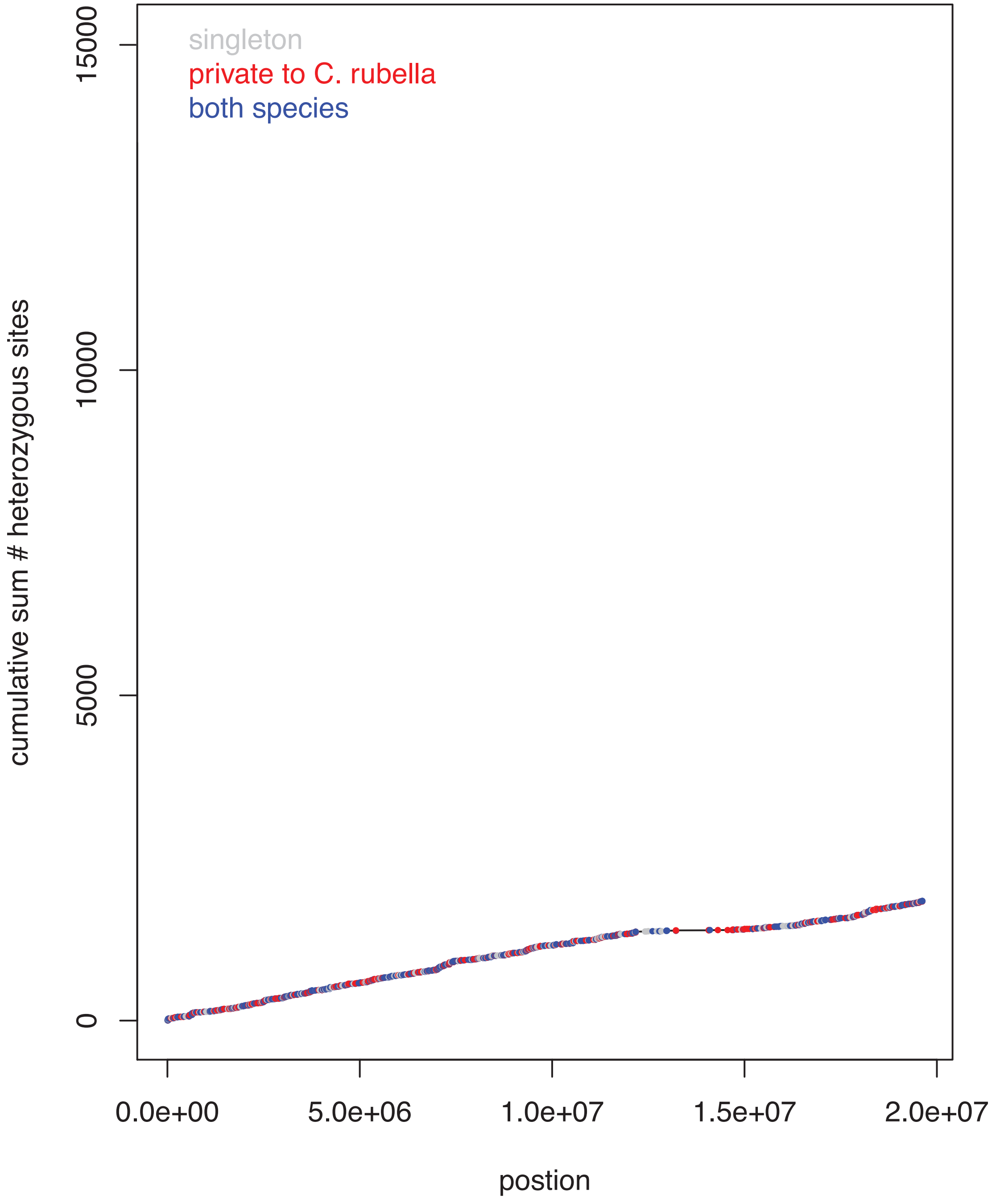

S9_B.2) Cr1Gr1 (Greek, lab inbred), Chromosome 2

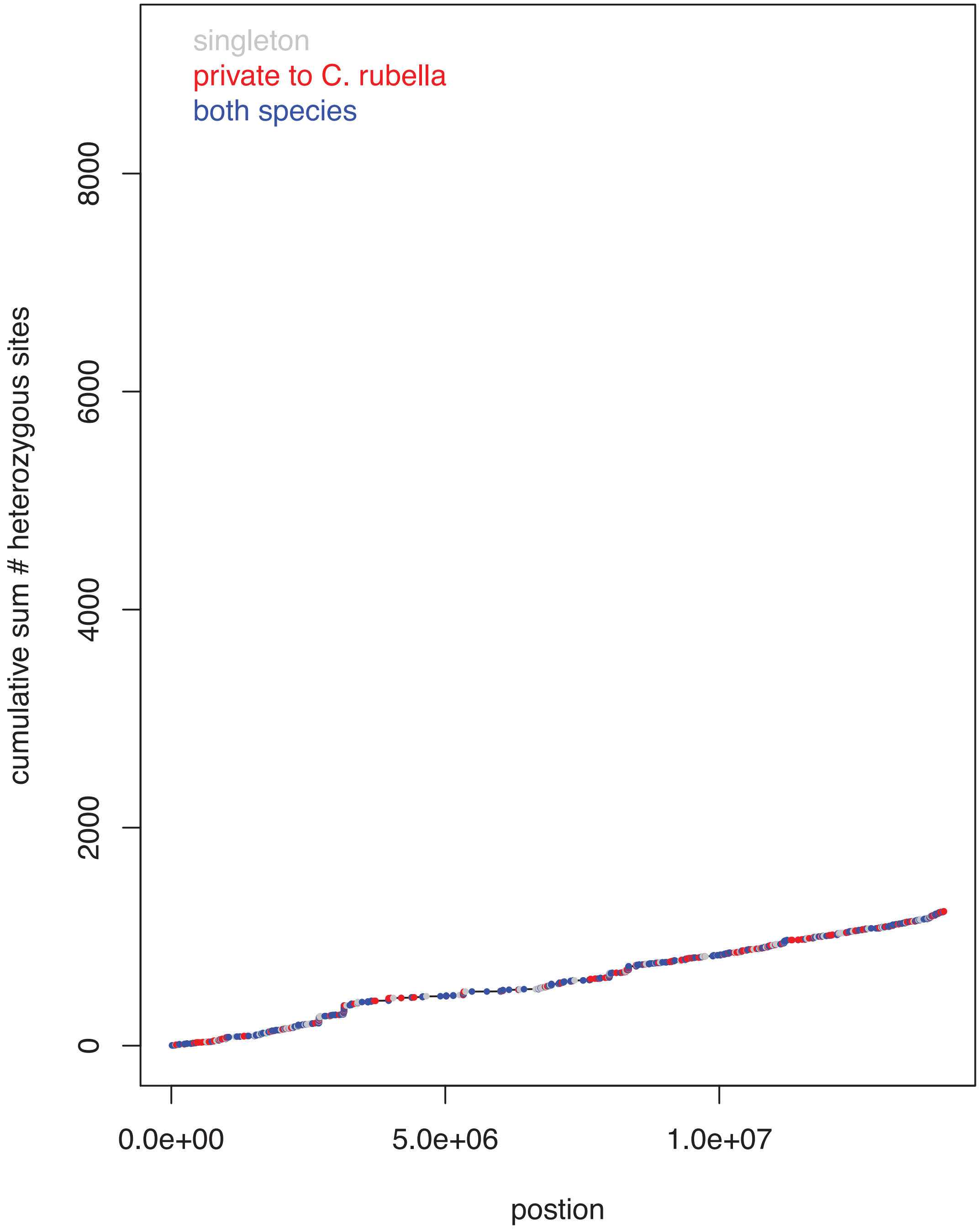

S9_B.3) Cr1Gr1 (Greek, lab inbred), Chromosome 3

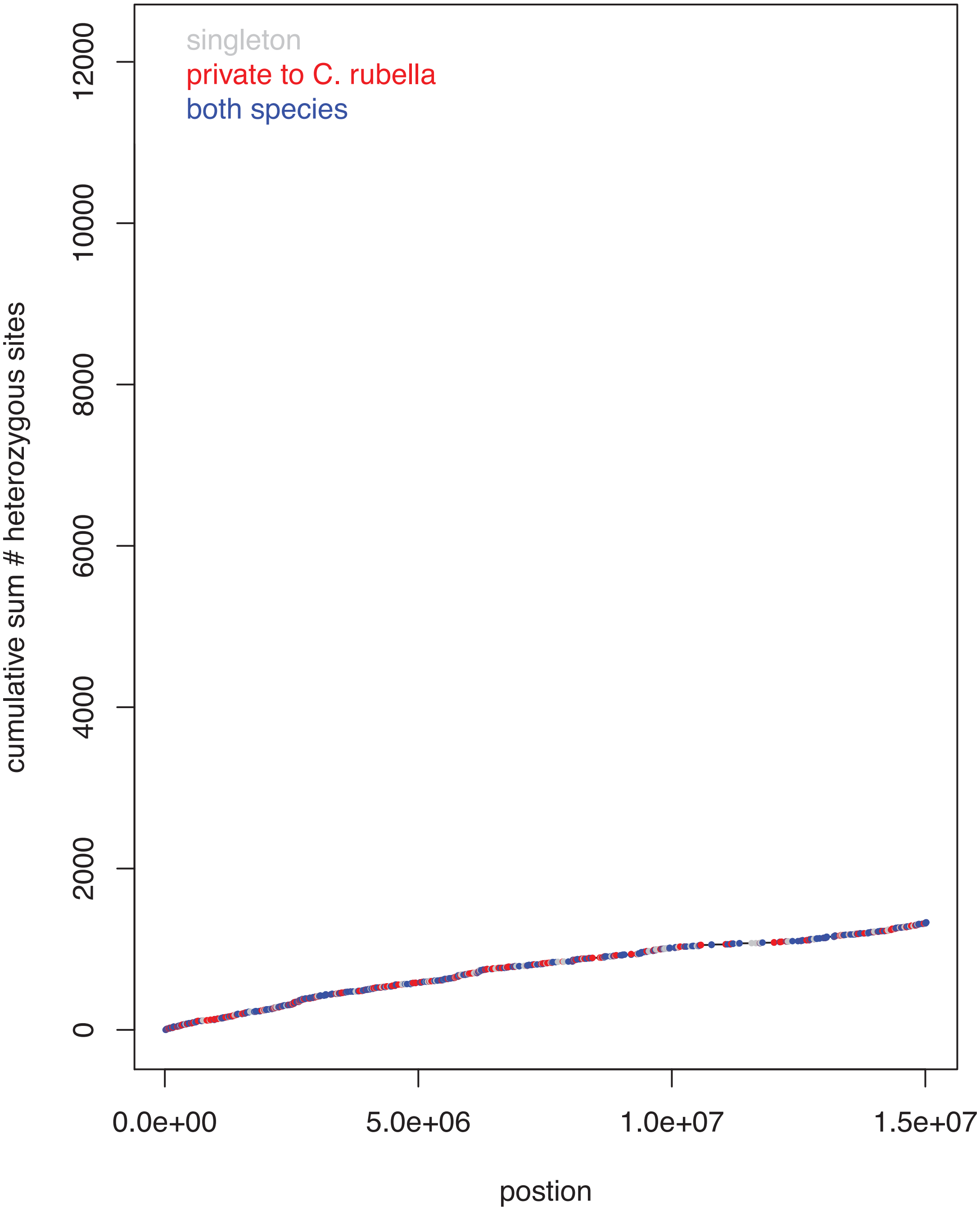

S9_B.4) Cr1Gr1 (Greek, lab inbred), Chromosome 4

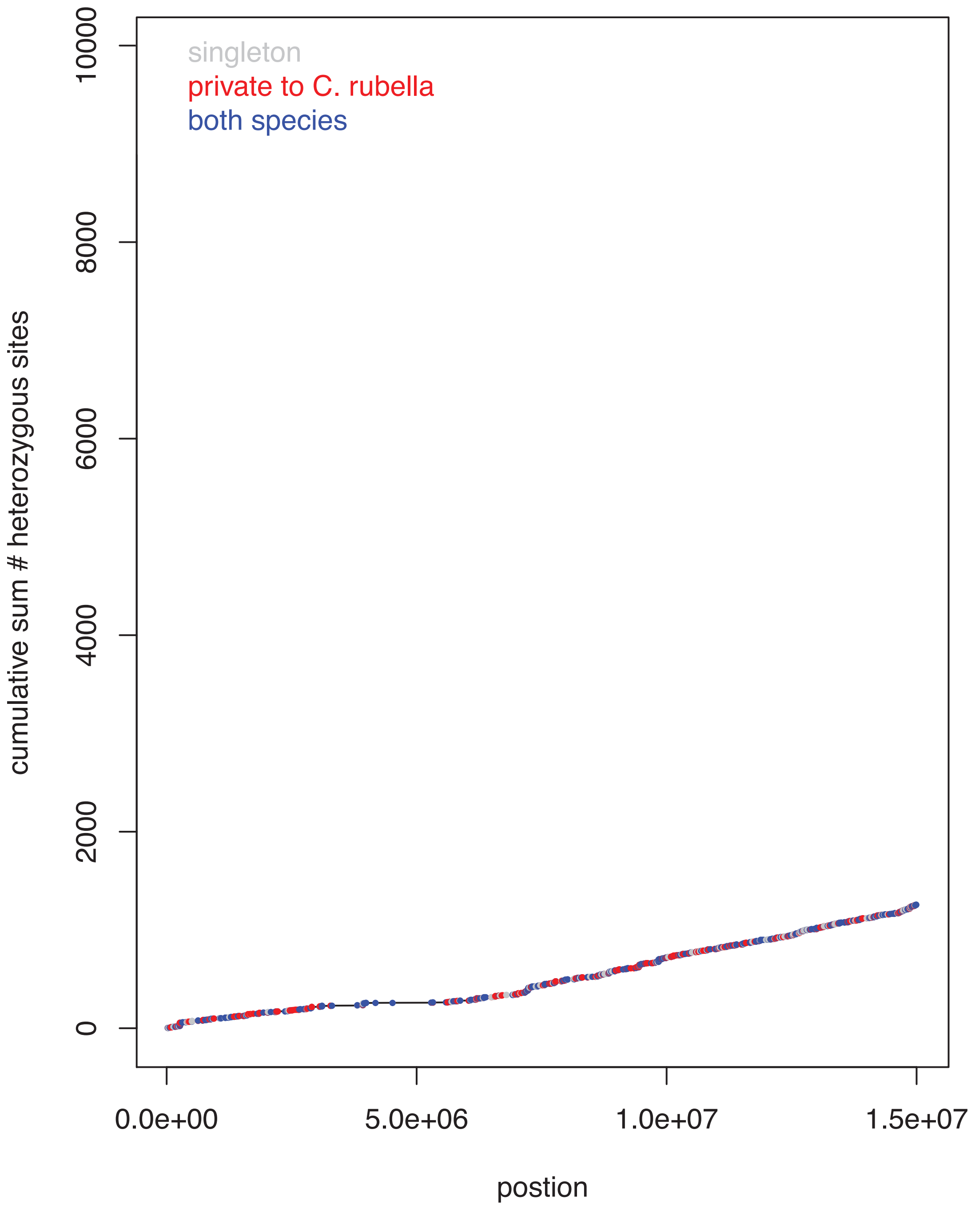

S9_B.5) Cr1Gr1 (Greek, lab inbred), Chromosome 5

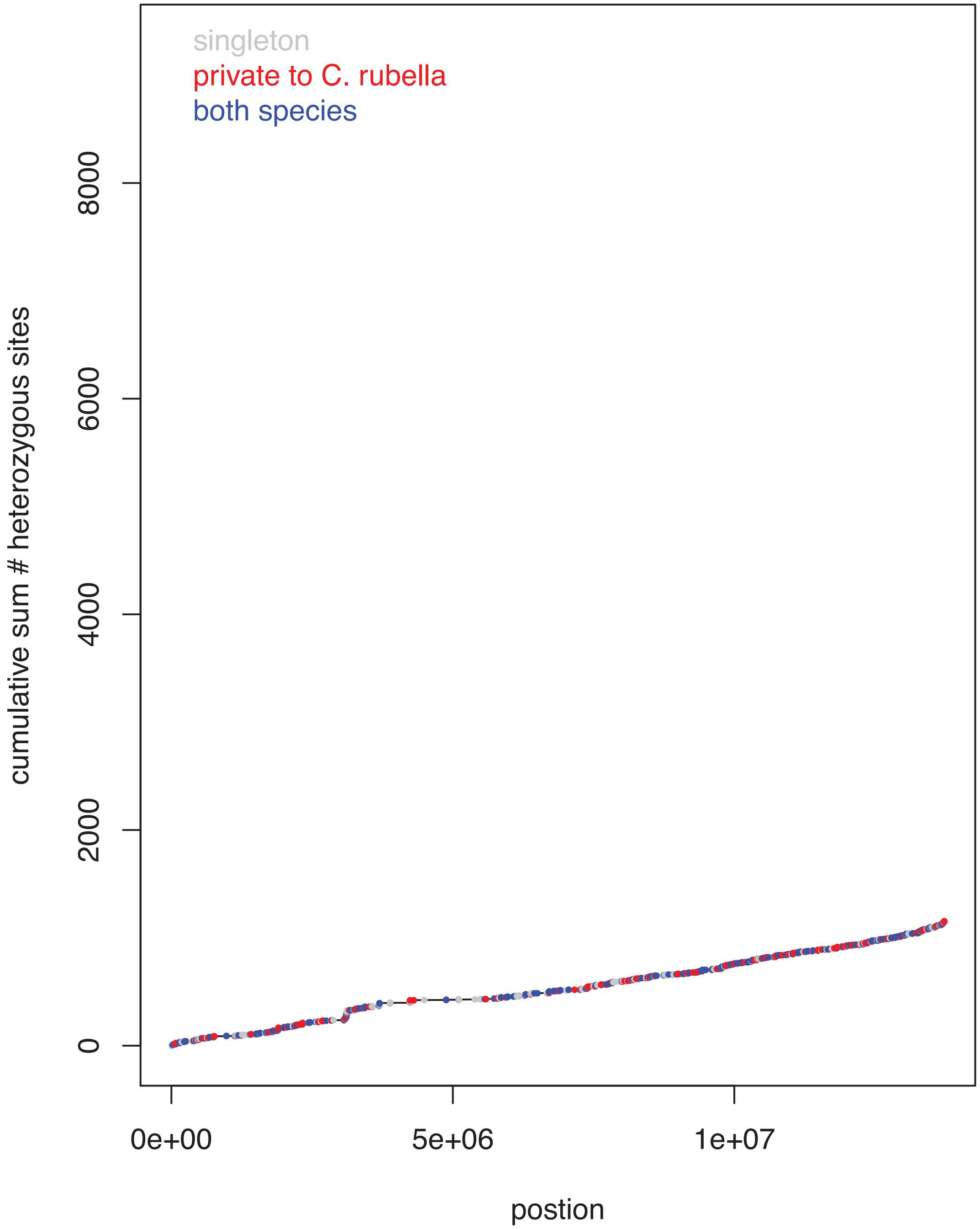

S9_B.6) Cr1Gr1 (Greek, lab inbred), Chromosome 6

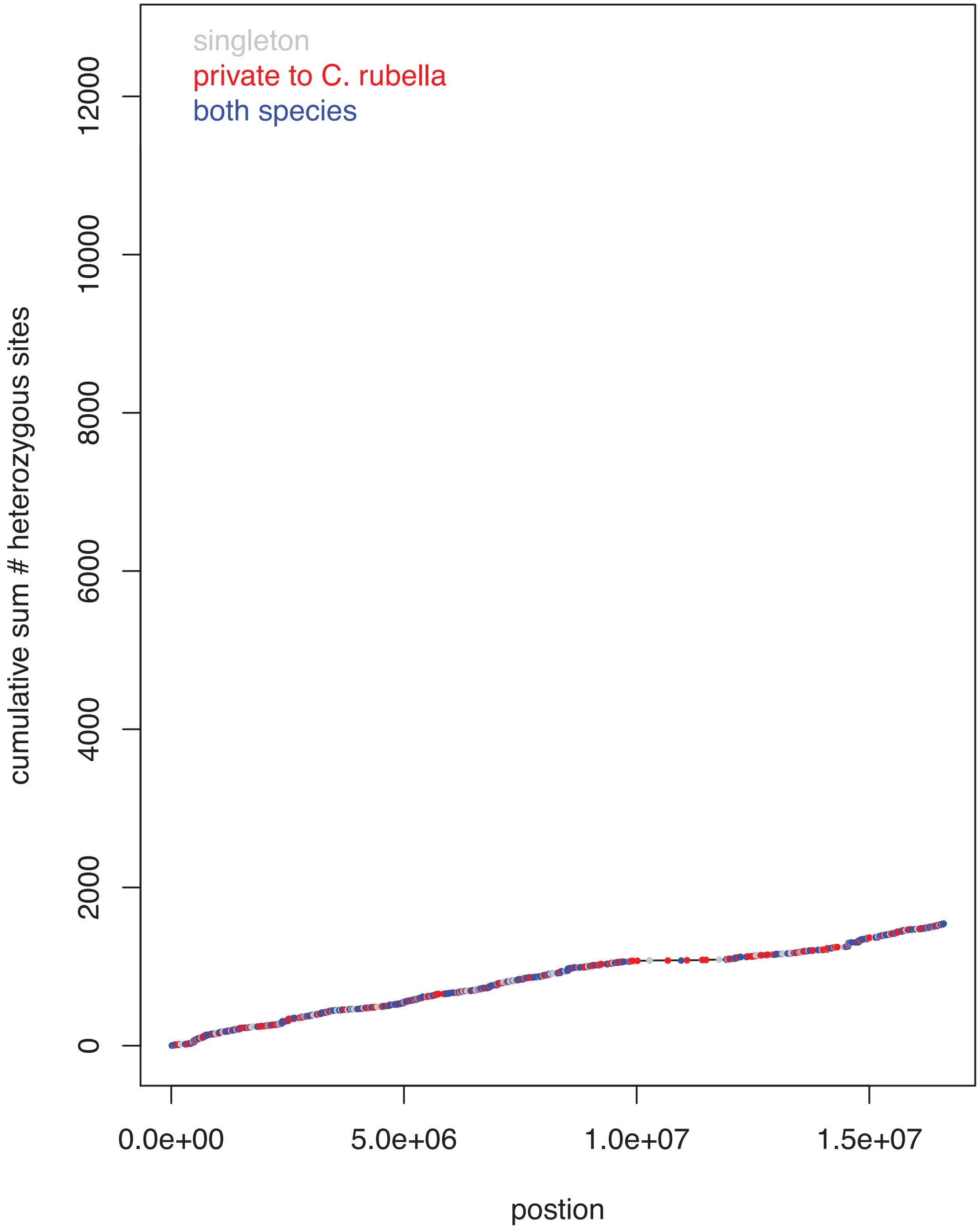

S9_B.7) Cr1Gr1 (Greek, lab inbred), Chromosome 7

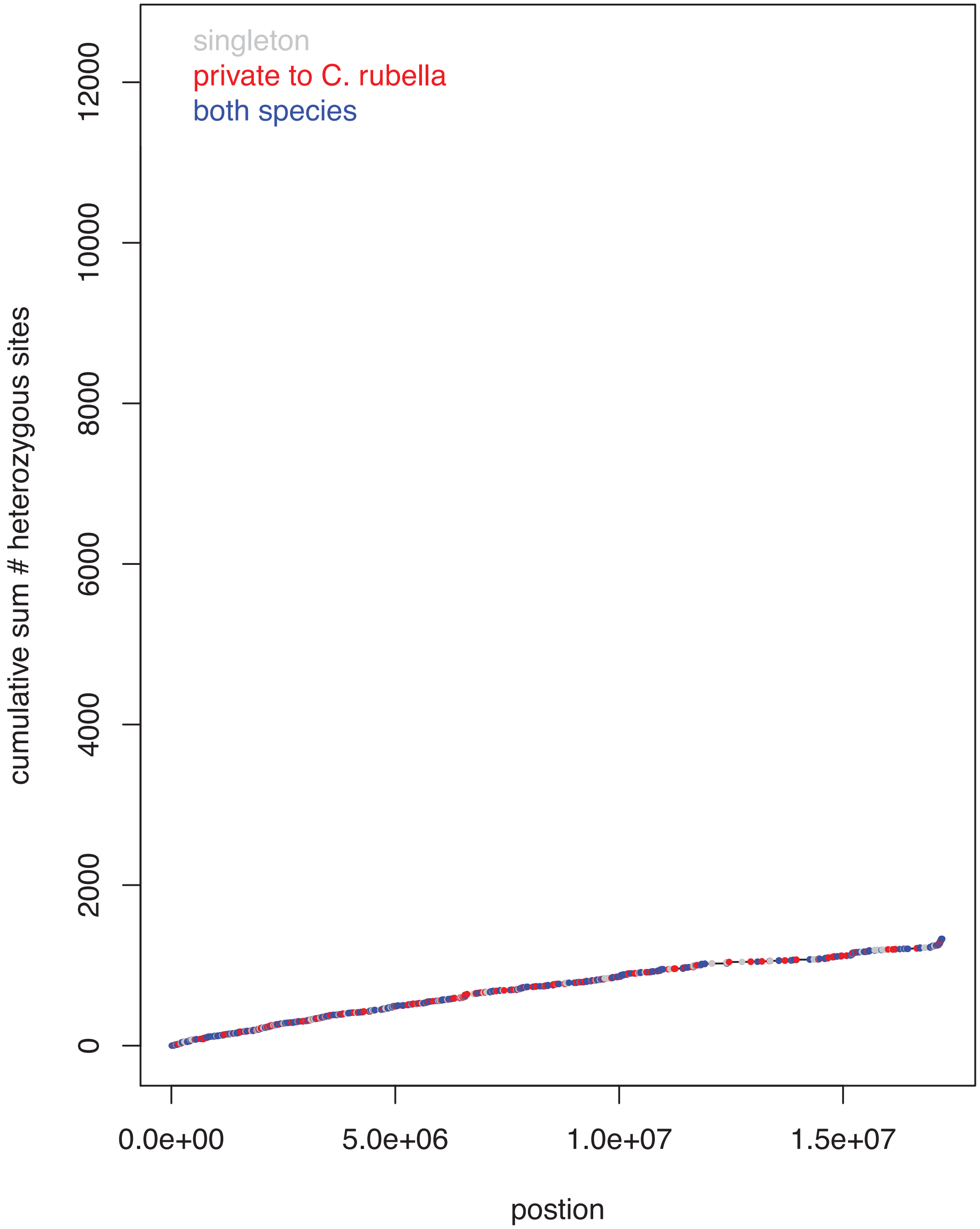

S9_B.8) Cr1Gr1 (Greek, lab inbred), Chromosome 8

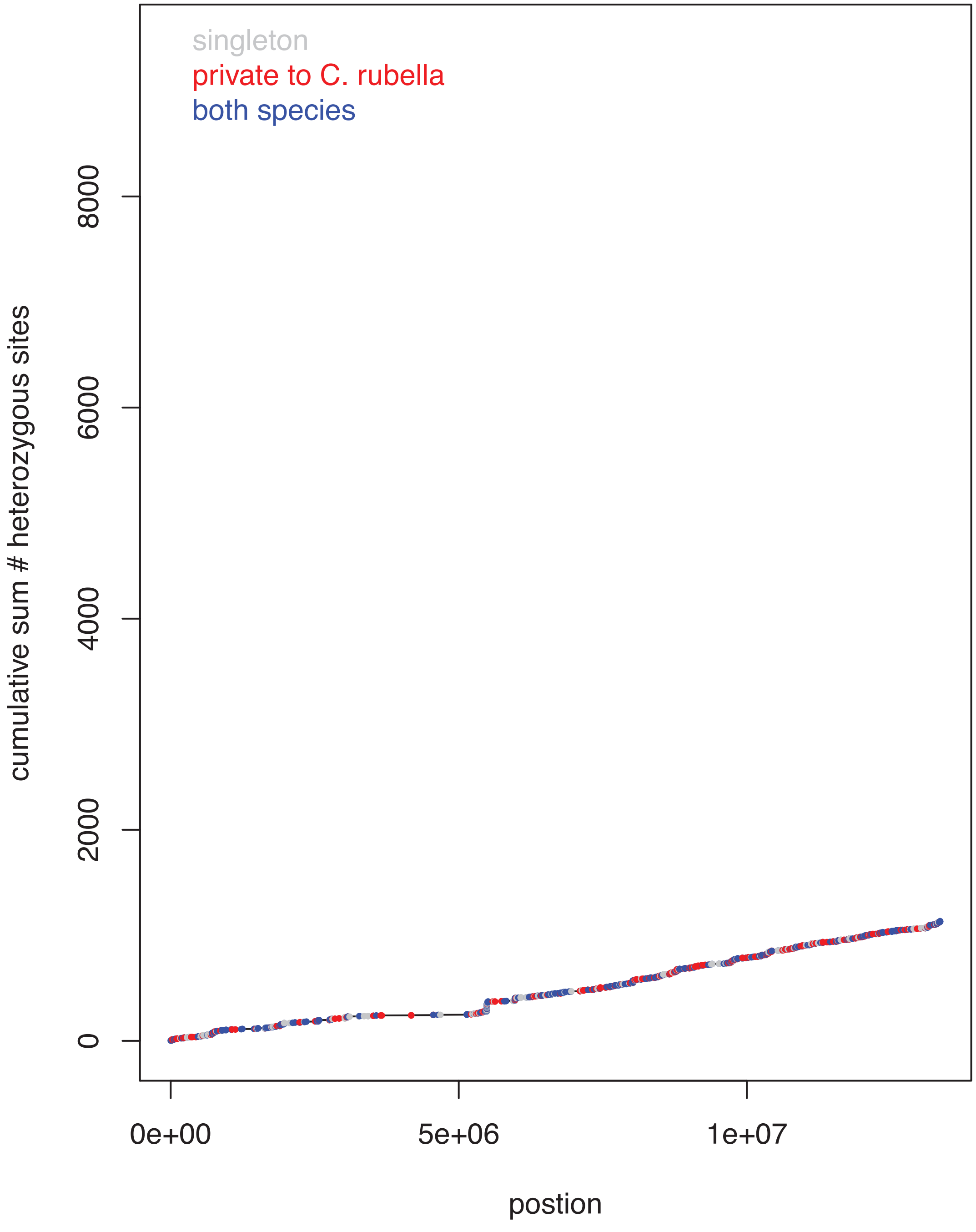

S9_C.1) **Cr34 (Italian), Chromosome 1**

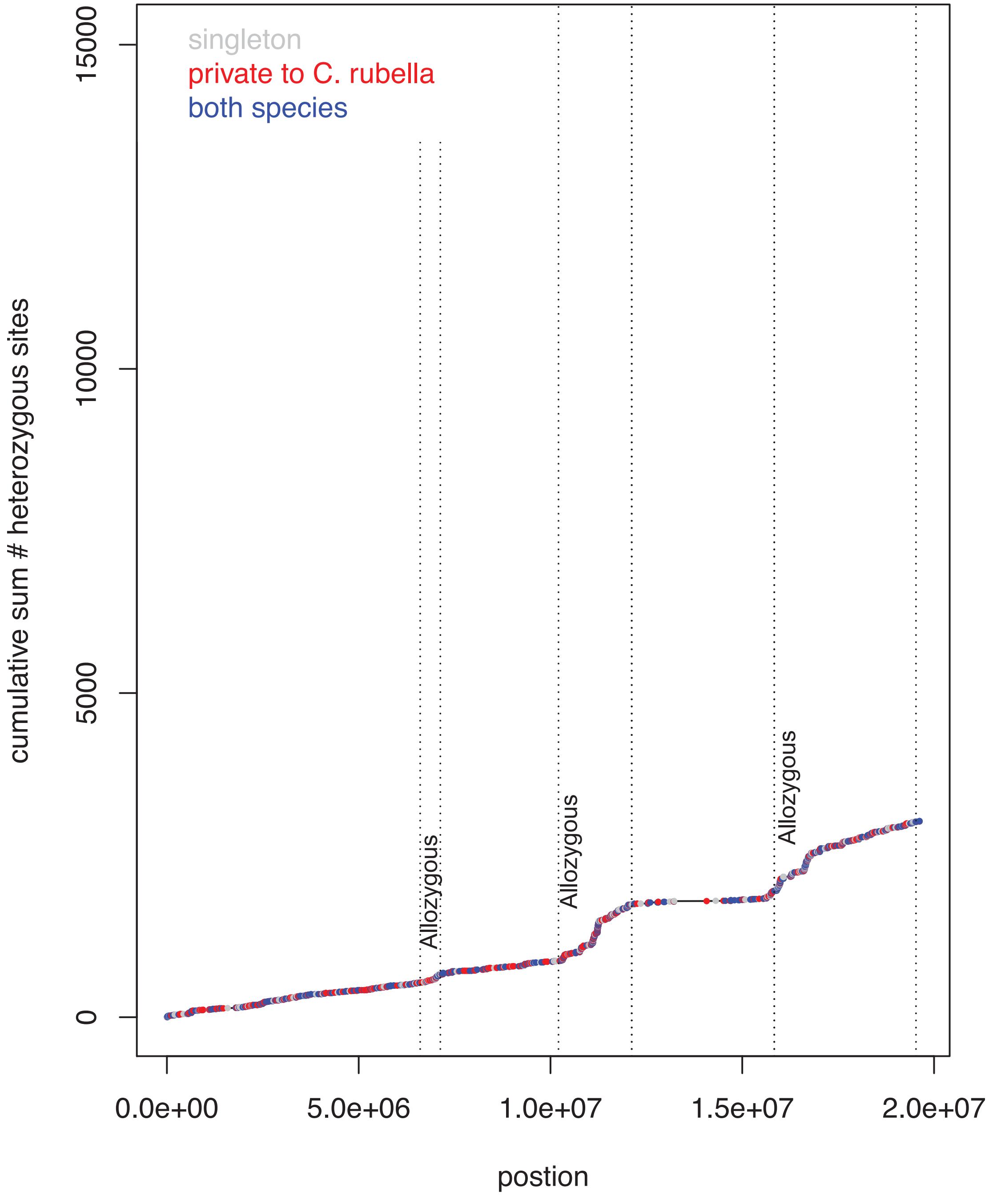

S9_C.2) Cr34 (Italian), Chromosome 2

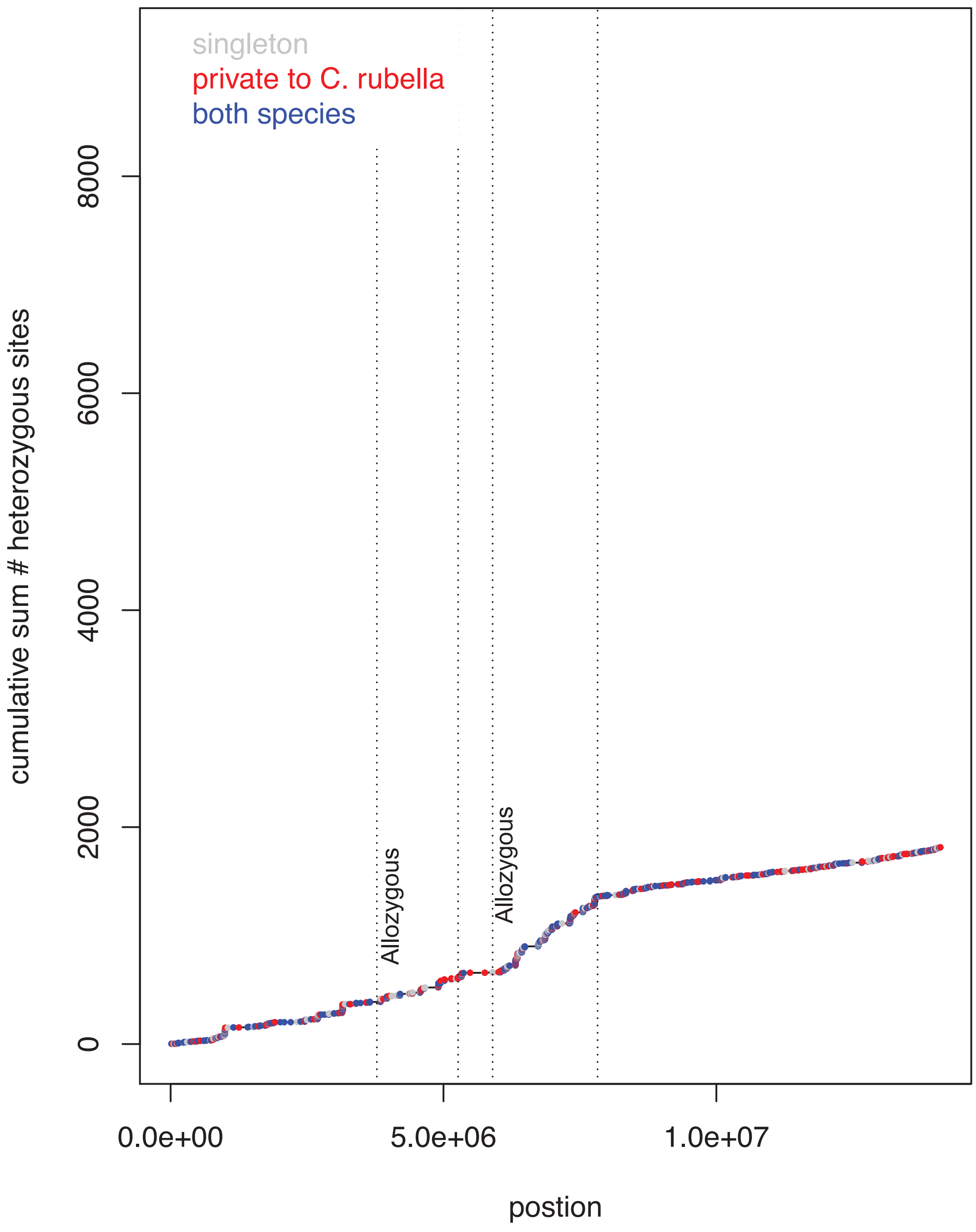

S9_C.3) **Cr34 (Italian), Chromosome 3**

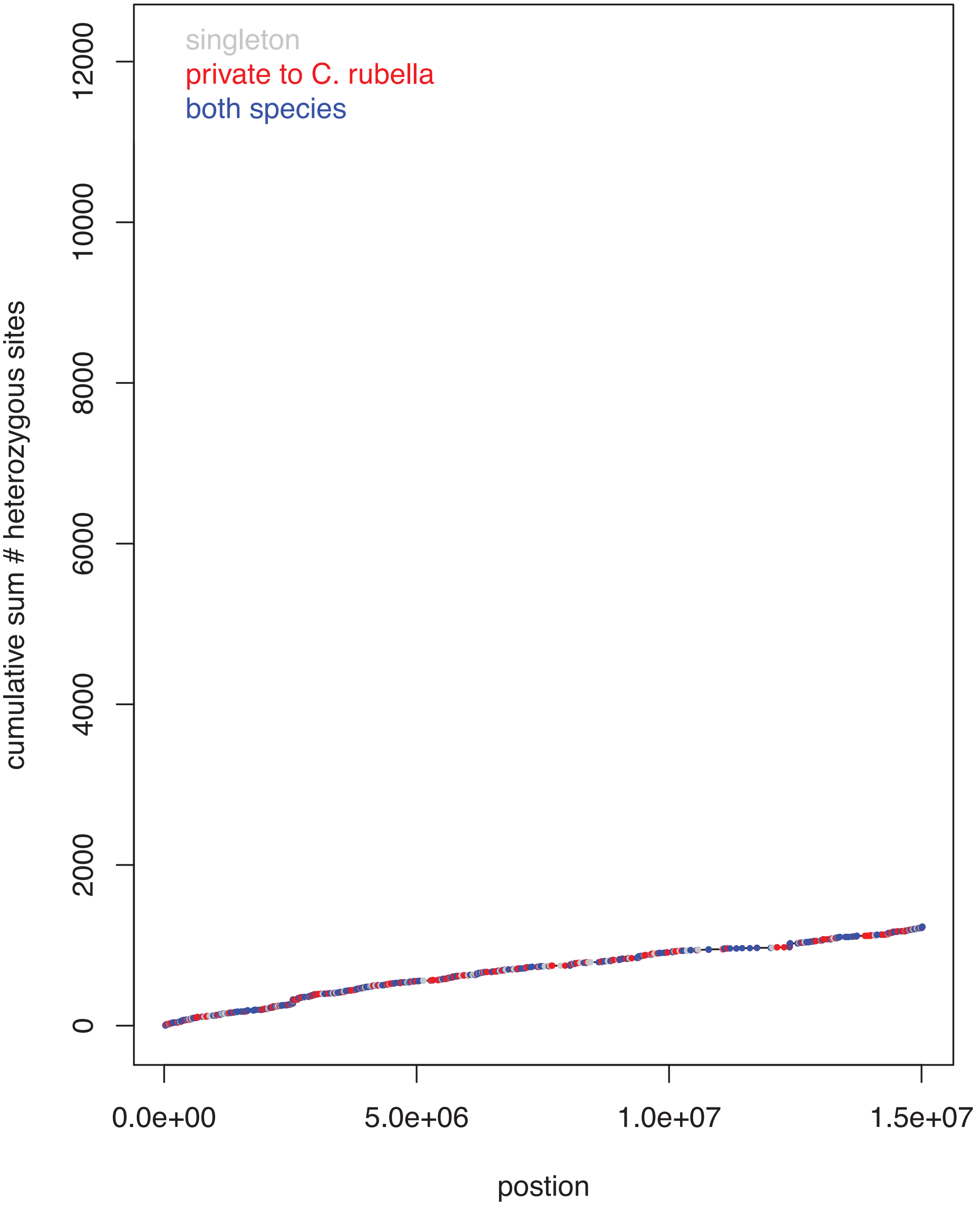

S9_C.4) **Cr34 (Italian), Chromosome 4**

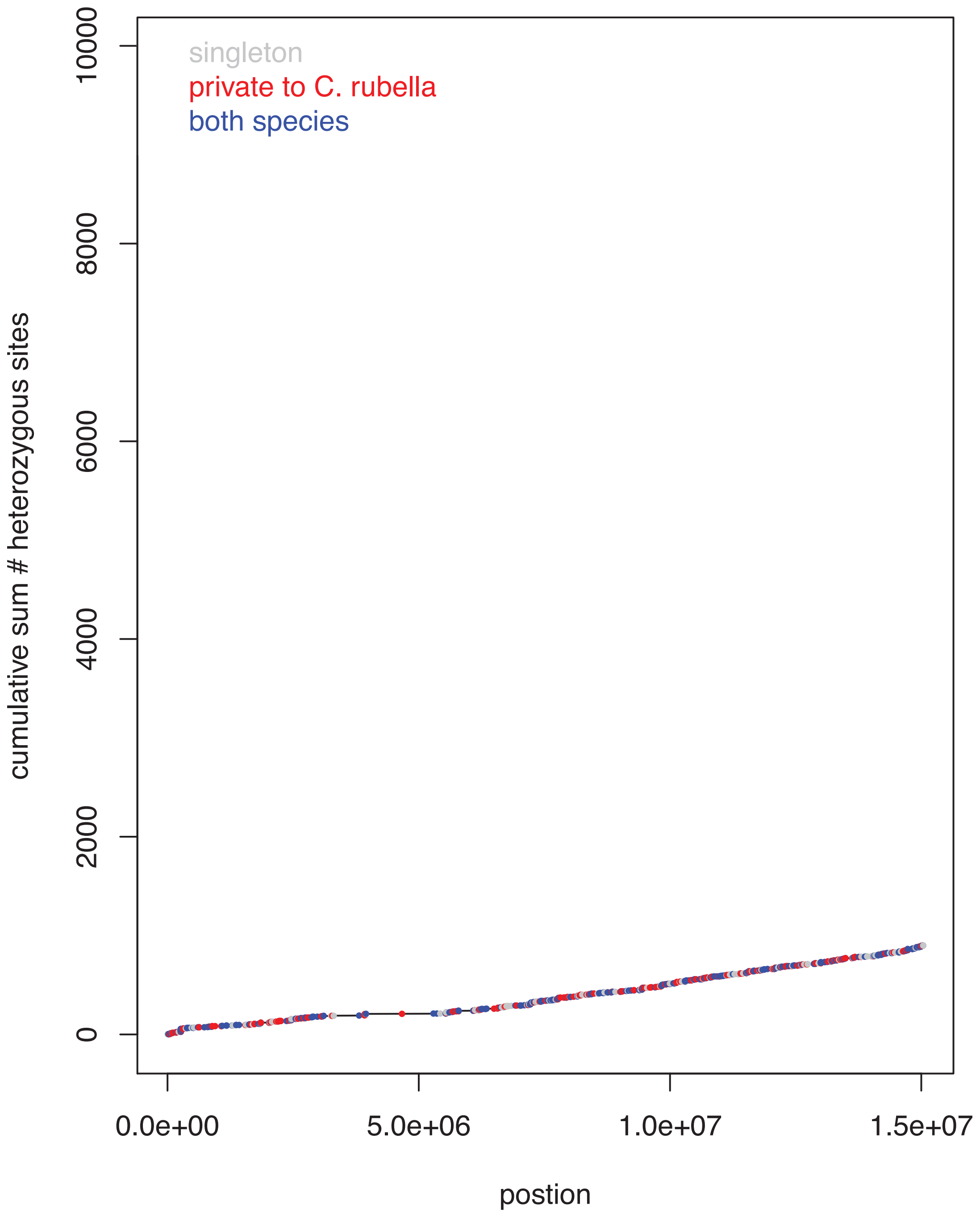

S9_C.5) **Cr34 (Italian), Chromosome 5**

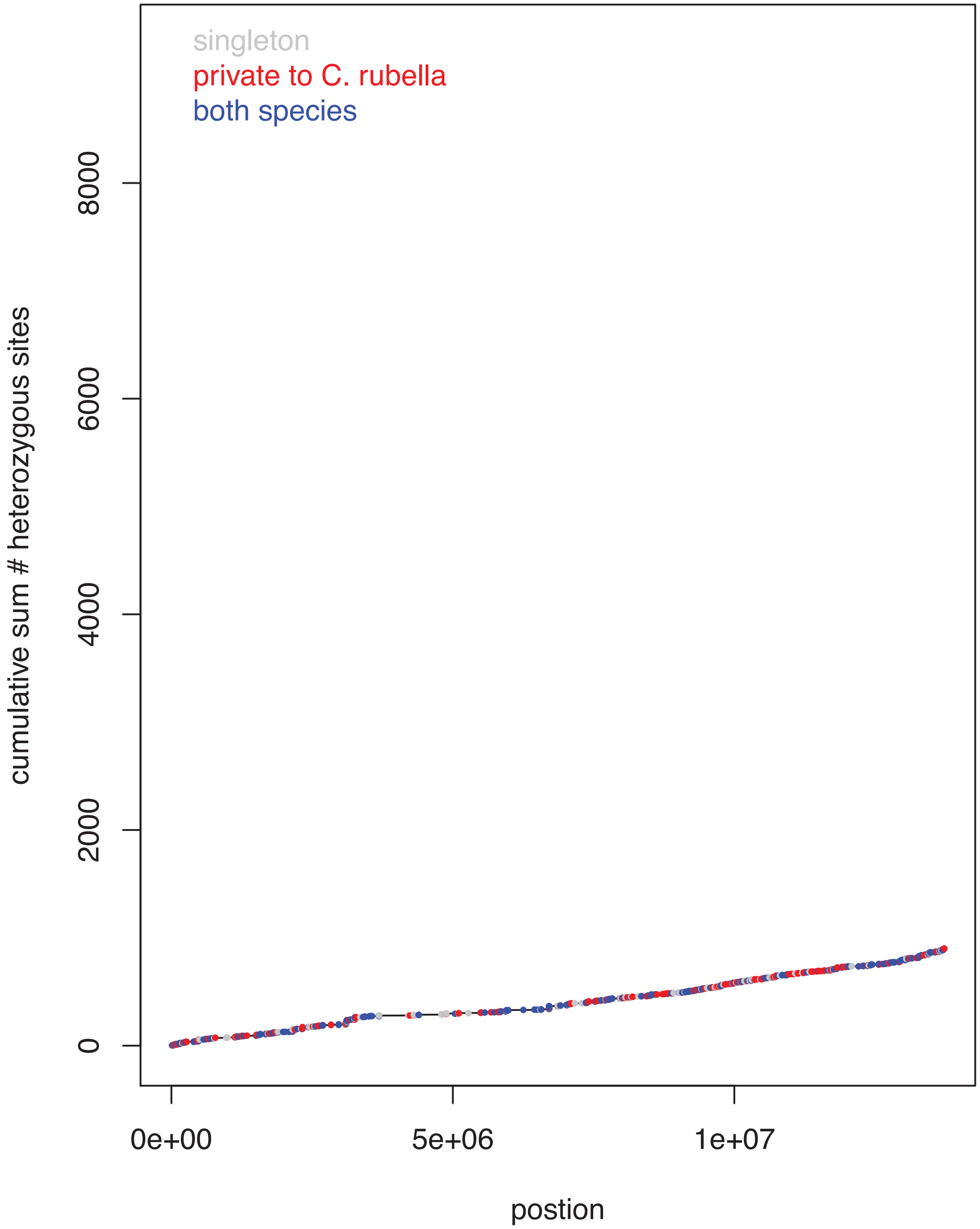

S9_C.6) **Cr34 (Italian), Chromosome 6**

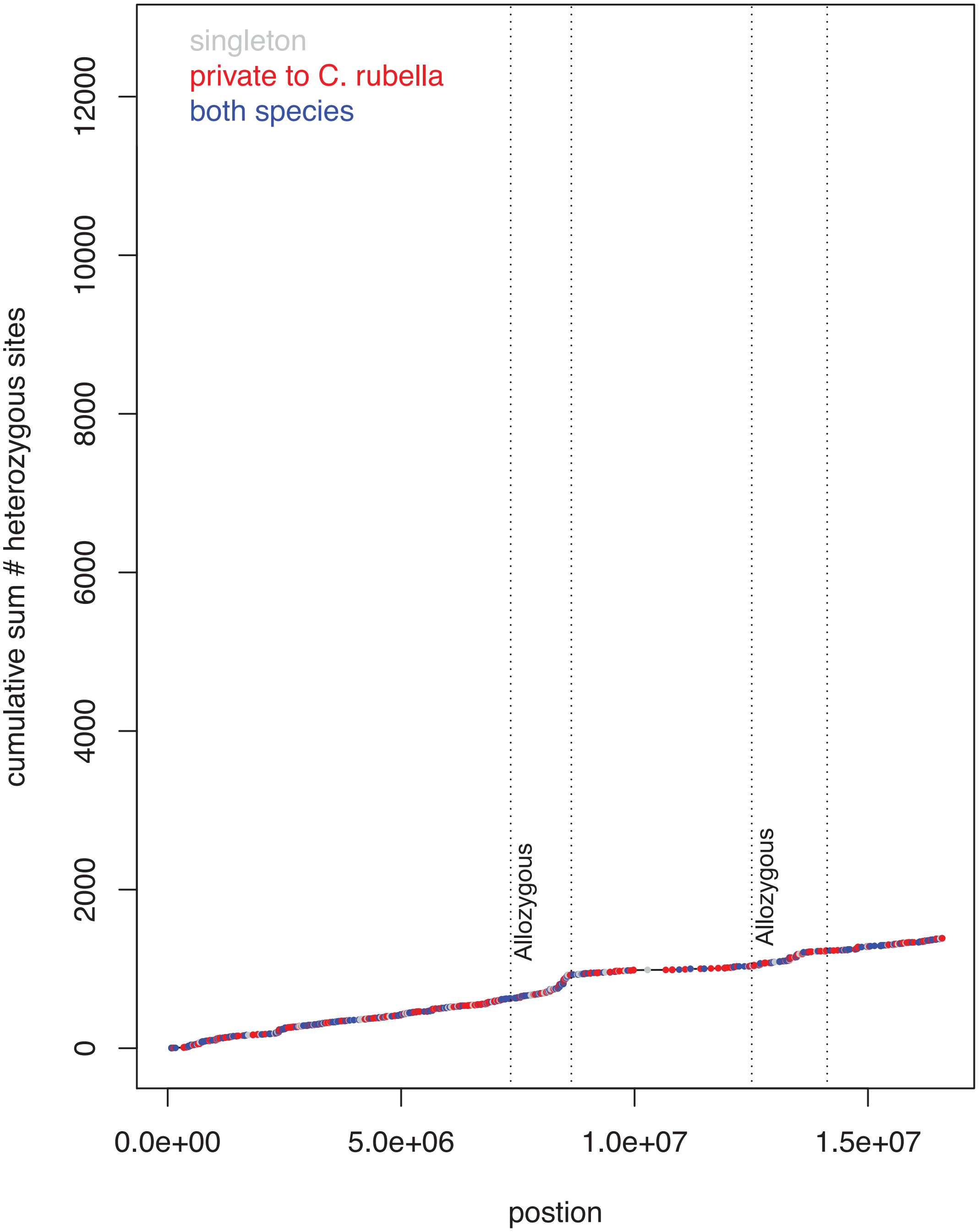

S9_C.7) **Cr34 (Italian), Chromosome 7**

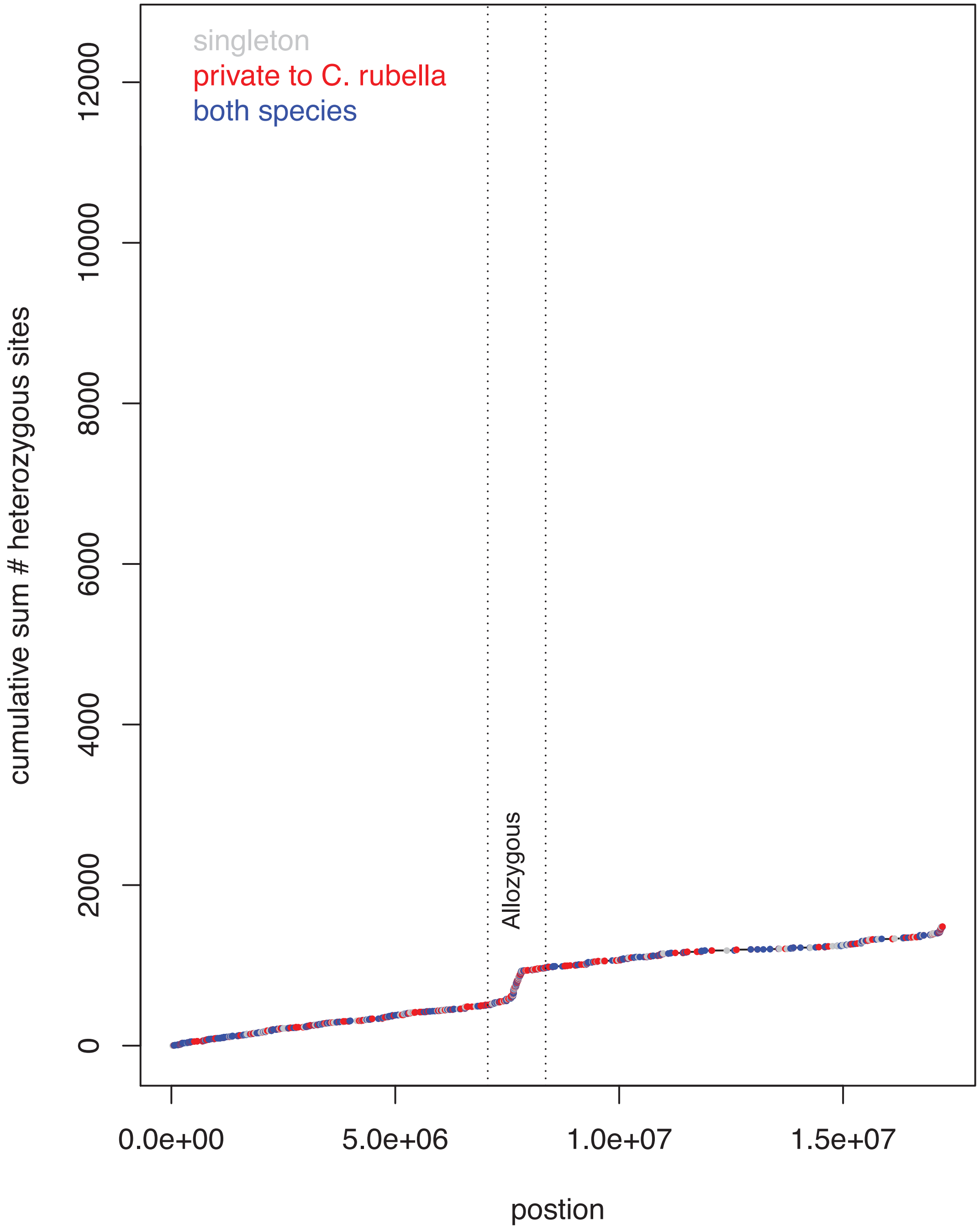

S9_C.8) **Cr34 (Italian), Chromosome 8**

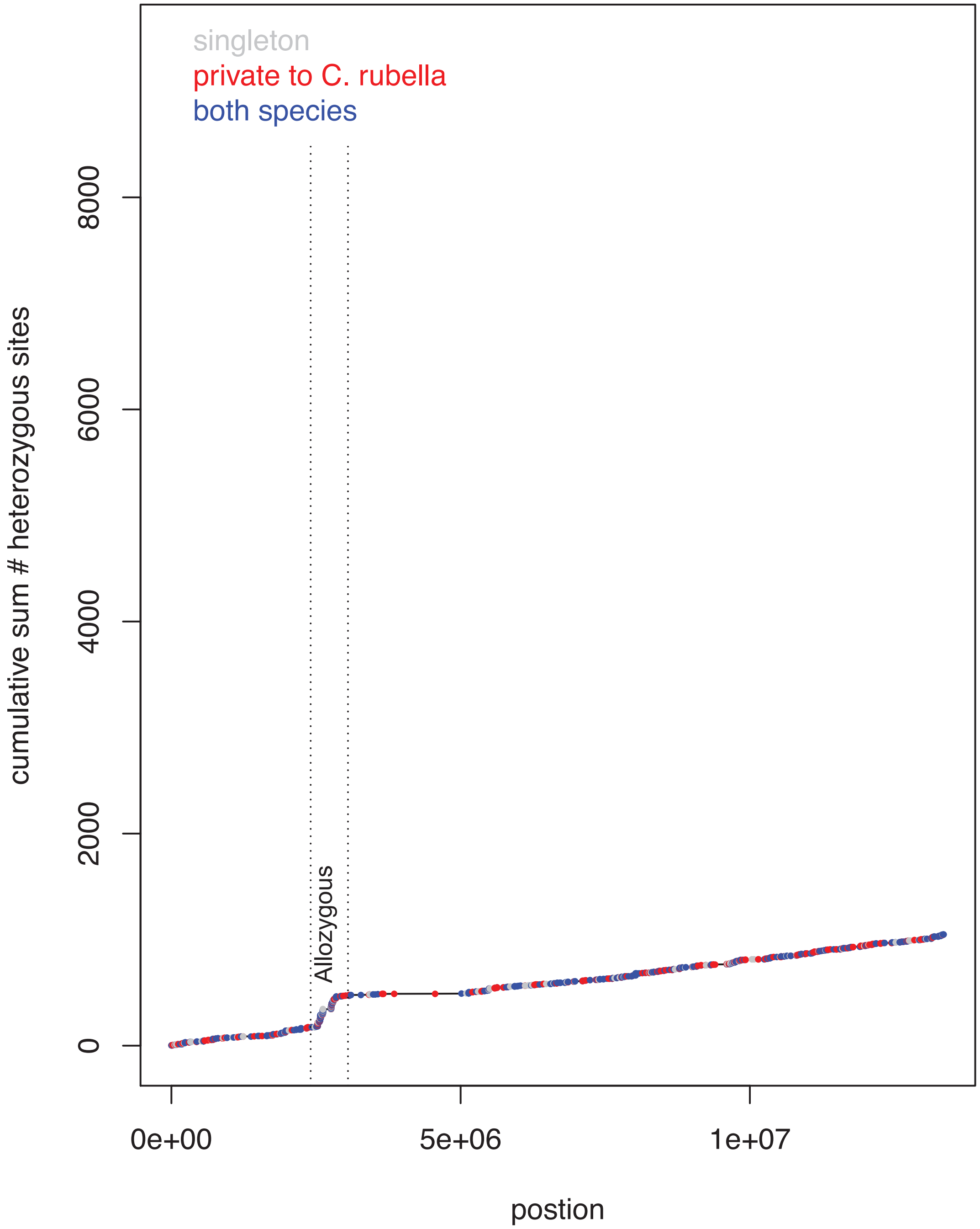

S9_D.1) Cr75 (Greek), Chromosome 1

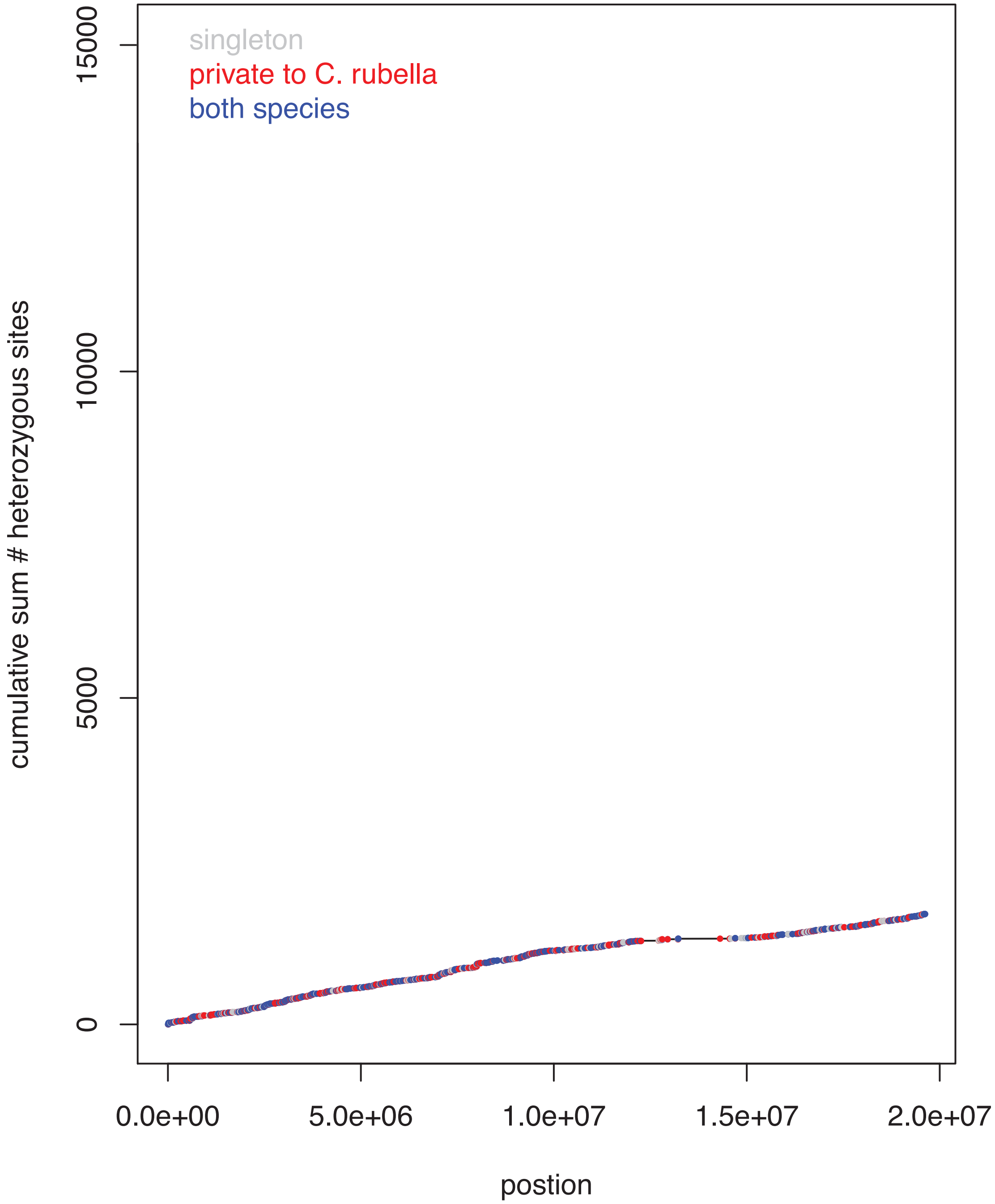

S9_D.2) **Cr75 (Greek), Chromosome 2**

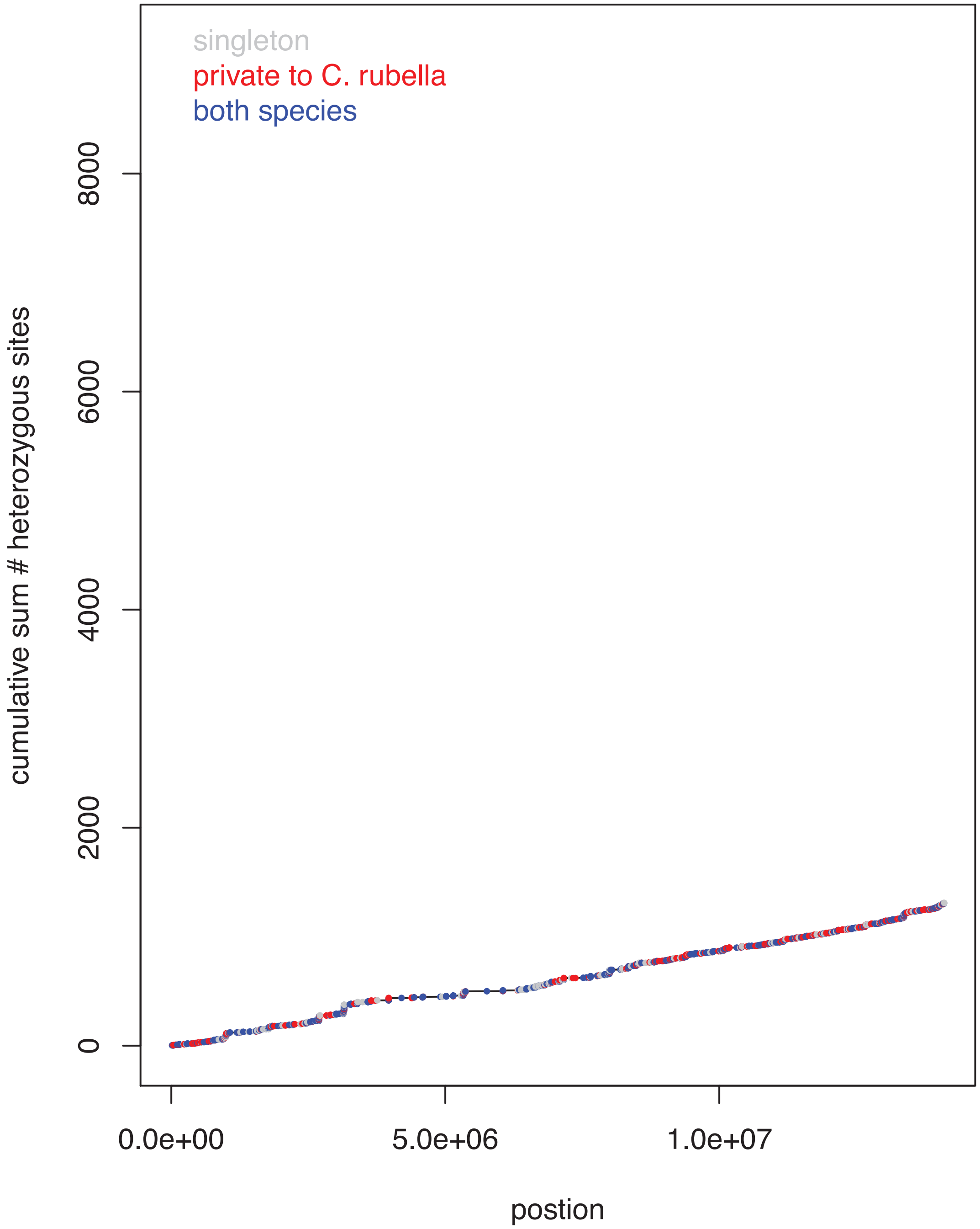

S9_D.3) **Cr75 (Greek), Chromosome 3**

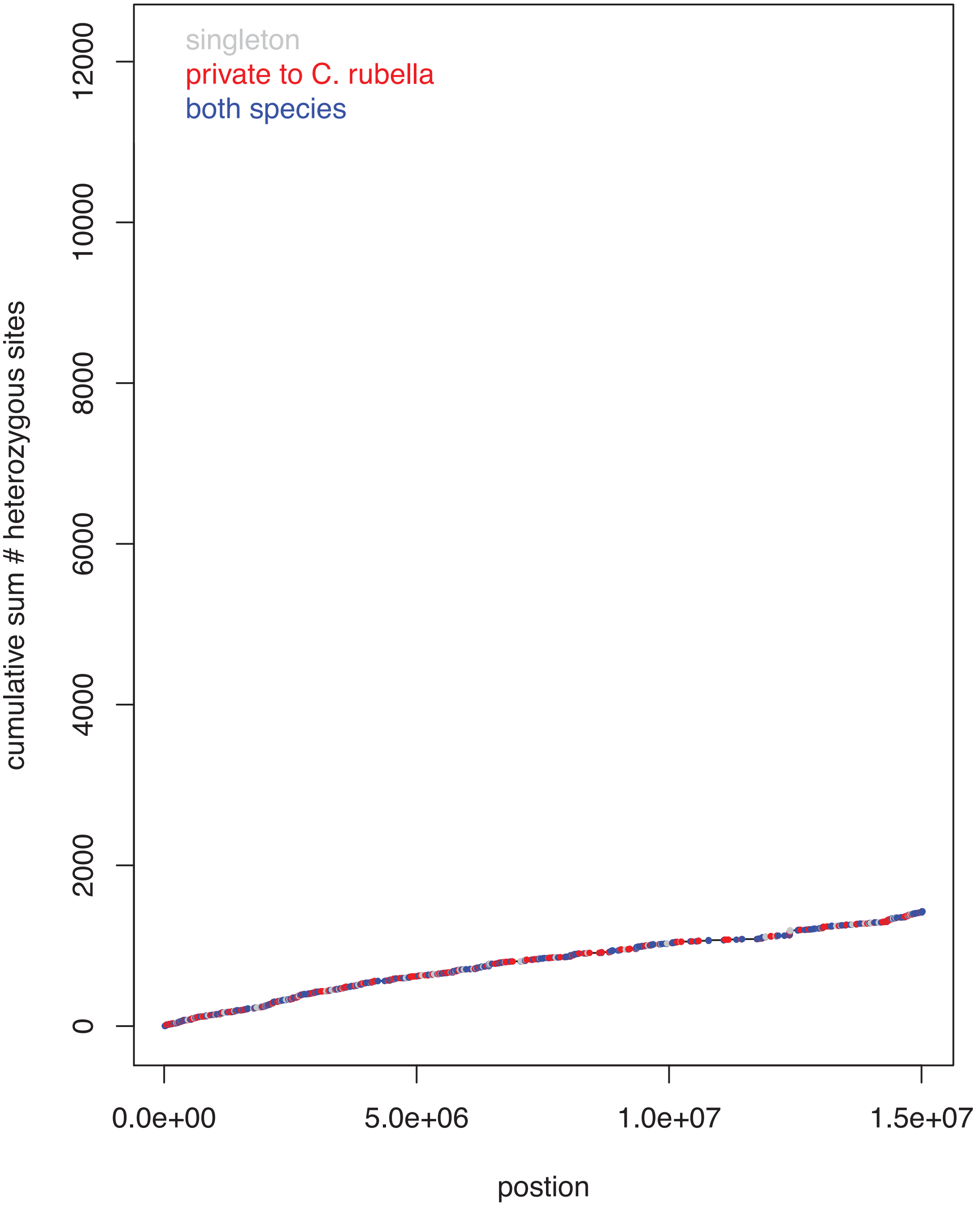

S9_D.4) Cr75 (Greek), Chromosome 4

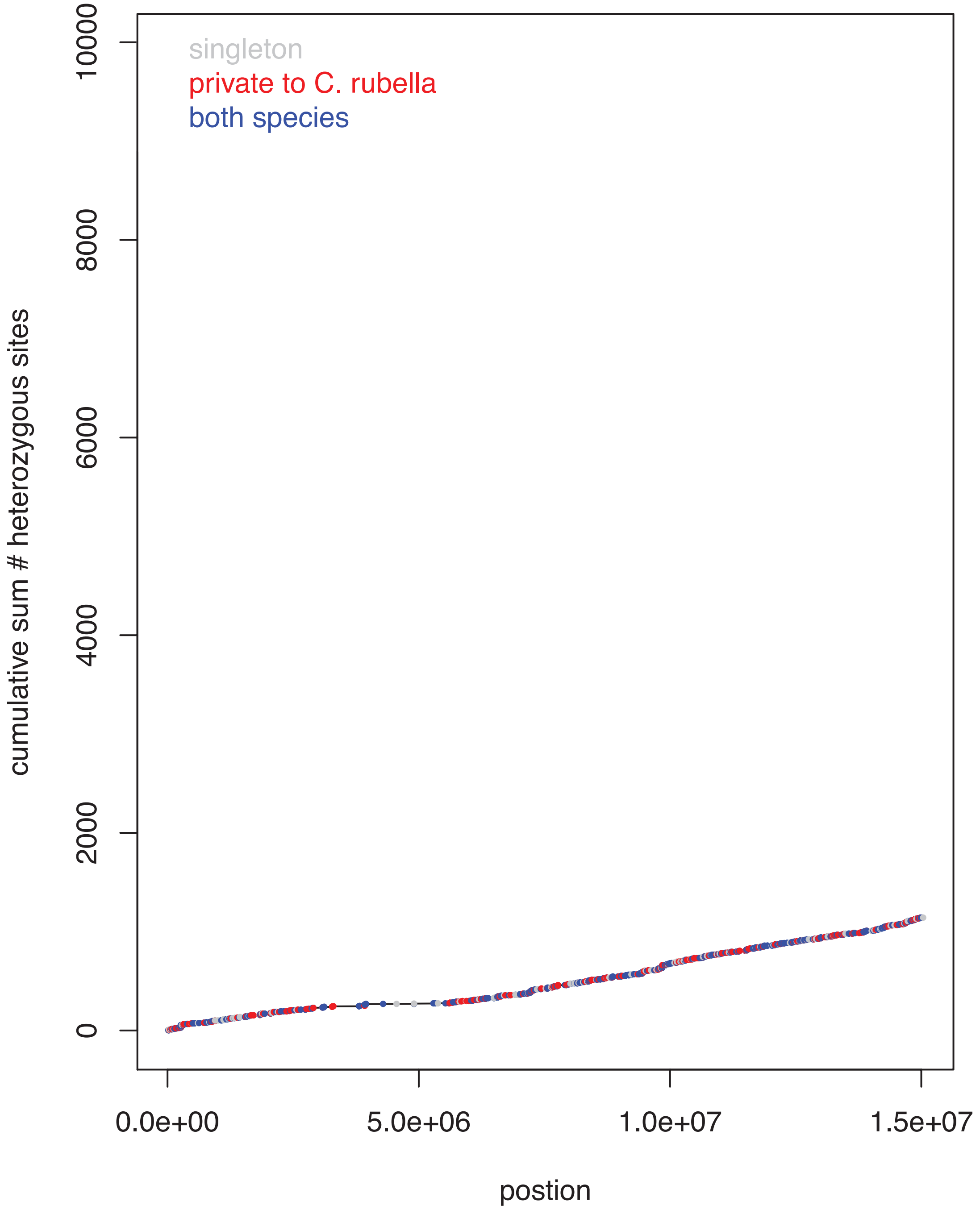

S9_D.5) **Cr75 (Greek), Chromosome 5**

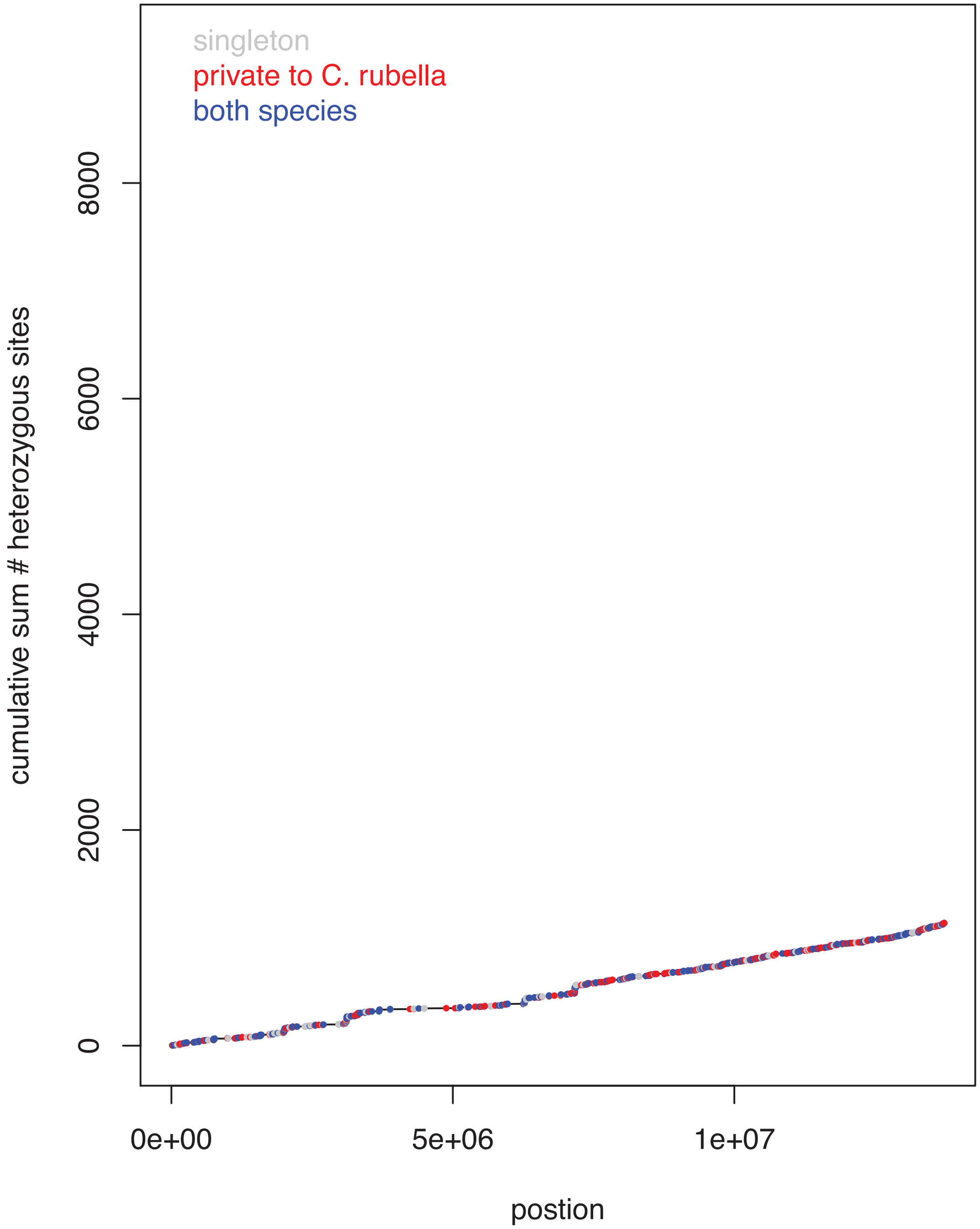

S9_D.6) **Cr75 (Greek), Chromosome 6**

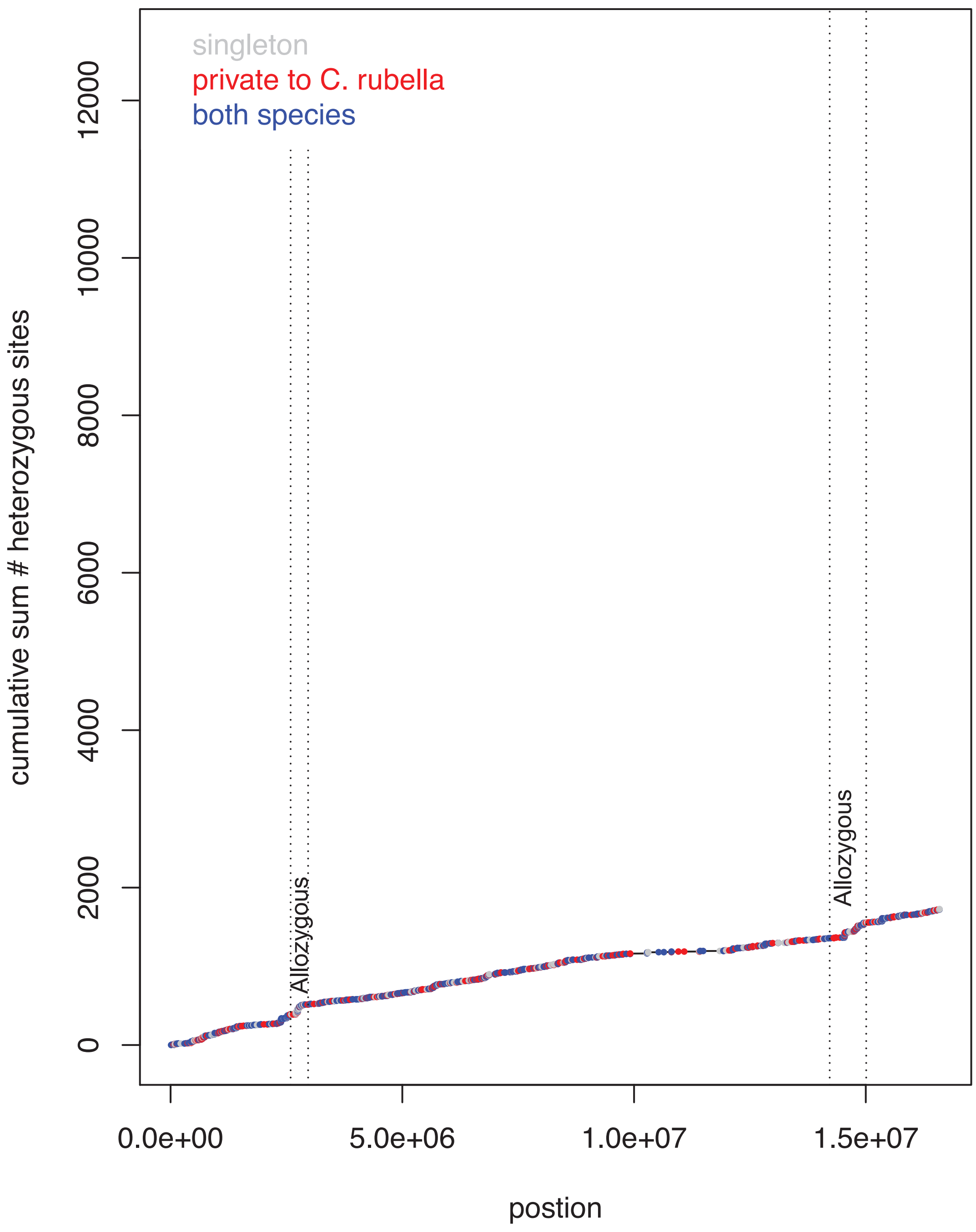

S9_D.7) **Cr75 (Greek), Chromosome 7**

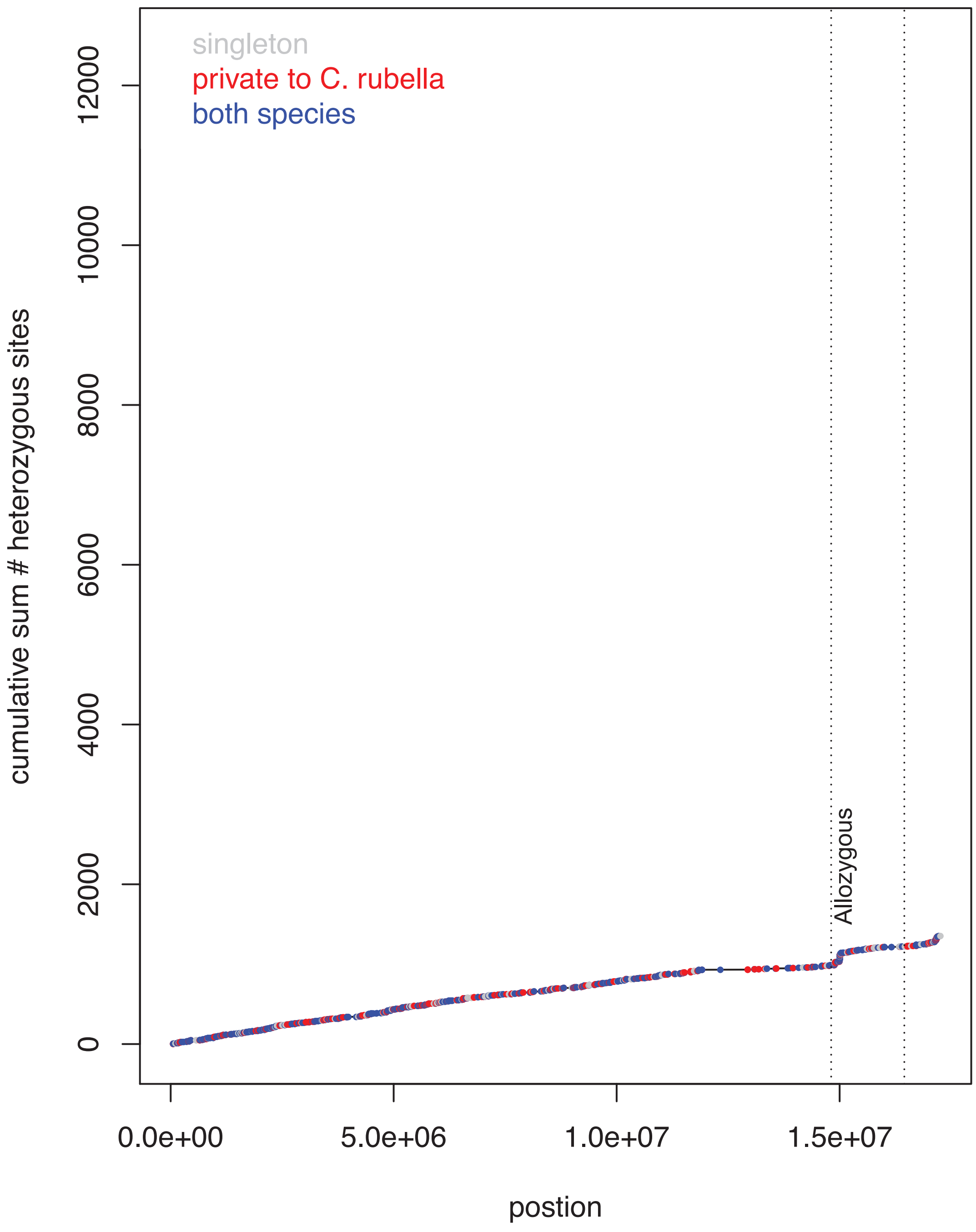

S9_D.8) **Cr75 (Greek), Chromosome 8**

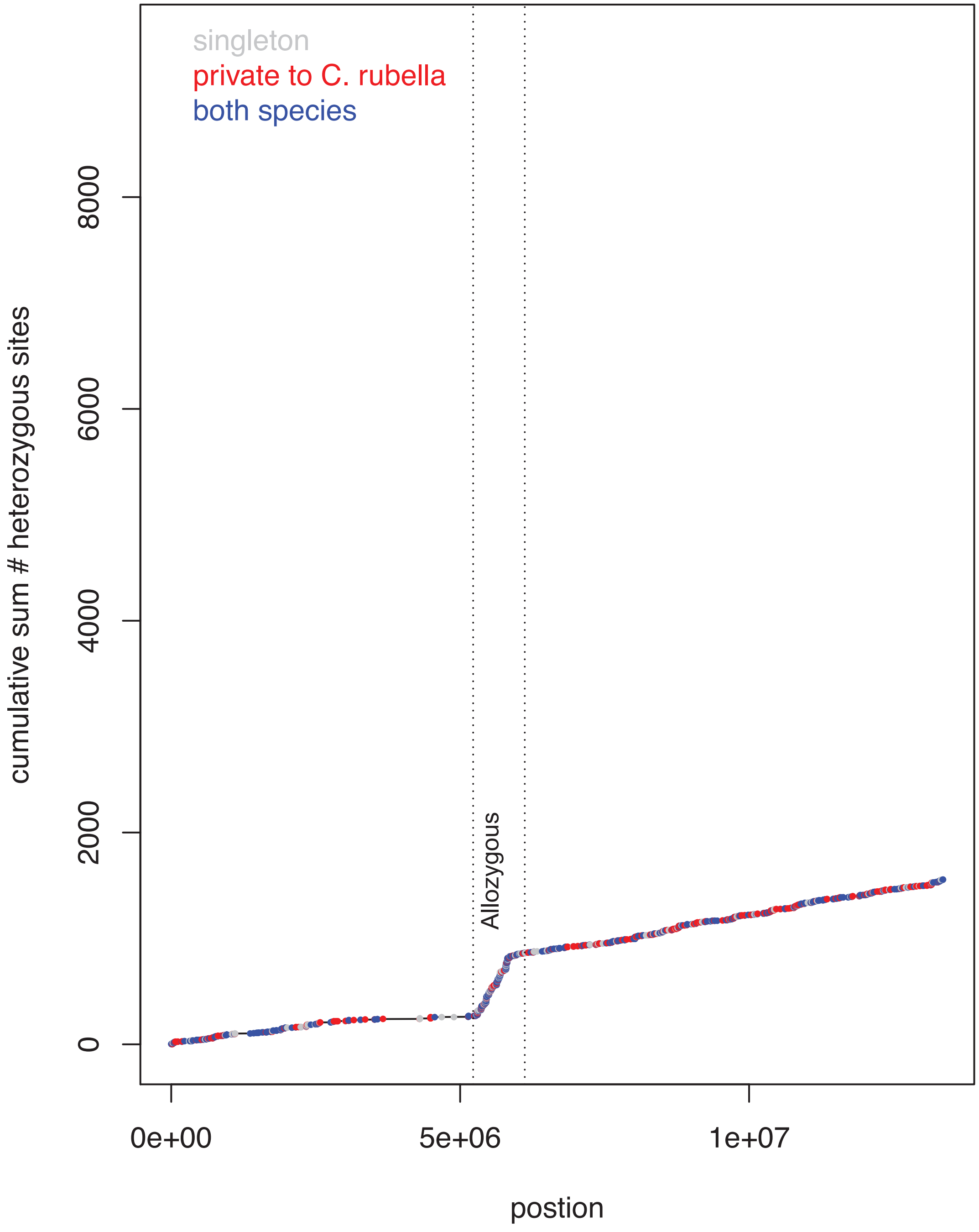

S9_E.1) Cr1337 (Argentinian), Chromosome 1

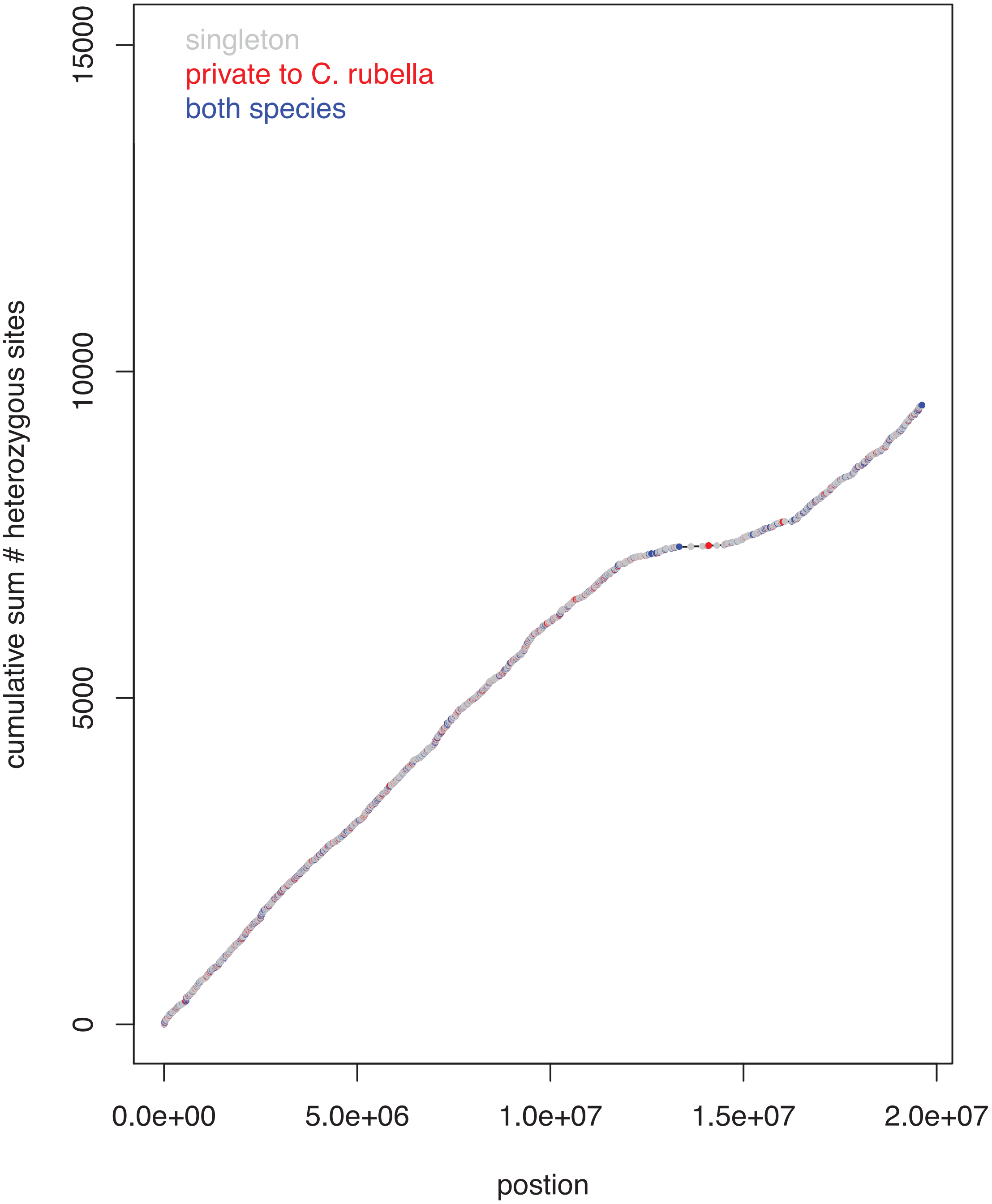

S9_E.2) Cr1337 (Argentinian), Chromosome 2

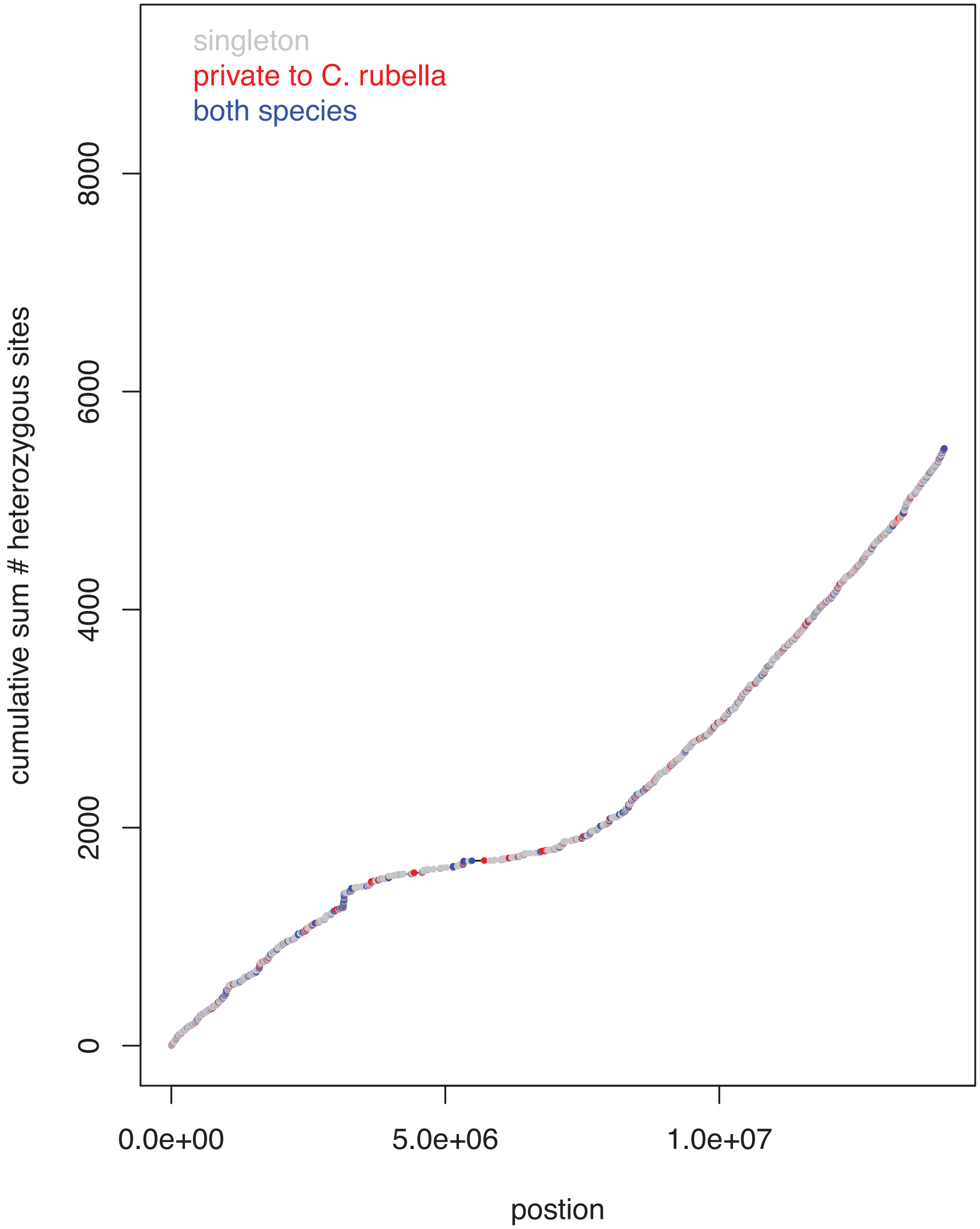

S9_E.3) Cr1337 (Argentinian), Chromosome 3

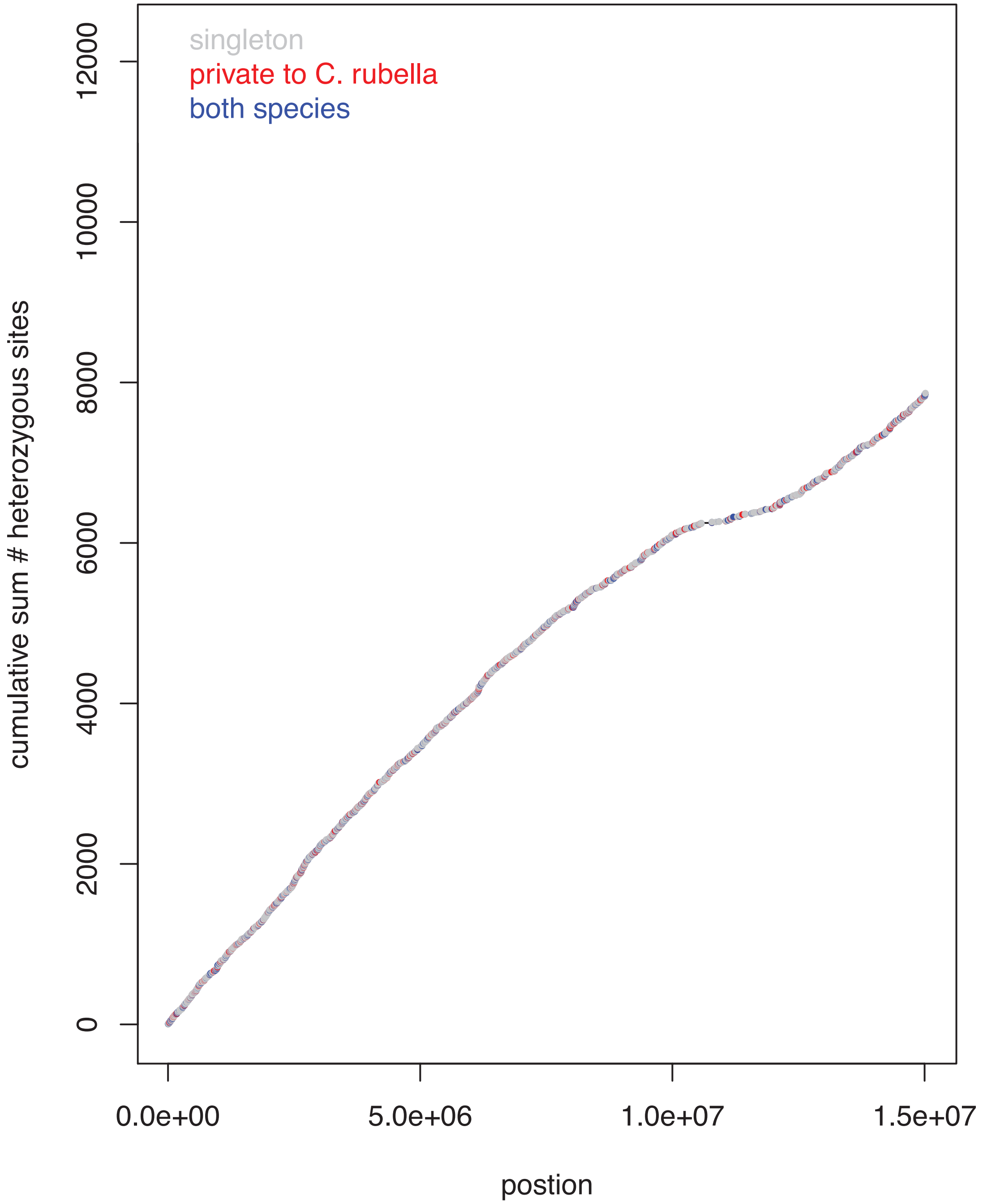

S9_E.4) Cr1337 (Argentinian), Chromosome 4

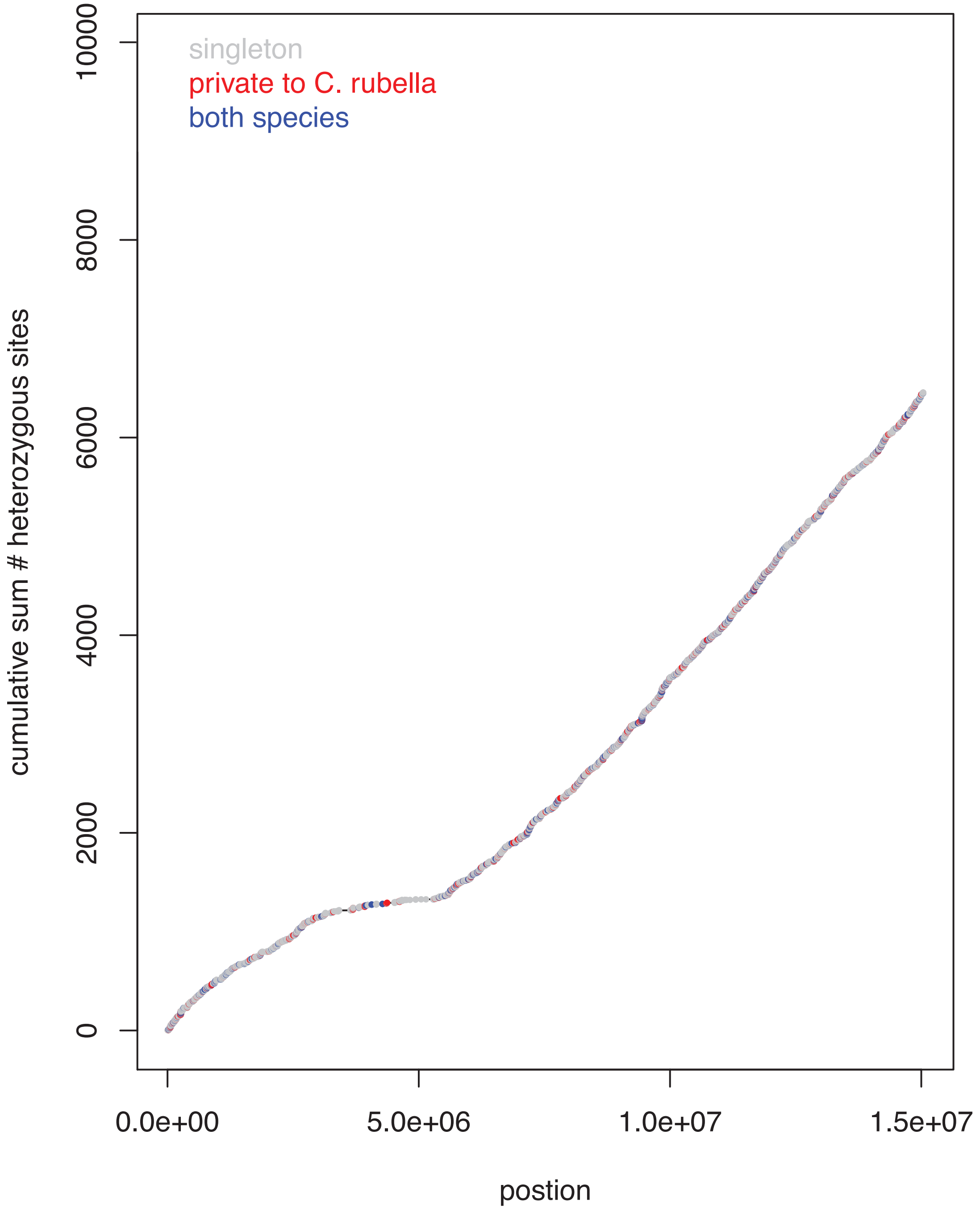

S9_E.5) Cr1337 (Argentinian), Chromosome 5

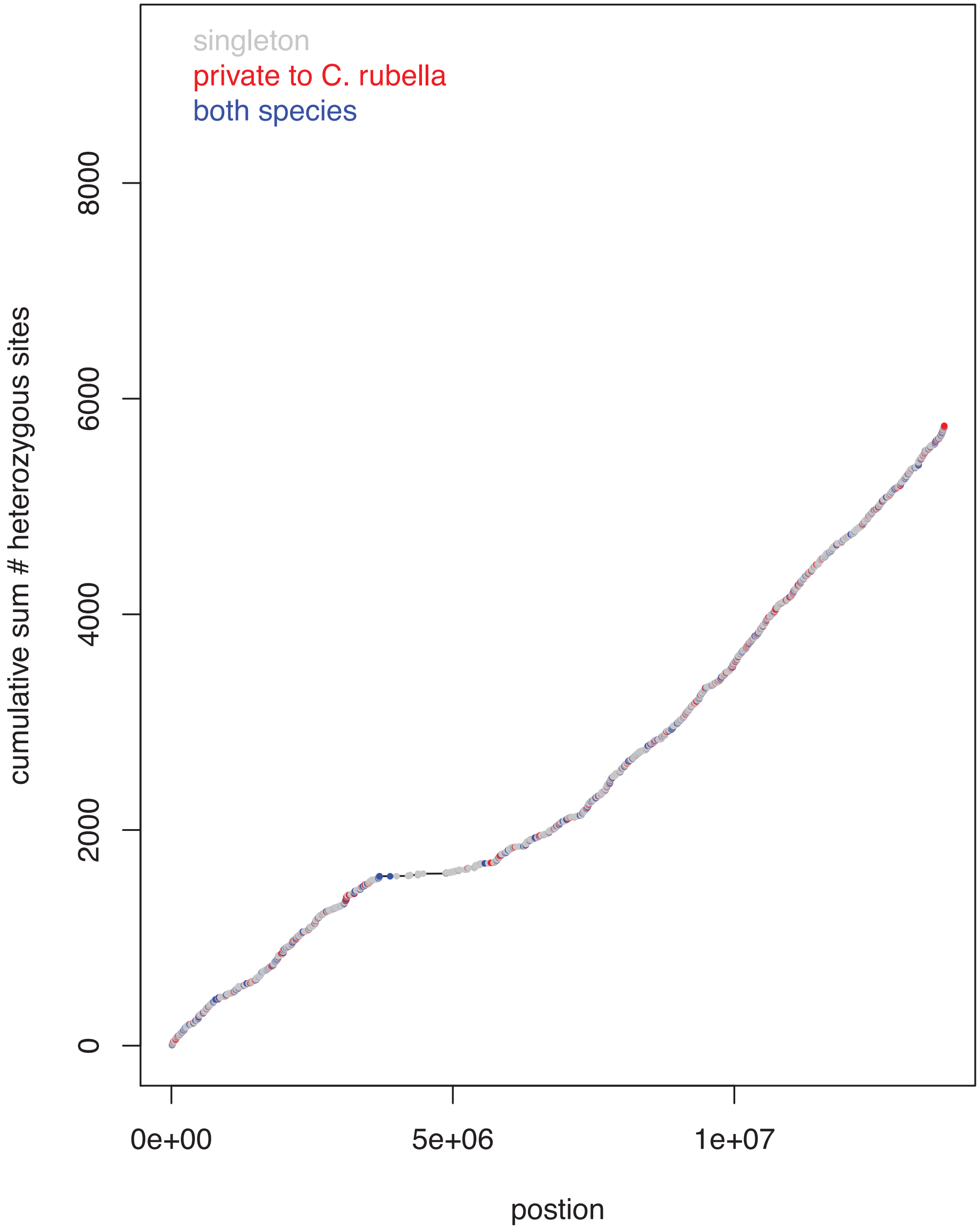

S9_E.6) **Cr1337 (Argentinian), Chromosome 6**

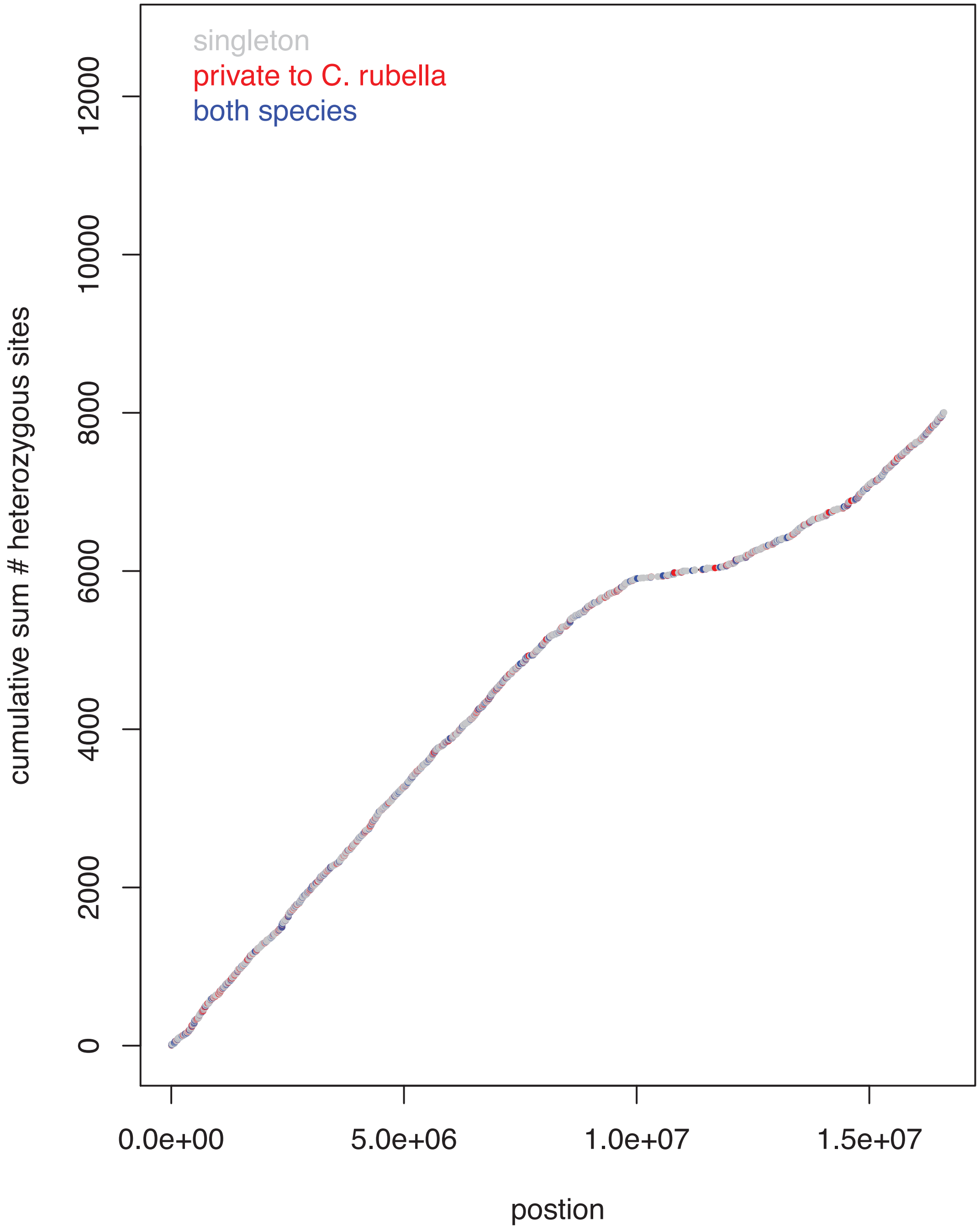

S9_E.7) **Cr1337 (Argentinian), Chromosome 7**

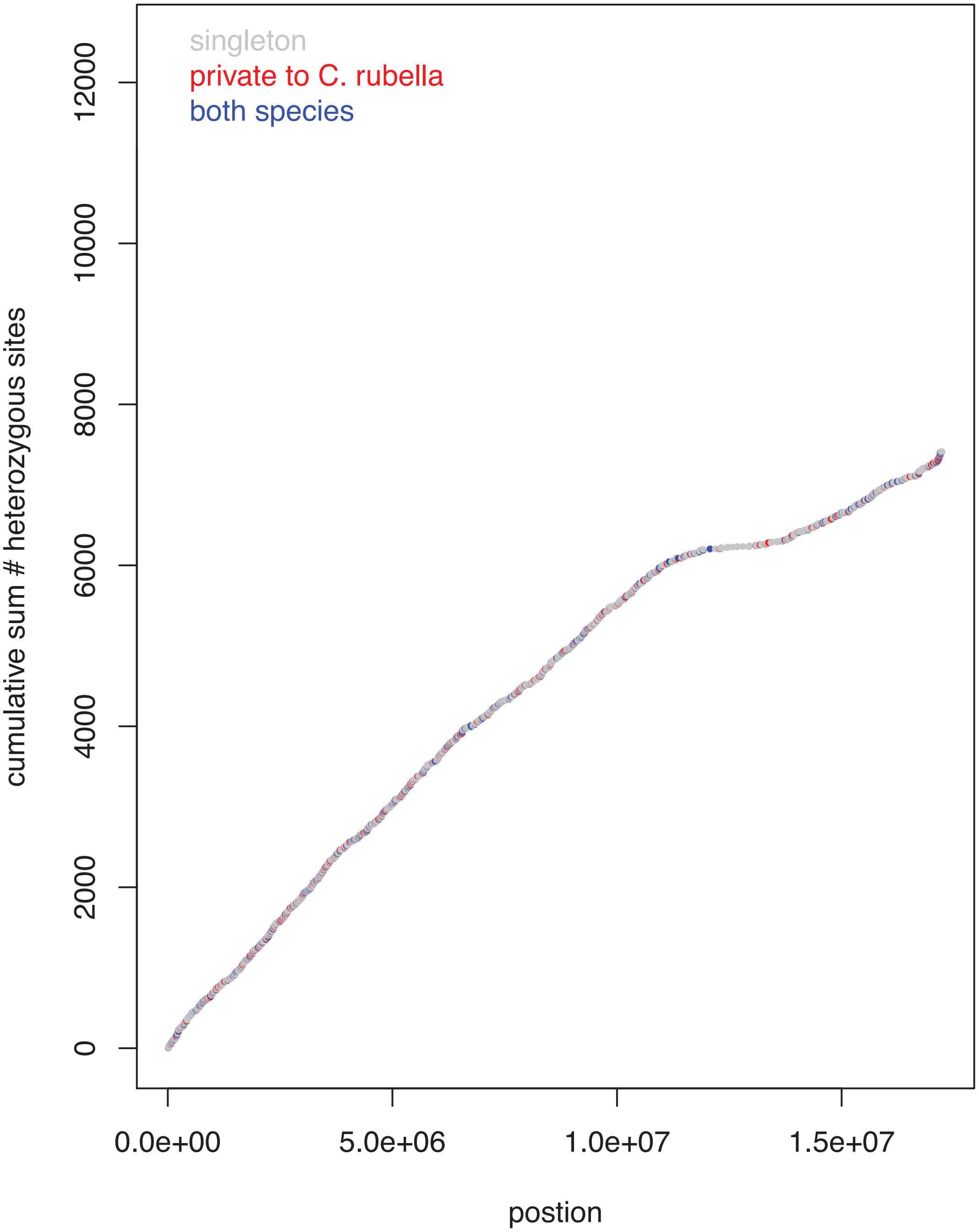

S9_E.8)   Cr1337 (Argentinian), Chromosome 8

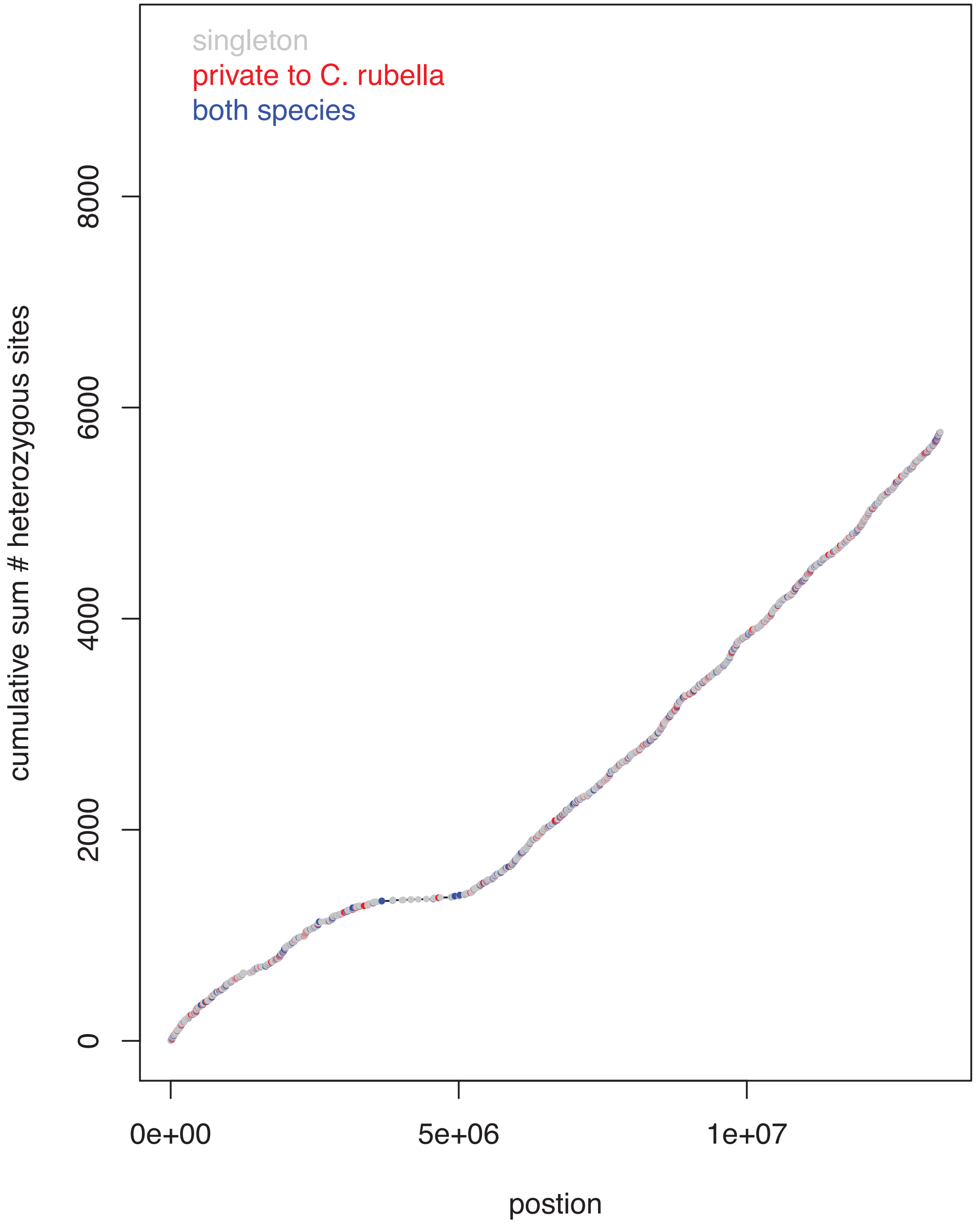

S9_F.1) Crtaal (Algerian), Chromosome 1

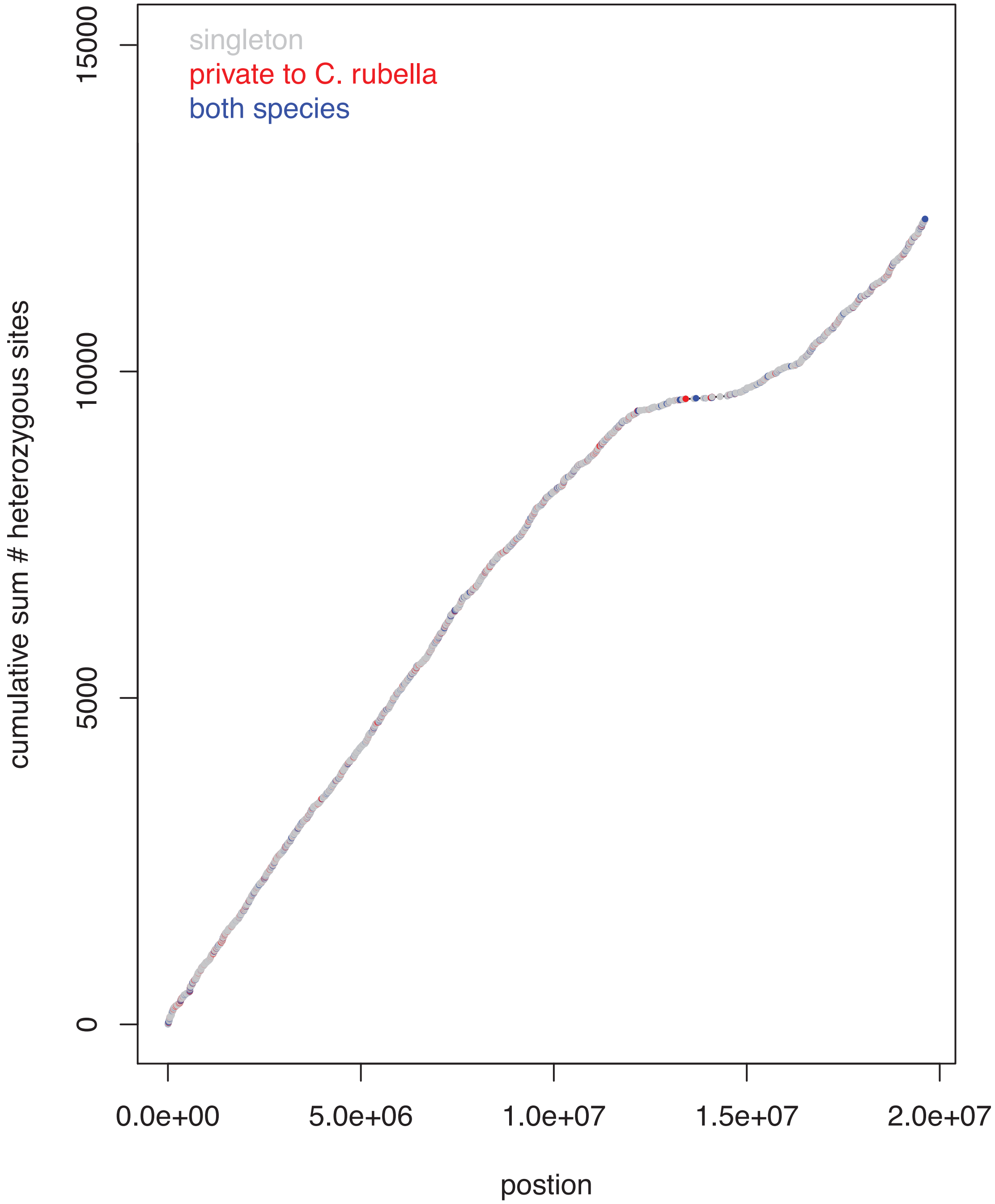

S9_F.2) **Crtaal (Algerian), Chromosome 2**

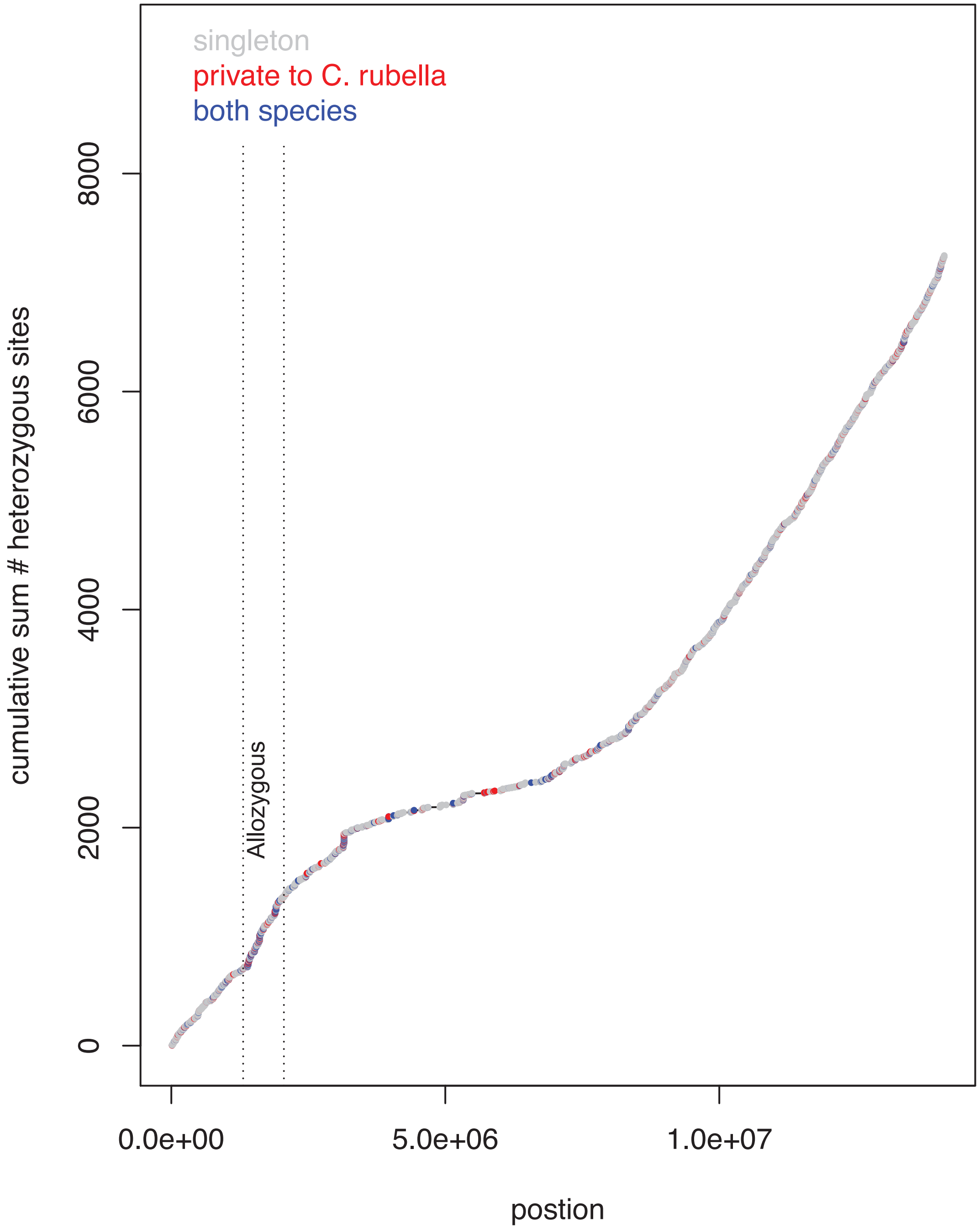

S9_F.3) Crtaal (Algerian), Chromosom

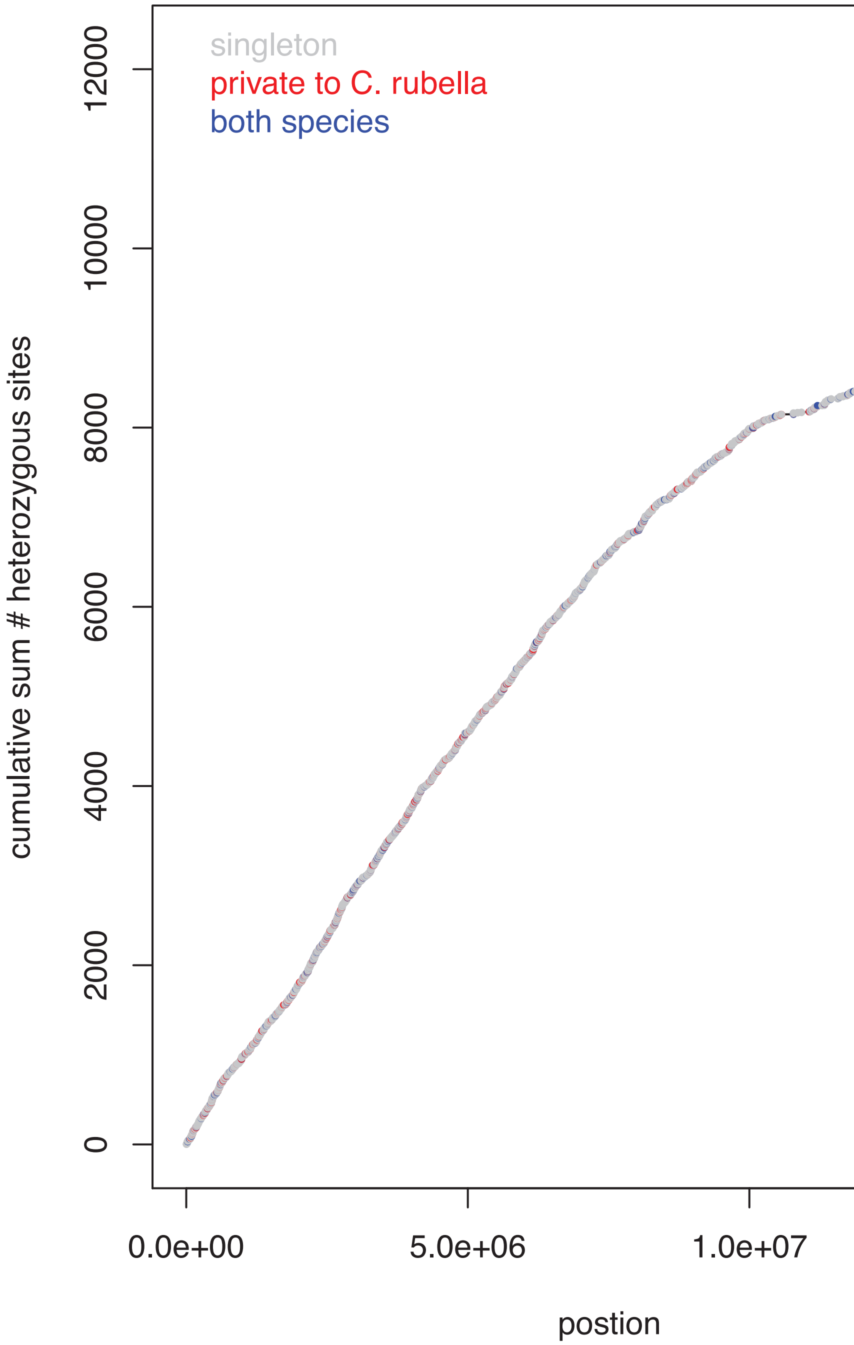

S9_F.4) **Crtaal (Algerian), Chromosome 4**

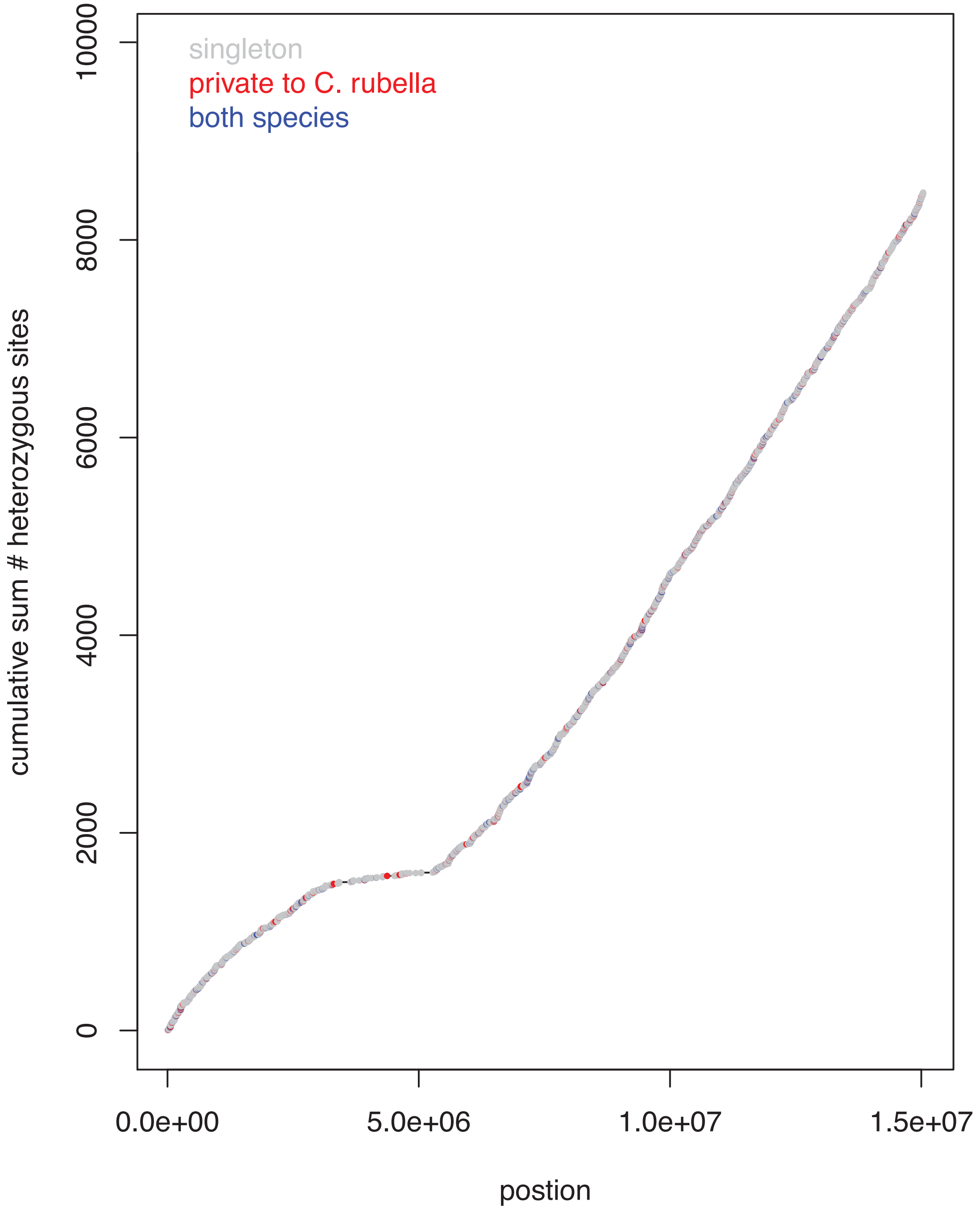

S9_F.5) Crtaal (Algerian), Chromosome 5

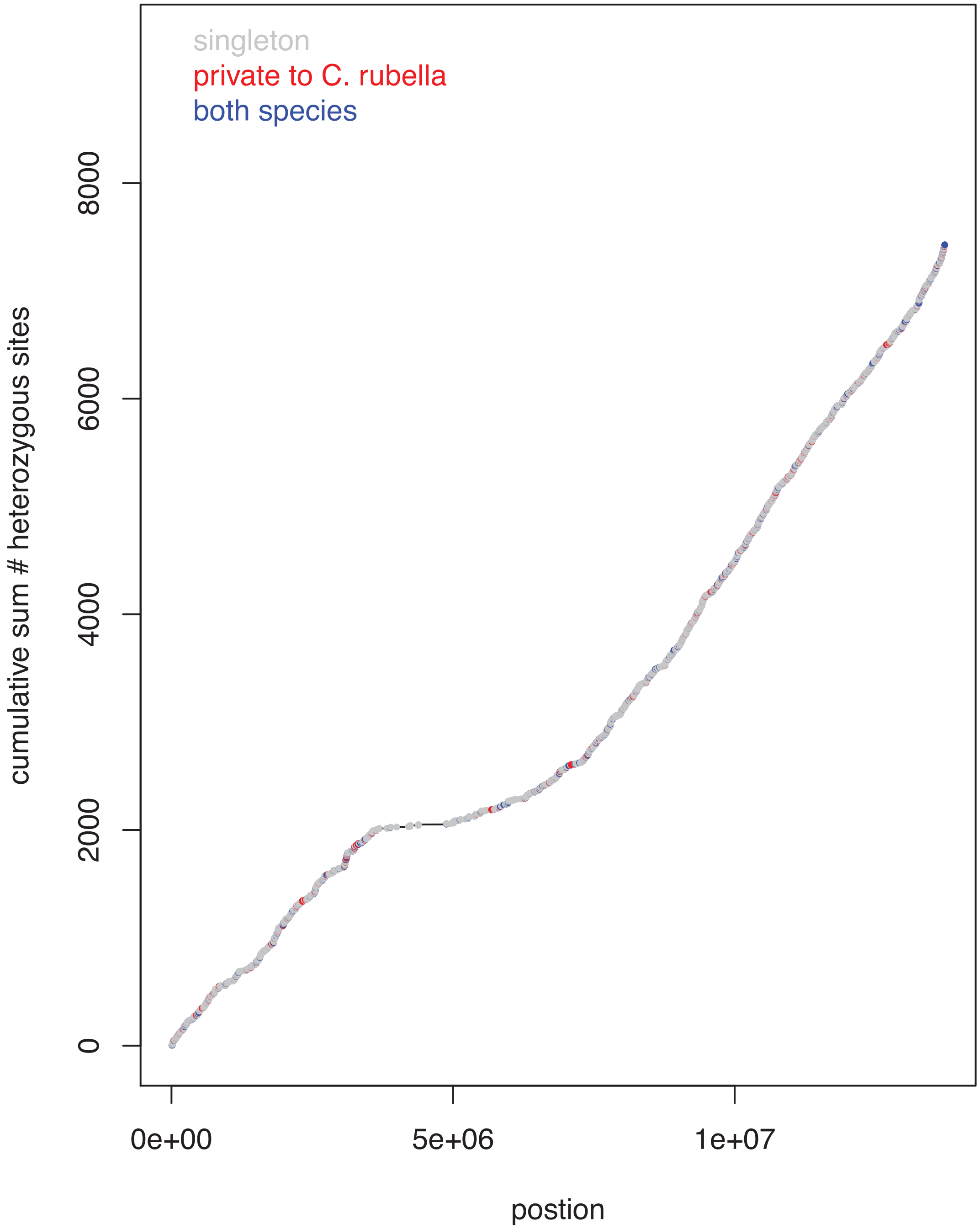

S9_F.6) Crtaal (Algerian), Chromosome 6

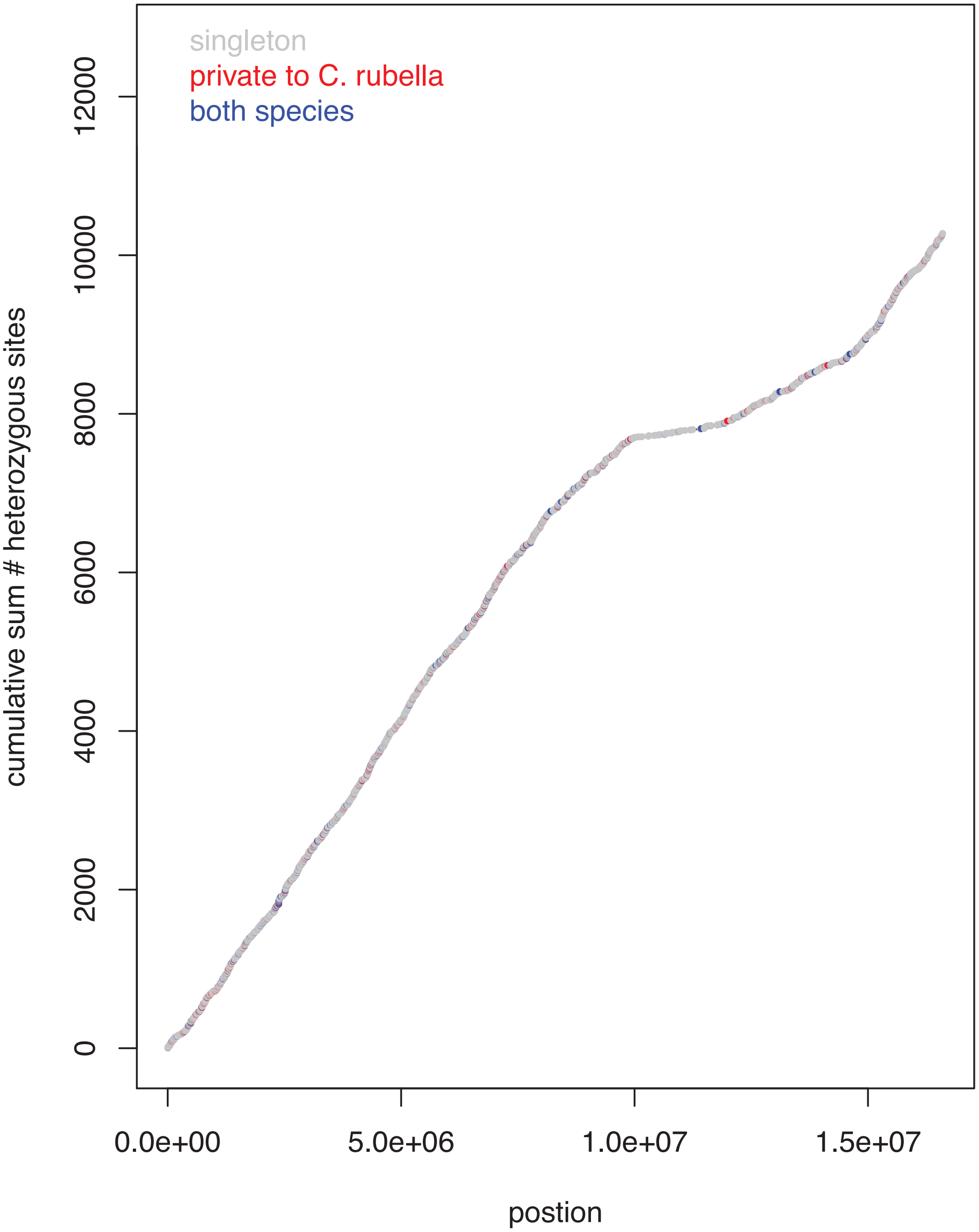

S9_F.7) Crtaal (Algerian), Chromosome 7

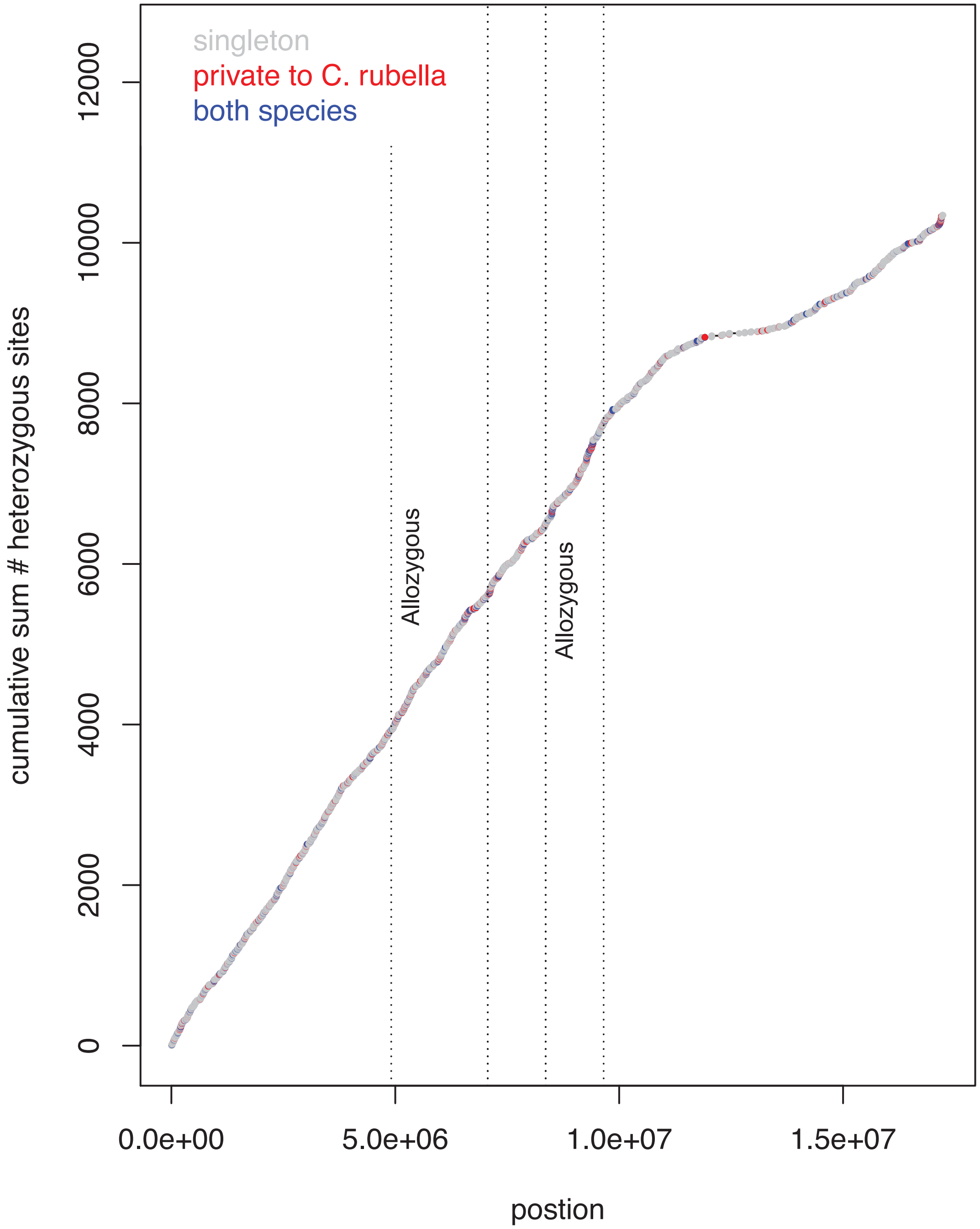

S9_F.8) **Crtaal (Algerian), Chromosome 8**

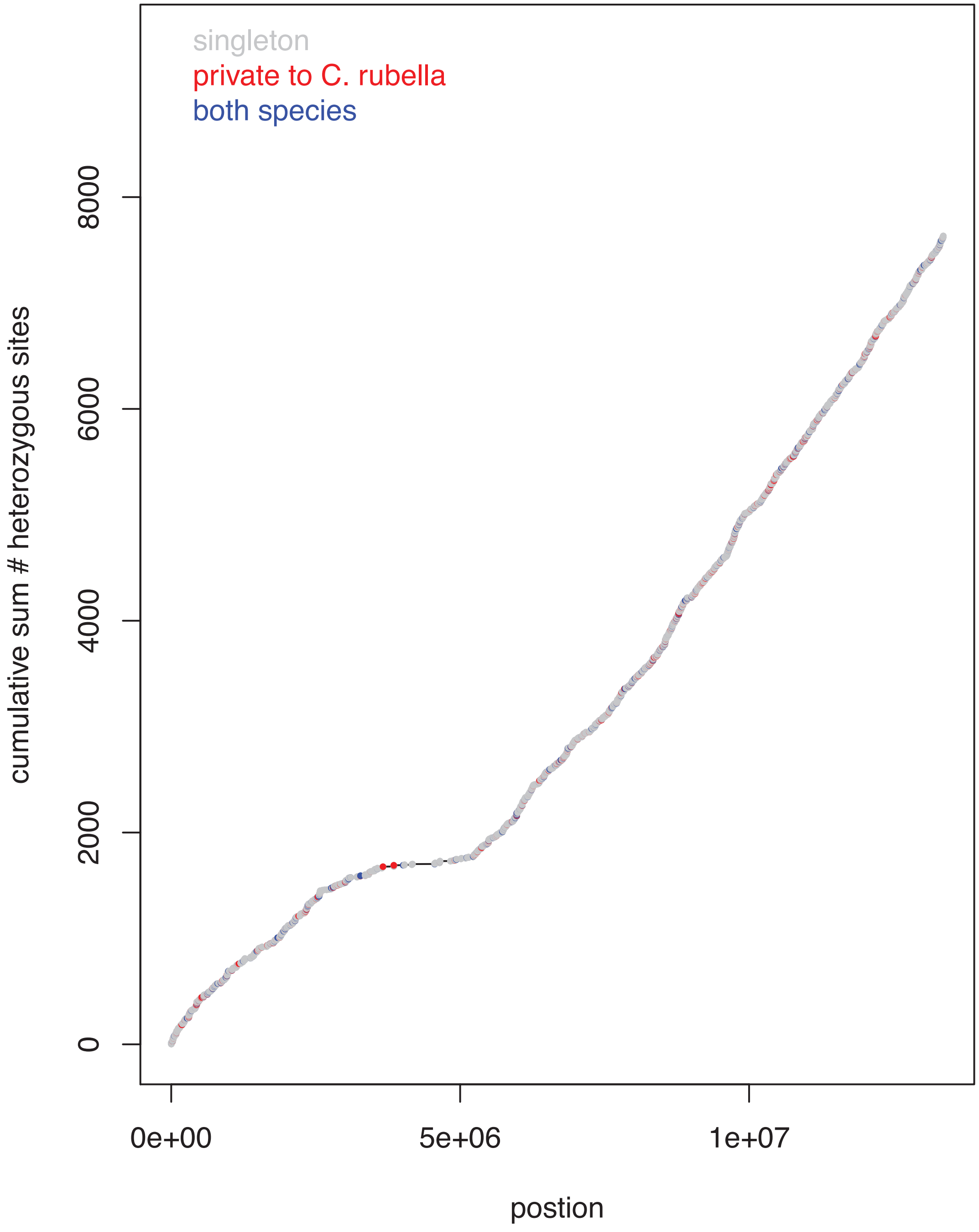

Supplement: Figure S9 — Genome-wide individual heterozygosity in C. rubella. We label allozygous regions by eye (Letters A–F represent individuals, and numbers 1–8 represent chromosomes – see figure titles). Blue and red points display sites heterozygous in C. rubella or both species, respectively, while singleton sites are presented in grey. Dotted lines separate regions inferred to be autozygous and allozygous. The cumulative number of heterozygous genotypes is plotted on the y-axis, and the physical position is displayed on the x-axis. We infer a region to be allozygous when this slope is relatively large. (PDF) [file pgen.1003754.s009.pdf]
