# Supplementary material for: Genomic Identification of Founding Haplotypes Reveals the History of the Selfing Species Capsella rubella
Source: PLoS Genet. 2013 Sep 12;9(9):e1003754. doi: 10.1371/journal.pgen.1003754 (PMC3772084; doi:10.1371/journal.pgen.1003754)
Supplement: Table S1 — Validation of C. rubella genotype calls by Sanger sequencing. Genomic regions targeted for Sanger sequencing. The chromosome, genomic location, orientation of each ORF, and the individuals sequenced is noted in each column. Concordance between Sanger and RNA-seq genotypes calls, split by individual. Numbers refer to the number of inferred non-reference alleles (e.g. 0 = homozygous for the reference, 1 = heterozygous, 2 = homozygous non-reference), and NAs mark missing data or data that did not pass QC. Sanger genotypes are presented before, and RNA-Seq genotypes are presented after the ‘/’. Note the minimal discordance between genotype calls by technology. (PDF) [file pgen.1003754.s011.pdf]

Table S1A: Sanger sequencing efforts.

chrom	start	stop	orientation	inds
1	1121520	1122379	revcomp	Cr1Gr1, Cr75, Cr81, Crtaal
1	2189244	2189862	revcomp	Cr1Gr1, Cr75, Cr81, Crtaal
1	3187217	3187782	revcomp	Cr75, Cr81, Crtaal
1	9252546	9253264	forward	Cr1337, Cr1Gr1, Cr81, Crtaal
1	9791410	9792193	revcomp	Cr1Gr1, Cr75, Cr81, Crtaal
1	12972008	12972691	forward	Cr1Gr1, Cr81, Crtaal
1	16978816	16979446	forward	Cr1Gr1, Crtaal
2	7158433	7159032	revcomp	Cr1Gr1, Cr75, Cr81, Crtaal
2	9111928	9112538	revcomp	Cr75, Crtaal
2	11501659	11502346	revcomp	Cr1Gr1, Cr75, Cr81, Crtaal
2	13499343	13499957	forward	Cr81
3	4380447	4381090	revcomp	Cr81
3	12783435	12784119	revcomp	Cr1Gr1, Cr75, Cr81, Crtaal
7	2781533	2782194	forward	Cr1Gr1, Cr75, Cr81, Crtaal
7	3433282	3434023	revcomp	Cr1Gr1, Cr81, Crtaal
7	3458572	3459198	revcomp	Cr1Gr1, Cr75, Cr81, Crtaal
7	4820394	4821059	forward	Cr1337, Cr1Gr1, Cr81, Crtaal
7	6079504	6080090	forward	Cr1337, Cr1Gr1, Cr75, Cr81, Crtaal
7	9123830	9124515	forward	Cr1Gr1, Cr75, Cr81
7	9214127	9214749	forward	Cr1Gr1, Cr75, Cr81, Crtaal
8	2578408	2579001	revcomp	Cr1Gr1, Cr75, Cr81, Crtaal
8	6671378	6671912	revcomp	Cr1Gr1, Cr75, Cr81, Crtaal
8	7784867	7785350	forward	Cr1Gr1, Crtaal, Cr1337, Cr75
8	9670696	9671200	forward	Cr1Gr1, Cr75, Cr81, Crtaal

Table S1B: Comparison of genotype calls across technology (Sanger/RNA-Seq).

Sample	0/0	2/2	0/NA	NA/0	2/NA	0/2	1/1	NA/NA
Cr1Gr1	12808	25	6	92	0	0	0	50
Cr75	10611	19	5	53	0	0	0	28
Cr81	13494	6	37	104	0	0	1	50
Crtaal	12904	8	369	139	2	2	0	50
Cr1337	2429	0	12	0	0	0	0	13
