# Supplementary material for: Genomic Identification of Founding Haplotypes Reveals the History of the Selfing Species Capsella rubella
Source: PLoS Genet. 2013 Sep 12;9(9):e1003754. doi: 10.1371/journal.pgen.1003754 (PMC3772084; doi:10.1371/journal.pgen.1003754)
Supplement: Table S2 — Influence of founding haplotype stringency on founding haplotype assignment. The proportion of the genome in which we infer less than two founding haplotypes and no ambiguity in founding haplotype assignment as we change the X consecutive SNPS spanning Y base pairs required for founding haplotype assignment. (PDF) [file pgen.1003754.s012.pdf]

Table S2: Summary of haplotype information across haplotype calling cutoffs

X_{SNP}	Y_{BP}	unambiguous and < 3 haplotypes	ambiguous and/or > 2 haplotypes
2	10 bp	0.77	0.23
4	10 bp	0.70	0.30
10	10 bp	0.58	0.42
2	1 kb	0.76	0.24
4	1 kb	0.70	0.30
10	1 kb	0.58	0.42
2	10 kb	0.72	0.28
4	10 kb	0.67	0.33
10	10 kb	0.57	0.43
2	100 kb	0.54	0.46
4	100 kb	0.53	0.47
10	100 kb	0.49	0.51
