# Supplementary material for: Genomic Identification of Founding Haplotypes Reveals the History of the Selfing Species Capsella rubella
Source: PLoS Genet. 2013 Sep 12;9(9):e1003754. doi: 10.1371/journal.pgen.1003754 (PMC3772084; doi:10.1371/journal.pgen.1003754)
Supplement: Table S3 — Summary of synonymous and non-synonymous variation within and among C. rubella's founding haplotypes. All = comparison between all C. rubella samples, G/G = comparison between two C. rubella samples from Greece, G/O = comparison betweenGreek and Out-of-Greek C. rubella samples, O/O = comparison between two C. rubella samples from Out-of-Greece. (PDF) [file pgen.1003754.s013.pdf]

Table S3) Variation within and among *C. rubella* haplotypes:

	all	G/G	G/O	O/O
$100 \times \pi_S$% within haps	5.29 [5.11-5.40]	5.55 [5.24-5.84]	6.05 [5.78-6.24]	3.48 [3.20-3.66]
π_S% among haps	2.01 [1.98-2.04]	2.02 [1.97-2.05]	2.00 [1.96-2.03]	2.03 [2.00-2.07]
π_N/π_S within haps	.438 [.425,.449]	.416 [.392,.441]	.439 [.422,.458]	.461 [.426,.498]
π_N/π_S among haps	.140 [0.139,0.142]	.139 [.137,.141]	.139 [.139,.140]	.137 [.135,.139]
