# Supplementary material for: Genomic Identification of Founding Haplotypes Reveals the History of the Selfing Species Capsella rubella
Source: PLoS Genet. 2013 Sep 12;9(9):e1003754. doi: 10.1371/journal.pgen.1003754 (PMC3772084; doi:10.1371/journal.pgen.1003754)
Supplement: Text S1 — Supplementary text: Additional details about the data and analyses presented above. Data summary without reference to haplotype. The test shows no signal of recent introgression. Founding haplotype sharing by physical distance. Validation of RNA-Seq genotypes by Sanger sequencing. Robustness of results to thresholds for calling haplotype. A summary of diversity within and among founding haplotypes. and major haplotype frequency of the across all chromosomes. Putatively allozygous regions. Third haplotype candidate regions. (PDF) [file pgen.1003754.s014.pdf]

Contents

1 Data summary without reference to haplotype: 2

2 The f_3 test shows no signal of recent introgression 2

3 Founding haplotype sharing by physical distance 2

4 Validation 3

4.1 Potential influence of errors on inference 3

5 Robustness of results to haplotype calling cutoffs 4

5.1 Haplotype assignment and haplotype sharing: 5

5.2 Summary statistics 5

5.3 Inference 6

6 A summary of diversity within and among founding haplotypes 7

7 π_S and major haplotype frequency of the across all chromosomes: 8

8 Allozygous regions 8

9 Third haplotype candidate regions 9

1 Data summary without reference to haplotype:

We present basic data summaries without reference to our haplotype-based method in Figure S1, as discussed in the main text.

2 The f_3 test shows no signal of recent introgression

To further investigate the possibility of recent introgression in Greece, we made use of the f_3 statistic [1, 2]. This test compares the covariance of allele frequencies in three populations to identify a signal of gene flow [1, 2]. $f_3(c; a, b)$ is formally defined as $E[(c' - a')(c' - b')]$, where the prime denotes allele frequencies in putatively admixed population, c and putative source populations, b and c, and can only be negative if population c is a mixture of populations closely related to populations a and b. We found f_3(Greek; *C. grandiflora*, Out-of-Greece) to be significantly greater than zero (0.35 [0.32,0.38]), and therefore lack evidence for recent admixture between Greek *C. rubella* and *C. grandiflora*.

3 Founding haplotype sharing by physical distance

In the main text, we presented summaries of distances and proportions of founding haplotype sharing. In those analyses, we measured distance on a genetic map generated in a *C. rubella* x *C. grandiflora* interspecific cross. In Figure S2 we show that our summaries of founding haplotype sharing qualitatively hold when measuring in physical, rather than genetic distance. Across all pairwise comparisons, we observe a slightly higher proportion of the genome inheriting the same founding haplotype when comparing within Greece, as compared to between Greek and Out-of-Greece samples, while we see the most sharing of founding haplotypes in comparisons between Out-of-Greece samples.

4 Validation

We compared our genotype calls to $\approx$ 53 Kb of Sager sequencing (see Table S1A for sequenced regions / effort) to empirically investigate the error rate of our data. On the whole, there was little discordance between sequences (2 miscalls 52306 bp, an error rate more than an order of magnitude lower than π_S within founding haplotypes), and π for both data types was remarkably similar. In Table S1B we present a summary of our comparisons between RNA-Seq and Sanger sequencing. The two miscalls both occurred in our Argentinean sample. Both of these samples were run on the same and both of which exhibited higher levels of heterozygosity in putatively autozygous regions than the other individuals (Figure S8, below), constant with a lane effect on sequencing quality. Below we discuss how this potential lane effect on error rate could influence inference.

4.1 Potential influence of errors on inference

The potentially higher error rate in our Argentinian and Algerian samples has relatively little influence on our major conclusions. These two samples were not used in our demographic models, and the (still low) error rate is too low to have a substantial influence on genome-wide diversity measures. Additionally, since sequencing errors are likely overwhelmingly singletons, they are unlikely to influence our haplotype labeling, which makes use of common variants shared across species.

However, such sequencing errors could influence two summaries of diversity within founding haplotypes in the Out-of-Greece samples:

1. **Overestimate of Out-of-Greece growth:** We observed an excess of singletons in Out-of-Greek samples residing the same founding haplotype, suggesting recent growth and/or significant population structure Out-of-Greece (main text). However, this results is also consistent with sequencing error, which generates singletons, and therefore some of this signal may be due to sequencing error. Thus, although we have clear evi-

dence for an Out-of-Greece history of *C. rubella*, the rate of population growth and/or population structure outside of Greece is unclear.

2. **Overestimate of π_N/π_S within haplotypes in Out-of-Greece samples:** Within founding haplotypes, diversity at synonymous relative to non synonymous sites (π_N/π_S) increases with the number of Out-of-Greece samples. Since sequencing error is expected to target sites without respect to their degeneracy, while purifying selection is expected to eliminate deleterious mutations, sequencing error can increase π_N/π_S and therefore may contribute to the high π_N/π_S observed outside of Greece.

In summary, the pattern of potential sequencing error may change some details of the diasporan history *C. rubella* but does not strongly influence our major findings regarding the history of *C. rubella*.

5 Robustness of results to haplotype calling cutoffs

In the *METHODS*, we describe our algorithm for haplotype assignment. This algorithm requires us to prescribe threshold values for the number of consecutive SNPs and the distance in base pairs required to assign individuals to the same founding haplotype (i.e. in our pairwise assignment we insist that two individuals are identical at sites polymorphic in both species for X_{SNP} over at least Y_{BP}). We then combined information across individuals to create 'higher order assignments,' where we assigned all individuals to the same founding haplotype when there was no joint polymorphism for ten kb and five SNPs. Here we show that our major conclusions are robust to these cutoffs by demonstrating that inference is consistent across a diversity of pairwise combinations of $X_{\text{SNP}} = \{2, 4, 10\}$ and $Y_{\text{BP}} = \{10, 10^3, 10^4, 10^5\}$).

While all major results hold across all parameters investigated, some of the details change. Below, we discuss how these change what influence our parameters have on some informative

summary statistics, and how these results alter the interpretation of our findings.

5.1 Haplotype assignment and haplotype sharing:

As the criteria for assigning individuals to the same or different founding haplotype became stricter (e.g. X_{SNP} and/or Y_{BP} increased), proportionately less of the genome provided clean haplotype calls (exactly one or two founding haplotypes), while more of the genome yielded ambiguous haplotype calls and/or was inferred to contain more than two founding haplotypes (Table S2). These results are consistent with expectations, increasing the stringency necessary to assign individuals to founding haplotypes left us with fewer regions where individuals can be assigned to founding haplotypes. This expected effect also influences the portion of samples assigned to the same or different founding haplotypes across geographic comparisons (Figure S3, compare to Figure 2A in the main text).

5.2 Summary statistics

In Figures S4 and S5, we present basic summaries of variation within and among founding haplotypes across haplotype labeling cutoffs.

We present all three-way allele frequency spectra within haplotypes averaged across geographic comparisons in Figure S4. Note that, although some results change slightly with haplotype calling rules, results are relatively stable and consistently separated from both the standard neutral expectations and the allele frequency spectrum without reference to founding haplotype.

We present π_S and π_N/π_S within and among founding haplotypes in Figure S5. Although results are relatively consistent across parameters, there are a few noteworthy trends.

1. **Same haplotype:** Insisting on strict criteria to assign chromosomes to the same founding haplotype results in these regions being very recently diverged (e.g. Figure 3B), and, as expected decreases π_S within founding haplotypes (S5A). This recency

appears to also result in less time for putatively deleterious mutations to be removed from the population, increasing π_N/π_S within founding haplotypes (S5A).

2. **Different haplotype:** Perhaps counterintuitively, increasing the length for which two samples must differ at sites polymorphic in both species also decreases π_S and π_N/π_S between founding haplotypes (S5B). This result could be due to a slight enrichment of regions in which all samples reside on the same founding haplotype too short to be caught by our *ad-hoc* rules. However, diversity among founding haplotypes is orthogonal to our major questions and inferences, and therefore this result does not influence our major claims in any way.

5.3 Inference

Above, we showed that most of our summary statistics do not change, or change slightly and predictably across founding haplotype calling thresholds. Here, we review our three main results concerning the history or *C. rubella* gleaned from our coalescent model and investigate how founding haplotype assignment cutoffs influence these conclusions

1. **No need to postulate an extreme bottleneck:** In the main text, we showed that while we could not completely rule out an 'extreme' founding of *C. rubella*, we had little evidence supporting this hypothesis. We arrive at a similar conclusion for most founding haplotype calling rules (Figure S6A); however, when only exceptionally long regions can be assigned to founding haplotypes, our model begins to favor an extreme founding event. This result is expected – as we limit the regions assigned to founding haplotypes these regions will seem young and long and will trace their ancestry to few founders. Since even under these standards, a large number of founders is still likely, and since this extreme method of haplotype calling is expected to generate this bias, we find little compelling evidence for an 'extreme' founding event.

2. **Reduced long term effective population size:** We infer a very small effective population size ($N_0 = \{25,000 - 40,000\}$) across all haplotype labeling cutoffs. We find a decrease in the inferred N_0 with the stringency required to assign samples to founding haplotypes. Two potential factors likely generate this pattern

   (a) Shared ancestry across long distances suggests recent common ancestry and hence less time for mutations to accumulate (a result observed in this data, but not presented), decreasing π_S.

   (b) Lower stringency may accidentally place samples on the same haplotype, artificially increasing estimates of π_S.

3. ***C. rubella* originated ≈ 50 kya:** Estimates of the date of origin of *C. rubella* vary slightly across founding haplotype calling threshold for reasons similar to those listed above. The 95% confidence intervals are partially overlapping for every date estimate provided. Note that these confidence intervals are larger than those provided in the main text because here we do not constrain our initial population size to be the MLE. We note that the variation in our estimates induced by our haplotype labeling rules pales in comparison to our uncertainty in the mutation rate. If we replace the estimate of μ/gen of 1.5×10^{-8}, with an alternative estimate of 7×10^{-9} we double our estimated split time (see main text).

6 A summary of diversity within and among founding haplotypes

Figures 3A and 3B , we display π_S and π_N/π_S within and among *C. rubella's* founding haplotypes. In table S3, we present these values and the associated bootstrapped confidence intervals.

7 π_S and major haplotype frequency of the across all chromosomes:

In the main text we present the relationship between nucleotide diversity and haplotype frequency for chromosome seven (Figure 5). We present similar results across all chromosomes in 10 kb windows with a 2 kb slide, below (Figure S7). As in Figure 5, synonymous diversity is in purple, the inferred major haplotype frequency is in orange, and red points putatively containing more than two founding haplotypes. Also, like Figure 5, nucleotide diversity increases as major founding haplotype frequency decreases.

8 Allozygous regions

We present individual heterozygosity at synonymous sites in putatively allozygous and autozygous genomic regions in Figure S8. Diversity in allozygous regions closely matches diversity between individuals, as expected if regions that we infer to be allozygous are correctly identified. While individual heterozygosity is clearly higher in allozygous than in autozygous regions, we still observe heterozygous genotypes in putatively autozygous regions. We treat these heterozygous sites in putatively autozygous regions as missing data since hey likely represent sequencing errors, and we point out that they are overrepresented and are predominantly singletons in the Algerian and Argentinian samples (consistent with the potential lane effect on error rate, described above).

We label allozygous regions by eye (Figures S9A-F) for each individual – inferring a region to be allozygous when the slope of the cumulative number of heterozygous sites on physical position is relatively large. We exclude these putatively allozygous regions from all haplotype-based analyses. Sites heterozygous in all *C. rubella* samples are censured in *C. grandiflora* and *C. rubella* analyses, as they likely represent common misalignments.

9 Third haplotype candidate regions

We display nine regions likely to contain more than three founding haplotypes in Figure S10. In each panel we present π_S between all combinations of three individuals inferred to have inherited alternative founding haplotypes (across 10 kb windows each overlapping by 2 kb). When π_S between each individual in the trio is high (i.e. near the level of d_U – the dashed horizontal line) it is likely that the three individuals have inherited distinct founding haplotypes. We also display π_S within a founding haplotype in grey – the small values of these grey lines argues against the possibility that these regions are poorly aligned. We note that this non-random sample of our 172 candidate regions was chosen to argue that some of these regions are likely correct, and it is therefore that *C. rubella* originated from a single founder without subsequent introgression. We also note that since π_S between some samples is very low (grey lines), these regions are not easily dismissed as likely alignment errors.

References

[1] Nick Patterson, Priya Moorjani, Yontao Luo, Swapan Mallick, Nadin Rohland, Yiping Zhan, Teri Genschoreck, Teresa Webster, and David Reich. Ancient admixture in human history. *Genetics*, 192:1065–1093, 2012.

[2] David Reich, Kumarasamy Thangaraj, Nick Patterson, Alkes L Price, and Lalji Singh. Reconstructing indian population history. *Nature*, 461:489–494, 2009.
